# Proteogenomic Analysis of HDAC4 and HDAC5 in Uveal Melanoma

**DOI:** 10.1186/s10020-026-01652-9

**Published:** 2026-09-22

**Authors:** Svenja Henning, Tanja J. Kutzner, Martin Schneider, Teresa González-Gimeno, Yue Zheng, Utta Berchner-Pfannschmidt, Dominic Helm, Bernd Giebel, Nikolaos E. Bechrakis, Samuel Peña-Llopis

**Affiliations:** 1 Translational Genomics, German Cancer Consortium (DKTK) and Department of Ophthalmology, University Hospital Essen, 45147 Essen, Germany; 2 German Cancer Consortium (DKTK) and German Cancer Research Center (DKFZ), Heidelberg, Germany; 3 Institute for Transfusion Medicine, University Hospital Essen, University of Duisburg-Essen, Essen, Germany; 4 Proteomics Core Facility, German Cancer Research Center (DKFZ), Heidelberg, Germany; 5 Department of Ophthalmology, University Hospital Essen, Essen, Germany; 6 Faculty of Experimental Sciences, Universidad Francisco de Vitoria (UFV), Madrid, Spain

**Keywords:** Choroid Melanoma, Histone Deacetylases, HDAC

## Abstract

Uveal melanoma (UM) is a rare but aggressive cancer arising from cells of the uveal tract that is highly resistant to therapy once it has metastasized. In drug screening experiments, we identified a high sensitivity of primary UM cell lines to the pan-histone deacetylase (HDAC) inhibitor panobinostat and addressed the question whether two class IIa HDACs, HDAC4 and HDAC5, could serve as potential therapeutic targets in UM. Selective pharmacological inhibition of HDAC4/5, as well as stable knockdown of *HDAC4* or *HDAC5,* did not strongly affect cell viability, differentiation, apoptosis or necrosis, and did not sensitize cells to other HDAC or MEK inhibitors. Proteogenomics analysis revealed 73% concordance and only 7% discordance in the expression of transcripts and proteins. Concordance reached 92% for significant transcripts. Despite the lack of severe global gene and protein expression changes in *HDAC4-* or *HDAC5-*knockdown UM cell lines, the expression of individual cancer-related genes and signaling pathways was differently regulated following *HDAC4* or *HDAC5* knockdown. This was particularly evident in the NF-κB pathway, which showed decreased NF-κB p65 phosphorylation in UM cell lines with monosomy in chromosome 3 (M3) genotype and mutations in *BRCA1-associated protein 1* (*BAP1*). In summary, our results suggest that HDAC4/5 inhibitors alone are insufficient for monotherapy in UM cells, but the identification of HDAC4/5-regulated genes involved in cancer progression provides a rationale for exploring combination strategies that include HDAC4/5 inhibition.

**Supplementary Information:**

The online version contains supplementary material available at https://doi.org/10.1186/s10020-026-01652-9.

## Introduction

Uveal melanoma (UM) is the most common intraocular malignancy in adults and arises from melanocytes in the uveal layer, which comprises the choroid, the ciliary body and the iris (Singh et al. 2005; 2007). Around 50% of UM cases progress to metastasis, leading to an overall survival rate of 4 to 7 months after metastatic disease diagnosis due to a lack of effective therapeutic options (Gragoudas et al. 1991; Bedikian et al. 1995; Diener-West et al. 2005). Monosomy of chromosome 3 (M3) is an important marker of metastasis and poor prognosis in UM. It is strongly correlated with mutations in the tumor suppressor gene *BRCA1-associated protein 1* (*BAP1*), which is located on chromosome 3p21 (Prescher et al. 1996; Harbour et al. 2010). However, disomy of chromosome 3 (D3) and mutations in *eukaryotic translation initiation factor 1A* (*EIF1AX*) or *splicing factor 3B subunit 1* (SF3B1) are associated with a better prognosis (Prescher et al. 1996; Harbour et al. 2013; Martin et al. 2013). Identifying prognostic signatures has led to the distinction of two UM classes. Tumors in class 1 (low grade) have a better prognosis than those in class 2 (high grade) (Onken et al. 2004). This UM classification was subsequently expanded to include four groups based on differential gene expression and DNA methylation profiles (Robertson et al. 2017).

As a nuclear deubiquitinase, BAP1 plays a role in regulating central cellular functions at the epigenetic level by influencing chromatin remodeling and regulating different transcription factors (Carbone et al. 2013). Although the mechanisms by which *BAP1* mutations promote tumor aggressiveness are not fully understood, it has been demonstrated that BAP1 loss impairs cellular differentiation and induces a stem cell-like state in UM cells (Kuznetsov et al. 2019; Matatall et al. 2013). This is accompanied by the downregulation of key pathway genes involved in melanocytic differentiation. Similarly, poor prognostic class 2 UM tumors show a stem cell-like gene expression profile that resembles primitive neural and ectodermal stem cells. In contrast, class 1 UM tumors are more similar to mature neural crest cells or differentiated melanocytes (Chang et al. 2008).

Despite current treatments, approximately half of UM patients experience distant metastases, primarily those with *BAP1* mutations (Harbour et al. 2010). Historically, the median survival rate for patients with metastatic UM was less than one year. However, it has improved modestly with newer therapies, especially tebentafusp for eligible patients (Nathan et al. 2021). Therefore, there is an urgent need for more effective therapies for UM.

Histone deacetylases (HDACs) are a family of enzymes that modulate gene transcription by removing acetyl groups from lysine residues on histone tails. As acetylation neutralizes the positive charge of lysine, acetylation of histones facilitates a relaxed chromatin structure and allows the access of transcription factors to the DNA. Conversely, histone deacetylation represses gene transcription, making HDACs important mediators of tightly controlled genetic transcriptional programs (Grunstein 1997). In humans, HDACs are classified into four families based on their homology to yeast analogs (Gregoretti et al. 2004). Several studies have demonstrated the important regulatory functions of HDACs in cell proliferation, migration, angiogenesis, and cellular differentiation (Marks and Xu 2009). These effects are mediated by modulation of chromatin structure through histone deacetylation and deacetylation of non-histone protein targets (Marks and Xu 2009). Aberrant HDAC expression and activity are linked to various human disorders, including cancer (Hontecillas-Prieto et al. 2020).

HDAC inhibitors (HDACi) have been shown to effectively reduce cell proliferation, induce apoptosis, and increase the susceptibility of cancer cells to other antitumor agents in combination therapy approaches (Hontecillas-Prieto et al. 2020). In UM, HDACi have been shown to induce cellular differentiation at the morphological and gene expression levels. They also have the ability to revert BAP1-loss-associated H2A hyperubiquitination, making BAP1-deficient UM cells more susceptible to HDACi than BAP1-wild-type cells (Landreville et al. 2012). A recent study investigating the effects of *Bap1* loss in *Xenopus laevis* development identified *Hdac4* as being significantly upregulated when *Bap1* was inactivated, and it was found that rescue of the Bap1-deficient phenotype was possible by simultaneous depletion of *Hdac4* as well as upon treatment with HDACi (Kuznetsov et al. 2019). Moreover, knockdown of *HDAC4* was shown to significantly decrease proliferation in *BAP1*-mutant UM cell lines, but not in *BAP1*-wild type cell lines (Kuznetsov et al. 2019). These findings make HDACi attractive candidates in the therapy of *BAP1*-mutant UM.

In this study, we aimed to identify novel therapeutic approaches for UM treatment. We analyzed the effects of various pharmacological compounds on the viability of primary UM cell lines and found that all were sensitive to pan-HDACi. Analysis of The Cancer Genome Atlas (TCGA) data from uveal melanoma (UVM-TCGA) revealed high expression of *HDAC4* and *HDAC5*, as well as a correlation between *HDAC4* expression and M3, *BAP1* mutations, and poor survival. We hypothesized that targeting HDAC4 would make UM cells with M3 and *BAP1* mutations more vulnerable, as recently reported (Kuznetsov et al. 2019). However, we did not detect sensitivity to HDAC4 pharmacological inhibition or changes in cell proliferation upon *HDAC4* knockdown in UM cell lines, regardless of *BAP1* mutational status. This calls into question the suitability of HDAC4i or HDAC5i as monotherapeutic options for UM treatment. Nevertheless, gene expression and proteomics analyses of *HDAC4/HDAC5* knockdown UM cells revealed the involvement of *HDAC4/HDAC5* in cancer-related pathways. This finding provides a rationale for investigating different potential combination therapy partners. Overall, our results demonstrate the limited effectiveness of specific HDAC4/HDAC5 inhibitors as monotherapies in UM cells, but they also encourage further investigation of potential combinatorial approaches.

## Methods

### Cell lines

Cell lines UPMD1, UPMD2, UPMM2, UPMM3 and UPMM4 were derived from untreated primary UM tumors and characterized by the Department of Human Genetics (Nareyeck et al. 2009), University Hospital Essen, and kindly provided as early passages by Dr. M. Zeschnigk after determination of their chromosome 3 status. Primary UM cells and cell line 92–1 were cultured as described previously (Nareyeck et al. 2009; Mergener et al. 2021).

### Semi-automatic drug screening

Compounds (Supplementary Table S1) were dissolved to concentrations of 10 mM in DMSO and printed at nine concentrations ranging from 5 nM to 25 µM in triplicate in white, sterile, polystyrene, 384-well plates (Corning, 3570) with a Tecan D300e Digital Dispenser (Tecan, Switzerland/HP Inc., CA, USA) and the corresponding software D300e Control (v3.1.3), as previously described (Mergener et al. 2021). DMSO without compound was printed as negative controls. For library compounds, triplicates of 1 µM or 10 µM (or 100 nM and 1 µM, as indicated in Supplementary Figure S4) were used. Two drug libraries of highly selective and well characterized inhibitors were obtained from the Structural Genomics Consortium (SGC) chemical probe program (epigenetic library) or from donations from industry (donated chemical probes, DCP), respectively. Selectivity of all library compounds is specified in the respective corresponding online databases (https://www.sgc-ffm.uni-frankfurt.de/ and https://www.thesgc.org/sgc-chemical-probes). To ensure capturing the linear growth phase of cells during drug treatment, cell number and assay period were optimized prior to treatment experiments by seeding ten different cell numbers between 100 cells per well and 3,000 cells per well of each cell line in 30 µL of cell culture medium as triplicates in 9 replicate assay plates and performing cell viability assay (Mergener et al. 2021) every 24 h for 8 days (Supplementary Figure S1). Cell proliferation was analyzed by normalization of luminescence values to day 0. Based on these analyses, we selected a seeding density of 500 cells per well and an assay duration of five days for all subsequent experiments. For drug screening experiments, an optimal amount of 500 cells per well was seeded in 30 µL culture medium with a Multidrop Combi dispenser (Thermo Fisher Scientific, MA, USA). The assay plates were then incubated at 37 °C and 5% CO_2_ for 120 h (5 days) as the treatment time. As a readout, cell viability assay was performed as previously described (Mergener et al. 2021).

To assess synergistic effects, half maximal inhibitory concentrations (IC_50_), defined as the concentration resulting in *y* = 50% cell viability (”absolute “ IC_50_), of trametinib and panobinostat were determined for each cell line and individual concentration ranges were calculated by using the eight-fold IC_50_ as the maximum and one-eighth of the IC_50_ as the minimum dose. For tasquinimod, a similar concentration range as in the initial screening experiments was used for all cell lines due to non-determinable individual IC_50_ values. Compounds were printed as a matrix in duplicate concentration pairs in 384-well plates and assay was performed as formerly stated. Data was analyzed in Microsoft Excel (v16.0.13801.20288) and Combenefit (Veroli et al. 2016) (v2.021) with selection of the Loewe model (Loewe 1953; Loewe and Muischnek 1926).

### Construction of lentiviral shRNA vectors and transduction of UM cell lines

For stable shRNA expression, lentiviral particles were generated in HEK-293T cells cultured in DMEM medium (Thermo Fisher Scientific, 41,965,062) supplemented with 10% (*v/v*) fetal bovine serum (FBS, Life Technologies, 10,500,064) and 1% (*v/v*) Penicillin–Streptomycin (Thermo Fisher Scientific, 15,140,122), as previously described (Vega-Rubin-de-Celis et al. 2025). HEK-293T cells were transfected with pMD2.G envelope plasmid (Addgene, 12,259), psPAX2 packaging plasmid (Addgene, 12,260) and pLKO.1 vectors containing shRNA encoding sequences against *HDAC4* or *HDAC5* (Supplementary Table S2) and a puromycin resistance gene. As controls, an empty vector (for transduction of cell line 92–1) or vectors with scrambled sequences (pLKO.1-Sc for cell lines UPMM2 and UPMM3 and pLKO.1-Sc-#2 for transduction of cell line UPMD2, Supplementary Table S2) were used. Virus-particle containing supernatants were collected and passed through 0.45 µm syringe filters (Corning, 431,220) before using. For the transduction of UM cell line cells, cell culture media were replaced with filtered virus-particle containing supernatants. Successfully transduced cells were selected with 0.36 µg/mL (92–1) or 2 µg/mL (UPMD2, UPMM2, UPMM3) puromycin (Life Technologies, A1113803).

### Western blot

To evaluate knockdown efficiency, protein expression of HDAC4 and HDAC5 was evaluated by western blot, which was performed as described previously (Mergener et al. 2021; Niersch et al. 2021). Briefly, after blocking, membranes were incubated overnight at 4 °C in the presence of monoclonal rabbit anti-HDAC4 antibodies (Cell Signaling, 7628S) or monoclonal mouse anti-HDAC5 antibody (Santa Cruz, sc-133106), diluted at a ratio of 1:1000 or 1:500, respectively. Subsequently, membranes were washed in TBS-T (500 mM Tris, 1.5 M NaCl, 0.1% Tween-20, pH 7.6) and incubated in the presence of HRP-conjugated polyclonal goat anti-rabbit IgG secondary antibody (Jackson Immuno-Research, 111–035-003) or in that of HRP-conjugated polyclonal goat anti-mouse IgG secondary antibody (Jackson ImmunoResearch, 115–035-003), respectively, diluted 1:2000 for 1 h at room temperature. The HRP-conjugated monoclonal mouse anti-β-Actin (C-4) antibody (Santa Cruz, sc-47778-HRP) or the monoclonal mouse α-Tubulin antibody (Sigma, T6074) was used as a loading control.

### Proliferation assay and colony formation assay

To assess differences in proliferation and colony formation ability in cells of knockdown cell lines, respective assays were performed as previously described (Mergener et al. 2021). In brief, 500 cells per well were seeded in 30 µL of culture medium in triplicates in white-bottom polystyrene 384-well plates (Corning, 3570) in seven replicate plates per experiment and incubated at 37 °C and 5% CO_2_, followed by subsequent cell viability assay every 24 h for 7 days and normalization of values to day 0 for evaluation of proliferation. For colony formation assay, 1,500 cells (92–1) or 3,000 cells (UPMD2, UPMM2, UPMM3) per well were seeded in 2 mL culture medium in triplicates in 6-well culture plates (Starlab, CC7682-7506) and incubated for 2 weeks at 37 °C and 5% CO_2_, followed by methanol fixation and crystal violet staining. Air-dried plates were scanned using an Epson Perfection V850 Pro Scanner (Epson, Suwa, Japan) and confluency was quantified with the ImageJ-Plugin “Colony Area” (Guzman et al. 2014) followed by analysis in Microsoft Excel (v16.0.13801.20288) and GraphPad Prism 9 (v9.3.1).

### Wound healing assay

A wound healing assay was conducted on fast-growing 92–1 cells with *HDAC4* and *HDAC5* knockdown. Cells were seeded at a density of 50,000 cells/well in 24-well plates and grown for 48 h until confluent. Then, 24 h before scratching, the medium was replaced with RPMI containing 5% FBS to minimize proliferation and ensure that gap closure primarily reflects cell migration rather than cell division. Next, a scratch wound was introduced using a sterile 200-µL pipette tip. Cell debris was removed by washing with PBS, and then fresh RPMI with 5% FBS was added. Bright-field images were acquired at 10 × magnification at 0, 4, 24, 48, and 72 h after scratching using a Leica DMIL LED Fluo inverted microscope. The wound area was manually outlined using Fiji/ImageJ, and gap closure was calculated as follows: Gap closure (%) = [Area t = 0 − Area tN]/Area t = 0 × 100. For each condition and time point, the average and SD were calculated across 4 technical replicates. Three independent biological experiments were performed. The 0 h, 24 h, and 48 h timepoints were assessed in all three experiments. The 4 h timepoint was assessed in two experiments (replicates 1 and 2), and the 72 h timepoint was assessed in two experiments (replicates 1 and 3). We quantified genotype differences using a three-factor analysis of variance (ANOVA) with a Student–Newman–Keuls (SNK) post hoc test in IBM SPSS Statistics 29.0.

### The Cancer Genome Atlas (TCGA) analysis

RNA-Sequencing (RNA-Seq) data from 80 UM tumors from The Cancer Genome Atlas (UVM-TCGA) were acquired from the Genomic Data Commons (GDC) portal on 19 December 2020 (https://portal.gdc.cancer.gov). *BAP1* mutation status and survival data (overall and progression-free) were retrieved from Robertson and colleagues (Robertson et al. 2017), as described previously (Mergener et al. 2021). Six tumors without mutations in *BAP1* but with monosomy of chromosome 3 were excluded, which were considered uncertain for BAP1 inactivation (Mergener et al. 2021). Gene expression in UVM-TCGA data was analyzed as previously described (Mergener et al. 2021; Peña-Llopis et al. 2011). Briefly, gene expression was normalized using the RNA-Seq Expectation–Maximization (RSEM) method and normalized values were log2-transformed. Unpaired *t*-tests between tumors with or without *BAP1* mutations were performed considering the group variances, and the calculated *P* values were corrected by applying the Benjamini and Hochberg false discovery rate (FDR) method. Genomic-clinical associations were performed as previously reported (Peña-Llopis et al. 2016). The median and quartiles for the expression of *HDAC4* and *HDAC5* gene were calculated for all available patients of UVM-TCGA (*n* = 80). Gene expression was considered low for patients with expression values in the 1 st quartile, intermediate for the 2nd and 3rd quartiles and high for the 4th quartile. Kaplan–Meier survival curves and log-rank tests were calculated with IBM SPSS Statistics v29 (IBM, Ehningen, Germany).

For comparing *HDAC* expression levels in the 33 most prevalent cancer entities, gene expression data from the TCGA Pan-Cancer atlas (TCGA-PANCAN) was obtained and analyzed with the UCSC Xena platform (https://xena.ucsc.edu).

### Gene expression analyses

RNA was extracted from parental UM cell lines and *HDAC4/5* knockdown cells using the AllPrep DNA/RNA Kit (Qiagen, 80,204) according to the manufacturer’s protocol. RNA was quantified with a Nanodrop 2000c spectrophotometer (Thermo Fisher Scientific, MA, USA) and submitted in duplicate pairs to the Microarray Unit of the Genomics Core facility of the German Cancer Research Center (DKFZ), where gene expression profiling was performed using the Clariom S assay (Thermo Fisher Scientific, 902,927). To minimize possible shRNA-specific off-target effects in the analysis of *HDAC4/5* knockdown cells, we decided to analyze both *HDAC4* shRNA variants of each cell line, as well as the two *HDAC5* shRNA variants with the highest knockdown efficiency according to western blot analysis for each cell line (Supplementary Figure S10), and combined both variants of each cell line for *HDAC4* or *HDAC5* knockdown, respectively, for gene expression analysis. However, cell line 92–1 *shHDAC4 #2* was excluded due to insufficient knockdown of *HDAC4* (Supplementary Figure S10a).

### LC–MS based proteomics analyses

Cells were harvested with Protein Lysis Buffer containing protease and phosphatase inhibitors (Peña-Llopis and Brugarolas 2013) and treated with a final concentration of 10 U/ml DNase1 (Merck, 11,284,932,001) and 250 U/ml benzonase (Santa Cruz, sc-202391) for 1 h on ice. Samples were submitted in duplicate pairs to the Proteomics Core Facility at DKFZ. An amount of 10 μg of protein was run for 0.5 cm into an SDS-PAGE. After Coomassie staining the whole band was cut out and used for subsequent tryptic digestion according to Shevchenko and colleagues (Shevchenko et al. 2006), adapted to on a DigestPro MSi robotic system (INTAVIS Bioanalytical Instruments AG). LC–MS/MS analysis was carried out for a total of 150 min on an Ultimate 3000 UPLC system directly connected to an Orbitrap Exploris 480 mass spectrometer (both Thermo Fisher Scientific). Peptides were online desalted on a trapping cartridge (Acclaim PepMap300 C18, 5µm, 300Å wide pore; Thermo Fisher Scientific) for 3 min using 30 µl/min flow of 0.05% TFA in water. The analytical multistep gradient was carried out on a nanoEase MZ Peptide analytical column (300Å, 1.7 µm, 75 µm × 200 mm, Waters) using solvent A (0.1% formic acid in water) and solvent B (0.1% formic acid in acetonitrile). For 134 min the concentration of B was linearly ramped from 4 to 30%, followed by a quick ramp to 78%, after two minutes the concentration of B was lowered to 2% and a 10 min equilibration step appended. Eluting peptides were analyzed in the mass spectrometer using data depend acquisition (DDA) mode. A full scan at 120k resolution (380–1400 m/z, 300% AGC target, 45 ms maxIT) was followed by up to 2 s of MS/MS scans. Peptide features were isolated with a window of 1.4 m/z, fragmented using 26% NCE. Fragment spectra were recorded at 15k resolution (100% AGC target, 22 ms maxIT). Unassigned and singly charged eluting features were excluded from fragmentation and dynamic exclusion was set to 35 s.

Protein identification and quantification data analysis was carried out by MaxQuant (Tyanova et al. 2016) (version 1.6.14.0) using databases extracted from Uniprot.org under default settings (human containing 79,038 entries from 03.01.2022). Identification FDR cutoffs were 0.01 on peptide level and 0.01 on protein level. The match between runs (MBR) option was disabled. LFQ quantification was done using a label free quantification approach based on the MaxLFQ algorithm (Cox et al. 2014). A minimum of 2 quantified peptides per protein were required for protein quantification. For each cell line, separate parameter groups with separate LFQ normalization were used. LFQ values were further normalized via variance stabilization (Huber et al. 2002).

### Integration of transcriptomics and proteomics analyses

Quantile-normalized gene expression data were log2-transformed and analyzed as described above for UVM-TCGA (Mergener et al. 2021; Peña-Llopis et al. 2011). Principal Component Analyses (PCA) were performed for all transcripts/genes that were present in all samples using Partek Genomics Suite (v9.0, Partek, MO, USA). Proteomics samples with missing LFQ data were replaced by number two, log2-transformed and analyzed as described above for UVM-TCGA (Mergener et al. 2021; Peña-Llopis et al. 2011). Significant genes in transcriptomics and proteomics were further analyzed by Ingenuity Pathway Analysis (IPA) software (Qiagen, Germany) to identify overrepresented pathways upon knockdown of *HDAC4* or *HDAC5*.

### Flow cytometric analysis of apoptosis and necrosis

Apoptosis and necrosis were analyzed in the UM cell lines 92–1, UPMD2, UPMM2, and UPMM3 in technical triplicates. Cells (5 × 10^4^ per well) were seeded into six-well plates containing their corresponding medium. Apoptosis was induced by treatment with 1 µM staurosporine (Biomol) for 6 h at 37°C.

Cells were harvested by trypsinization and processed immediately. To recover all cells, including those detached during apoptosis or necrosis, the culture supernatant and the PBS wash were collected prior to trypsinization and combined with the trypsinized cell fraction. Cells were pelleted by centrifugation at 900 × *g* for 5 min and resuspended in 250 µL PBS. Cells were stained with APC-conjugated Annexin V (ImmunoTools) and 7-aminoactinomycin D (7-AAD; Beckman Coulter) for 20 min at 4 °C in the dark. Unstained control samples were processed in parallel. Following staining, cells were washed with PBS and resuspended in 100 µL PBS for flow cytometric analysis.

Samples were acquired on a CytoFLEX LX flow cytometer (Beckman Coulter, Krefeld, Germany). Data were analyzed using a uniform gating strategy for all samples. For cell death quantification, the total apoptotic population was determined by pooling early apoptotic (Annexin V^+^/7-AAD^−^) and late apoptotic (Annexin V^+^/7-AAD^+^) cells. Necrosis was defined by the presence of 7-AAD^+^ cells in the absence of Annexin V binding (Supplementary Figure S17). In both cases, the final percentages were calculated after subtraction of the background signal determined from unstained controls.

### Phospho-flow cytometric analysis of NF-κB p65

Phosphorylation of NF-κB p65 was analyzed in the UM cell lines 92–1, UPMD2, UPMM2, and UPMM3 by phospho-flow cytometry. Cells were harvested from 75 cm^2^ culture flasks and immediately fixed after detachment to preserve phosphorylation states. Fixation was performed using pre-warmed Phosflow™ Fix Buffer I (BD Biosciences, Heidelberg, Germany), which was incubated at 37 °C for 10 min prior to use. Cells were mixed with 100 µL fixative and incubated for 15 min at 37 °C.

Following fixation, samples were diluted to 15 mL with PBS, centrifuged (900 × *g*, 5 min), resuspended in 1 mL PBS, centrifuged again, and finally resuspended in 100 µL PBS. Cells were permeabilized by two washes with Perm/Wash Buffer I (BD Biosciences), each followed by centrifugation (500 × *g*, 5 min).

Intracellular staining was performed by incubating cells with an anti–phospho-NF-κB (p65) AF647-conjugated antibody (BD Biosciences) for 30 min at room temperature in the dark. AF647-conjugated IgG2b isotype controls and unstained controls were included and processed identically. After staining, cells were washed twice with Perm/Wash Buffer I and resuspended in 100 µL buffer prior to acquisition.

Data were acquired on a CytoFLEX LX flow cytometer (Beckman Coulter, Krefeld, Germany) (Supplementary Figure S22). Phosphorylated NF-κB p65 levels were quantified as mean fluorescence intensity (MFI).

### Statistics

Experiments were performed with *n* = 3–5 biological replicates per condition. Data were compared by unpaired *t* tests controlling for their variances unless otherwise indicated and adjusted with the Benjamini–Hochberg method for multiple testing. One-way, two-factor, and three-factor analysis of variance (ANOVA) with Student–Newman–Keuls (SNK) post hoc tests were calculated using IBM SPSS Statistics 29.0, where non-overlapping letters (e.g., “a” *vs*. “b”) represent significant differences (*P* < 0.05).

## Results

### UM cell lines are sensitive to HDAC inhibition

We systematically screened six uveal melanoma (UM) cell lines to evaluate their response to various pharmaceutically active compounds. Three of the cell lines had disomy of chromosome 3 (92–1, UPMD1, and UPMD2), and three had M3 and *BAP1* mutations (UPMM2, UPMM3, and UPMM4).

**Fig. 1:**
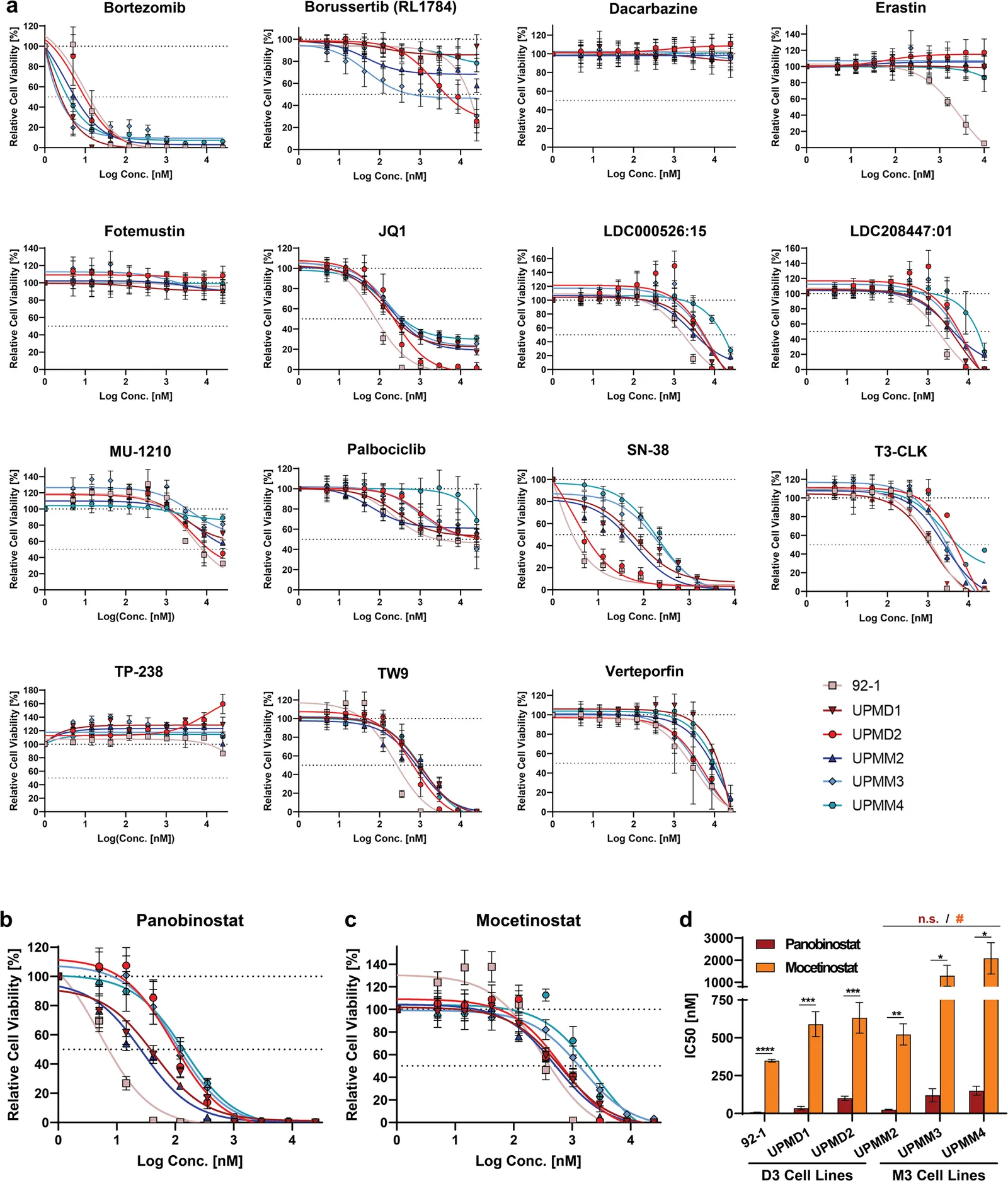
Drug screening experiments show that UM cell lines are more sensitive to the HDACi panobinostat compared to mocetinostat. Dose–response curves of uveal melanoma cell lines with M3 and *BAP1* mutations (blue symbols) or D3 and *BAP1* wild-type (red symbols) treated with a variety of different drugs (**a**), panobinostat (**b**) or mocetinostat (**c**) at different concentrations (from 5 nM to 25 µM) for 5 days. Cell viability is expressed as normalized values in percentage to DMSO controls. Summarized results of three independent experiments are shown. **d** Half maximal inhibitory concentrations (IC_50_) were determined for panobinostat and mocetinostat and compared in D3 *vs*. M3 cell lines. Data are average ± SD. *, *P* < 0.05; **, *P* < 0.01; ***, *P* < 0.001; ****, *P* < 0.0001 for the comparison of panobinostat *vs.* mocetinostat. #, *P* < 0.05 for the comparison of D3 cell lines with M3 cell lines

Building on our previous evaluation of trametinib, refametinib, carfilzomib, and sunitinib in these cell lines (Mergener et al. 2021), we expanded the screening to include 17 additional compounds spanning multiple inhibitor classes. These compounds were tested at nine different concentrations to generate full dose–response curves (Fig. 1, Supplementary Table S1), where IC_50_s and 95% CI were compared (Supplementary Figure S2). In parallel, we screened two curated drug libraries comprising 101 well-characterized compounds, each tested at two concentrations alongside 48 negative controls (Supplementary Figures S3 and S4).

Across all assays, most compounds exerted minimal effects on cell viability, revealing a pronounced and consistent resistance of UM cell lines to the majority of tested agents (Fig. 1, Supplementary Figures S3 and S4). In contrast, all UM cell lines were highly sensitive to bortezomib, a proteasome inhibitor that had shown more toxicity than efficacy in clinical trials (Markovic et al. 2005). Alternatively, the histone deacetylase inhibitors (HDACi) panobinostat and mocetinostat markedly reduced cell viability in all six UM cell lines. After five days of treatment, panobinostat induced a complete loss of viability at concentrations ranging from 100 nM to 1 µM, whereas mocetinostat achieved comparable effects at higher concentrations ranging from 1 µM to 10 µM (Fig. 1b,c).

Sensitivity to both HDAC inhibitors varied among cell lines, as reflected by distinct half-maximal inhibitory concentrations (IC_50_; Fig. 1d). Notably, panobinostat was consistently more potent than mocetinostat across all cell lines, with IC_50_ values that were sixfold lower (UPMD2) to 43-fold lower (92–1) (Fig. 1d). Furthermore, UM cell lines harboring M3 and *BAP1* mutations exhibited significantly increased resistance to mocetinostat, whereas their sensitivity to panobinostat remained unaffected (Fig. 1d).

### Class IIa HDACs are highly expressed in UM

**Fig. 2:**
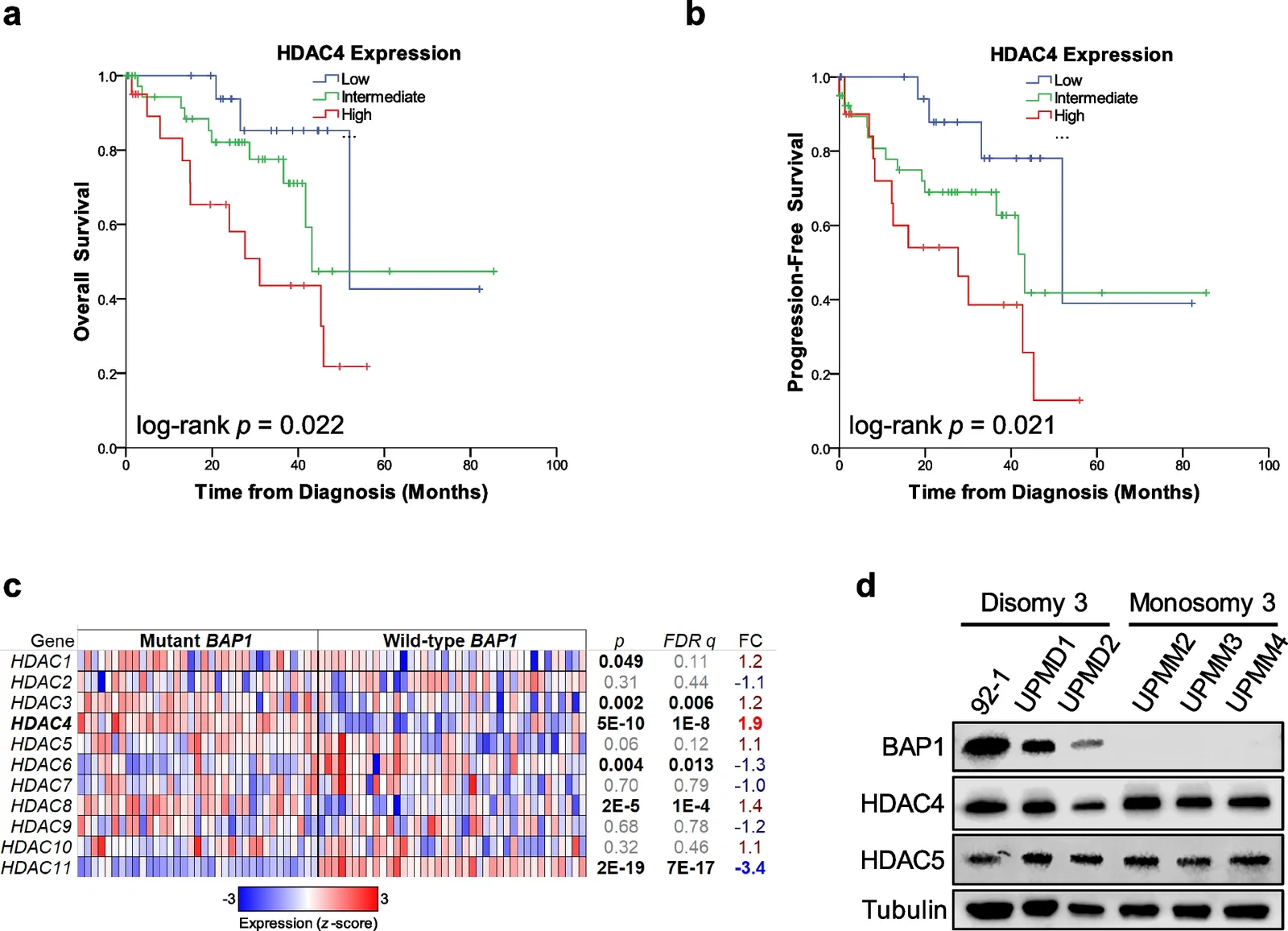
HDAC4 gene expression correlates with tumor aggressiveness in UM. **a/b** The Kaplan-Meier survival percentage estimate shows that high *HDAC4 *expression correlates with poor overall (**a**) and progression-free survival (**b**) in UVM-TCGA. Tumors (*n* = 80) were subdivided in three groups of high, intermediate or low gene expression based on quartiles. **c***BAP1*-mutant tumors from UVM-TCGA show significantly higher expression of *HDAC4* than tumors without mutations in *BAP1*. **d** Protein expression of HDAC4 and HDAC5 does not correlate with BAP1 status or M3 in UM cell lines as shown by western blotting. Un-cropped blots are displayed in Supplementary Figure S7. FDR, false-discovery rate corrected *P* value; FC, fold change

Because panobinostat and mocetinostat have different inhibitory activities due to their isotype specificity (mocetinostat does not inhibit class II HDACs, unlike panobinostat) (Zhou et al. 2008; Khan et al. 2007), we sought to investigate the role of class II HDACs in UM further. We focused on class IIa HDACs, specifically HDAC4, because class IIa HDACs have previously been described as playing important roles in regulating cellular differentiation and have been discussed in the context of their implications in cancer development (Parra 2015; Martin et al. 2007; Clocchiatti et al. 2011). Recent studies have described HDAC4 as potentially being involved in UM progression in the context of *BAP1* mutations and have proposed it as a key target in UM (Kuznetsov et al. 2019; Kuznetsoff et al. 2021). By comparing the gene expression of *HDAC1-11* in the TCGA Pan-Cancer (TCGA-PANCAN) dataset, we found that UM tumors (UVM-TCGA) had the highest expression of class IIa HDACs *HDAC4* and *HDAC5* among the 33 most prevalent cancers (Supplementary Figure S5). Further analyses confirmed an association of high expression of HDAC4, but not of *HDAC5*, with poor progression-free and overall survival in UM (Fig. 2a,b, Supplementary Figure S6), as well as a correlation between high *HDAC4* expression and a more aggressive UM subtype, as defined by M3 and BAP1 mutations in the UVM-TCGA data (Fig. 2c). Western blot analyses revealed high expression of HDAC4 and HDAC5 in all UM cell lines, but no correlation was observed with M3 or *BAP1* status at the protein level (Fig. 2d).

### UM cells do not exhibit strong sensitivity to the selective inhibition of HDAC4/5

**Fig. 3:**
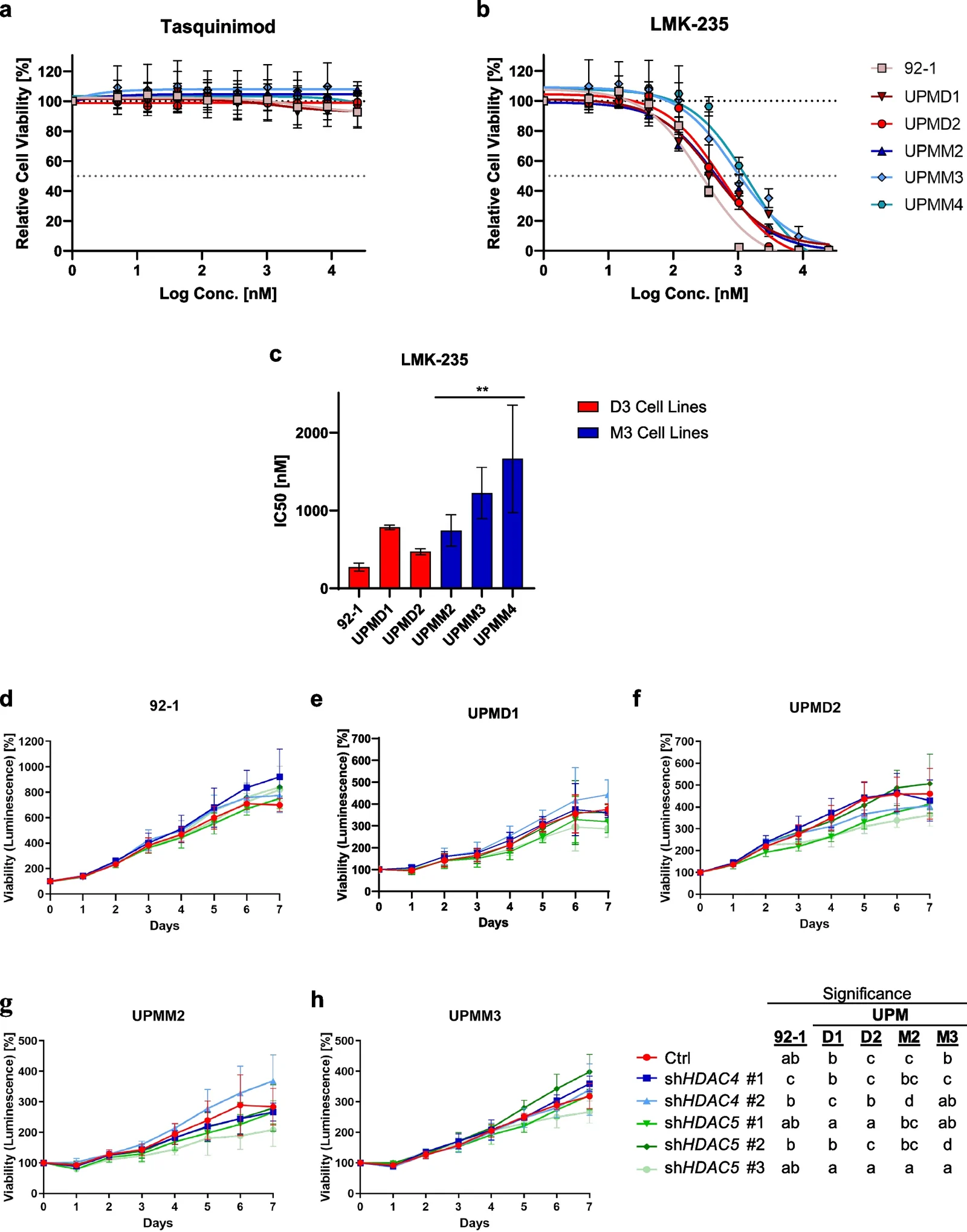
UM cell lines show no sensitivity to HDAC4 inhibition and moderate sensitivity to HDAC4/5 inhibition and are not significantly affected by *HDAC4* or *HDAC5* knockdown. Dose–response curves of UM cell lines with M3 and *BAP1* mutations (blue symbols) or D3 and *BAP1* wild-type (red symbols) treated with the HDAC4 inhibitor tasquinimod (**a**) or the HDAC4/5 inhibitor LMK-235 (**b**) at different concentrations (from 5 nM to 25 µM) for 5 days. Half maximal inhibitory concentrations (IC_50_) were determined for LMK-235 and compared in D3 *vs.* M3 cell lines (**c**). Cell viability is expressed as normalized values in percentage to DMSO controls. Summarized results of three independent experiments are shown. Cell viability rates of UM cell lines 92–1 (**d**), UPMD1 (**e**), UPMD2 (**f**), UPMM2 (**g**) and UPMM3 (**h**) upon knockdown of *HDAC4* or *HDAC5*. Luminescence is normalized to day 0 and expressed as percentage of day 0. Summarized results of three independent experiments are shown. Data are average ± SD. **, *P* < 0.01. Cell viability among different cell lines was quantified using a two-factor ANOVA and a Student–Newman–Keuls post hoc test. Non-overlapping letters (e.g., “a” *vs*. “b”) indicate significant differences (*P* < 0.05)

To investigate the effects of HDAC4 and HDAC5 inhibition in UM cells, we exposed all UM cell lines to the HDAC4 selective inhibitor tasquinimod and the HDAC4 and HDAC5 selective inhibitor LMK-235 for five days at concentrations up to 25 µM. Tasquinimod treatment did not result in detectable effects on cell viability, even at high concentrations, in any of the tested cell lines (Fig. 3a). LMK-235 treatment led to an intermediate response comparable to mocetinostat treatment in all cell lines, with complete reduction of cell viability at concentrations ranging from 1 µM to 10 µM and increased resistance in M3 cell lines (Fig. 3b,c). These results suggest that the significant decrease in cell viability observed following panobinostat treatment (Fig. 1b) may not primarily result from the inhibition of HDAC4 and HDAC5.

### Combined HDAC and MEK inhibition does not lead to strong synergistic effects in UM cell lines

Previously, it has been described that resistance to MEK inhibition (MEKi) in UM cells can be limited by the addition of the pan-HDAC inhibitor panobinostat (Faião-Flores et al. 2019). Since we reported an increased resistance to MEKi in M3 UM cell lines (Mergener et al. 2021), we aimed to investigate whether an additional HDACi could make M3 UM cells especially sensitive to MEKi. To accomplish this, we treated all UM cell lines with individual dose combinations of the MEKi trametinib and the pan-HDACi panobinostat centered on each drug''s and cell line''s respective IC_50_ for five days. We then analyzed the results using the Combenefit software (Veroli et al. 2016). We observed mild synergism, as calculated by the Loewe model (Loewe 1953; Loewe and Muischnek 1926), in single-concentration pairs. However, the observed synergism did not lead to a decrease in cell viability exceeding 10%, compared to single-drug treatment. The minimum remaining cell viability was 20% (Supplementary Figure S8). This indicates that resistance to trametinib cannot be circumvented by adding panobinostat in our UM cell lines in vitro, within the concentrations used.

Due to the absence of a response in all cell lines, we were unable to obtain IC_50_ concentrations for the selective HDAC4 inhibitor tasquinimod. Therefore, we assessed tasquinimod at concentrations up to 25 µM in MEKi synergy experiments but did not observe any synergistic effects (Supplementary Figure S9). As combinations of MEKi and selective HDACi targeting HDAC1/2/3 and HDAC6/8, respectively, have been shown not to exhibit synergistic effects (Faião-Flores et al. 2019), it is likely that a pan-HDACi is necessary to effectively suppress adaptive pathway activation in the presence of MEKi, and that selective HDACi alone is insufficient.

### Knocking down HDAC5, but not HDAC4, impairs UM cell growth

To better understand the roles of HDAC4 and HDAC5 as therapeutic targets in UM and to rule out the possibility that the tested selective compounds have insufficient inhibitory function in the investigated UM cell lines, we generated stable knockdown cell lines via lentiviral transduction of short hairpin RNAs (shRNAs) against *HDAC4* (two shRNA sequences) or *HDAC5* (three shRNA sequences) in four UM cell lines (92–1, UPMD2, UPMM2, and UPMM3). We then analyzed the phenotypic effects. We confirmed knockdown efficiency of at least 50% on the protein level by Western blotting in all cell lines except 92–1 sh*HDAC4* #2 (Supplementary Figure S10). Overall, we observed no consistent effects on cell morphology or strong changes in cell viability (Fig. 3d–h). *HDAC4* knockdown showed inconsistent results regarding cell viability. Only sh*HDAC4* #2 significantly impaired (*P* < 0.05) cell viability in UPMD2 cells. This was determined by comparing the slopes using a two-factor ANOVA followed by a Student–Newman–Keuls test. In contrast, *HDAC5* knockdown impaired cell viability in UPMD1, UPMD2, UPMM2, and UPMM3 cell lines, especially with sh*HDAC5* #3 (Fig. 3d–h).

*HDAC4* or *HDAC5* knockdown had various effects on colony formation. There was impaired colony formation ability upon *HDAC5* knockdown in the D3 cell lines 92–1 and UPMD2 but not in the M3 cell lines UPMM2 and UPMM3 (Supplementary Figure S12). Further effects on colony formation ability were restricted to single shRNAs and were therefore regarded as unspecific effects of the respective shRNA sequences, for example, upon *HDAC4* knockdown in UPMM2 (Supplementary Figure S12c) or *HDAC5* knockdown in UPMM3 (Supplementary Figure S12d).

### *Panobinostat efficacy is not recapitulated by* HDAC4 *or* HDAC5* knockdown*

**Fig. 4:**
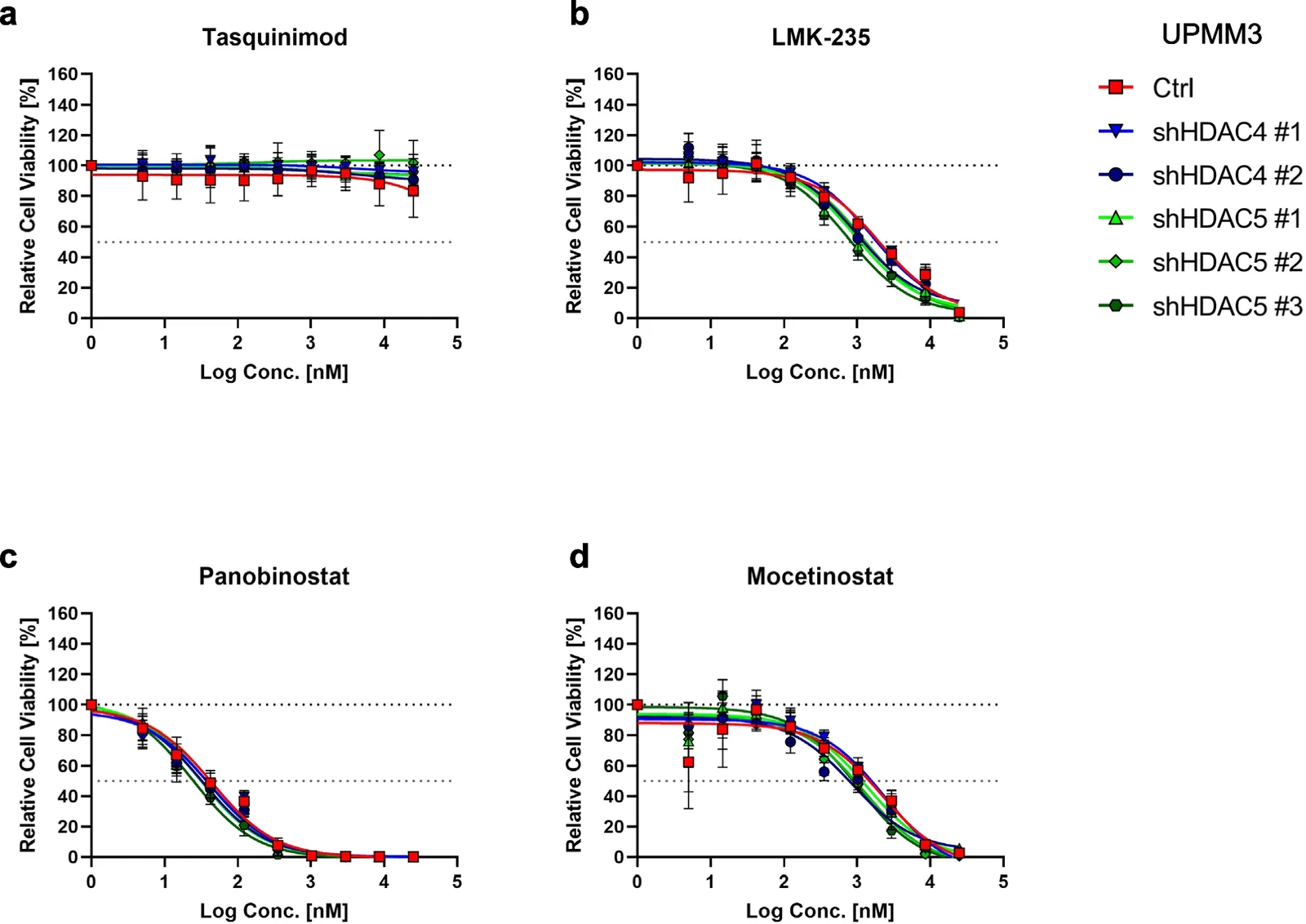
Sensitivity to HDAC inhibition is not driven by inhibition of HDAC4 or HDAC5. Dose–response curves of UM cell line UPMM3 upon shRNA knockdown of *HDAC4* or *HDAC5* with the HDAC4 inhibitor tasquinimod (**a**), the HDAC4/5 inhibitor LMK-235 (**b**), the pan-HDAC inhibitor panobinostat (**c**) or the HDAC inhibitor mocetinostat (**d**) at different concentrations (from 5 nM to 25 µM) for 5 days. Cell viability is expressed as normalized values in percentage to DMSO controls. Summarized results of two independent experiments are shown. Data are average ± SD. Similar results for cell lines 92–1, UPMD2 and UPMM2 are displayed in Supplementary Figure S13

To ascertain whether the marked diminution in cell viability engendered by the pan-HDAC inhibitor panobinostat could be ascribed to its concurrent suppression of HDAC4 and HDAC5, we investigated whether genetic repression of these targets would modify the cellular response to the class I–selective HDAC inhibitor mocetinostat. To this end, we repeated drug response experiments in UM cell lines with stable knockdown of *HDAC4* or *HDAC5*. Neither *HDAC4* nor *HDAC5* knockdown resulted in a strong or consistent shift in the mocetinostat response toward that observed with panobinostat across all tested cell lines (Fig. 4d, Supplementary Figure S13). These results suggest that the enhanced efficacy of panobinostat is not solely driven by the inhibition of HDAC4 or HDAC5.

Consistent with this interpretation, *HDAC5* knockdown cells failed to respond to the HDAC4-selective inhibitor tasquinimod (Fig. 4a, Supplementary Figure S13), whereas all cell lines remained sensitive to the HDAC4/5 dual inhibitor LMK-235 (Fig. 3b, 4b, Supplementary Figure S13). Together, these results suggest that suppression of individual class IIa HDACs is insufficient to reproduce the viability-reducing effects of pan-HDAC inhibition. They also point toward either compensatory pathway activation in knockdown cells or limited functional selectivity of the applied HDAC inhibitors.

### HDAC4 *and* HDAC5 *knockdown cells retain their respective D3 or M3 characteristics*

**Fig. 5:**
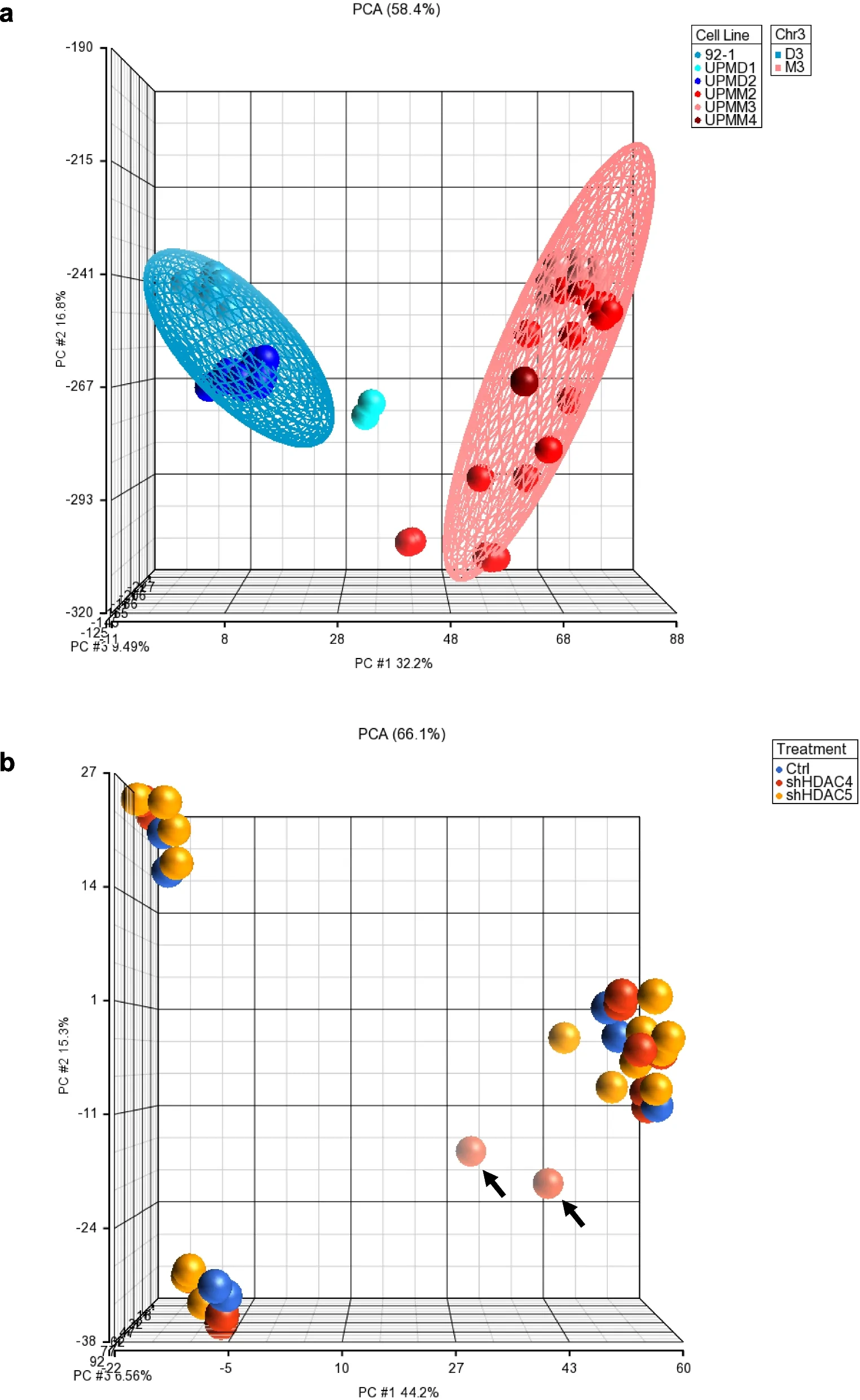
*HDAC4/5* knockdown cells retain their D3 or M3 characteristics and mostly cluster with their parental cell lines and controls. (**a**) Principal Component Analysis (PCA) demonstrates that knocking down *HDAC4* or *HDAC5* in cell lines 92–1, UPMD2, UPMM2, and UPMM3 does not affect their D3- or M3-specific gene expression profiles. All knockdown variants cluster with their parental cells except UPMM2, indicating minor changes in gene expression. (**b**) PCA shows that stable knockdown of *HDAC4* or *HDAC5* in cell lines 92–1, UPMD2, UPMM2 and UPMM3 does not cause strong changes in gene expression, clustering with their controls. Arrows indicate UPMM2 sh*HDAC4* #2

To get a clearer picture of possible effects caused by the knockdown of *HDAC4* or *HDAC5* that do not result in phenotypic changes and to identify possible combination therapy partners, we analyzed gene expression profiling of knockdown cell lines using Clariom S Microarrays (Affymetrix). Principal Component Analysis (PCA) of all parental UM cell lines and *HDAC4*/*HDAC5* knockdown variants of cell lines 92–1, UPMD2, UPMM2 and UPMM3 showed that all knockdown variants widely remain in their respective D3 or M3 cluster as defined by their 95% confidence limits of the ellipsoids in Fig. 5a, and that most of them closely resemble the expression profiles of their respective parental cell lines, except UPMM2 knockdown variants, which showed a higher variance (Fig. 5a). Additionally, we could not observe a biological replicate effect (Supplementary Figure S15) or a specific pattern of gene expression shift upon knockdown of *HDAC4* or *HDAC5*, suggesting that the effects of *HDAC4* or *HDAC5* knockdown are minor in the investigated cell lines compared to the genotype effects of each cell line (Fig. 5b). Notably, UPMM2 sh*HDAC4* #2 constitutes an outlier in the PCA (Fig. 5b, arrows). We observed morphological changes in all transduced UPMM2 variants compared to the parental phenotype, including the control, except in UPMM2 sh*HDAC4* #2 (Supplementary Figure S14). As UPMM2 showed to be a cell line of high plasticity that undergoes morphological changes after longer times in cell culture, we speculated that the process of transduction served as a source of stress that accelerated such morphological changes as we had observed previously in this cell line, including in the control. However, this morphological transition was delayed in sh*HDAC4* #2, where such changes occurred several weeks later (Supplementary Figure S14). For this reason, we suppose that the dropout of UPMM2 sh*HDAC4* #2 in PCA is mainly caused by the delayed transition observed at the time of sample acquisition rather than by effects specific to *HDAC4* knockdown. However, it is possible to speculate that *HDAC4* knockdown could have caused the delay of the morphological transition exclusively in UPMM2 sh*HDAC4* #2, as *HDAC4* knockdown was stronger in this variant than in UPMM2 sh*HDAC4* #1 (Supplementary Figure S10), where cells underwent morphological transition at the same time as the other variants (Supplementary Figure S14).

### Integrative analysis of transcriptome and proteome expression

**Fig. 6:**
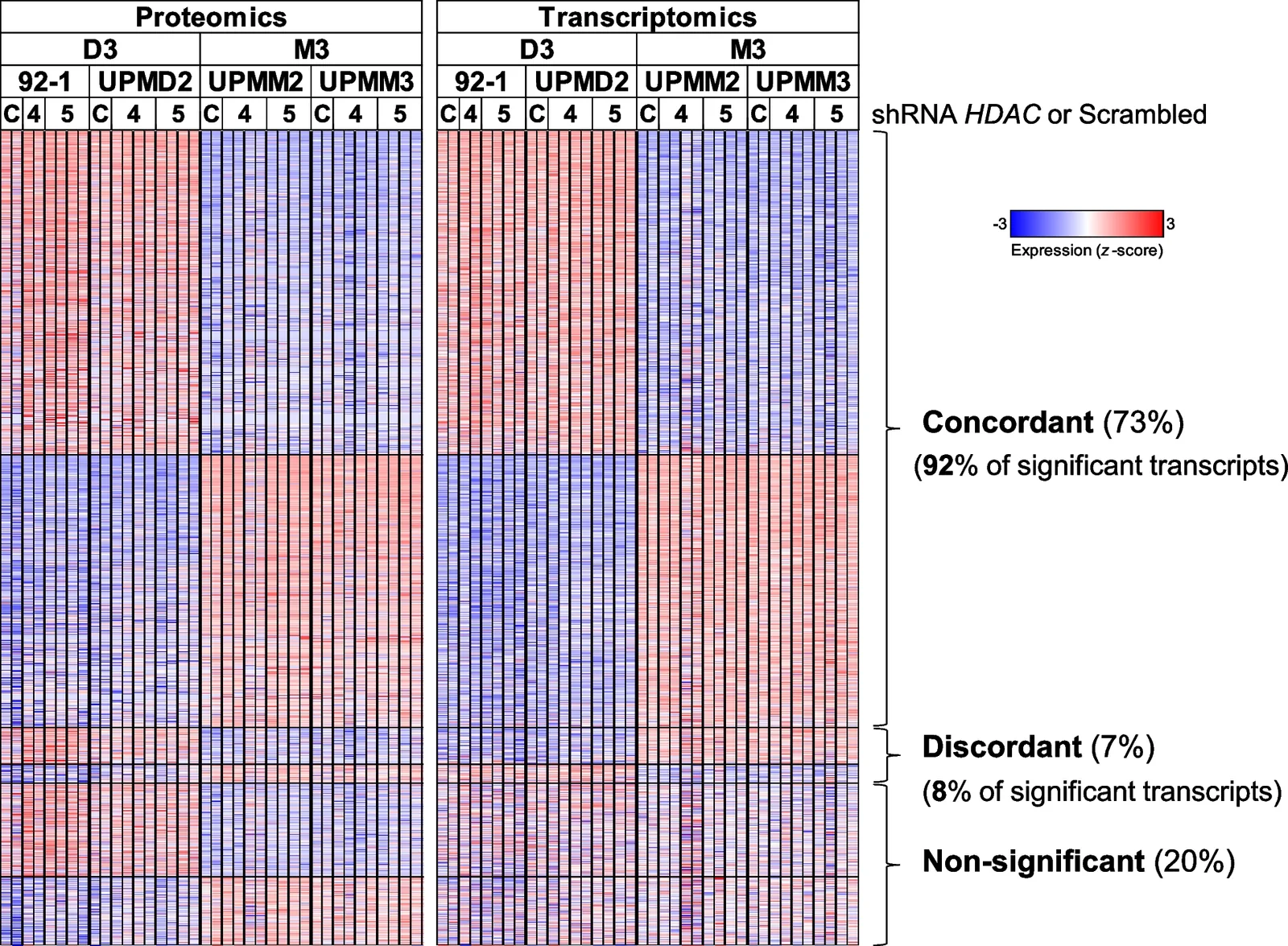
Proteomics and transcriptomics of D3 *vs*. M3 cell lines are largely concordant. After FDR correction, significant proteins that were differentially expressed between the D3 and M3 cell lines at *q* < 0.1 were integrated with their corresponding transcripts. This revealed that 80% of the transcripts were also significant at *q* < 0.1. Notably, 73% of the transcripts (and 92% of the significant transcripts) exhibited concordant fold changes, while only 7% (and 8%, respectively) exhibited discordant changes. C, control shRNA; 4, sh*HDAC4*; 5, sh*HDAC5*

To address the modest changes in gene expression induced by *HDAC4* or *HDAC5* knockdown in the studied UM cell lines, we integrated transcriptomic data with LC–MS-based proteomic analyses. To test the comparability of transcriptomics with proteomics, we compared all samples from D3 cell lines (92–1 and UPMD2) with all samples from M3 cell lines (UPMM2 and UPMM3), regardless of gene knockdown, which shows the strongest differences by principal component analysis (Fig. 5). Consequently, of the 5,796 genes with corresponding protein expression, 1,649 (28%) had a significant false-discovery rate (FDR) corrected *P-*value below 0.1 in both transcriptomics and proteomics when comparing the D3 and M3 cell lines, regardless of knockdown (Table 1). Notably, 92% of these genes/proteins exhibited the same direction of fold change for upregulation/downregulation, which is highly unlikely to occur by chance (Chi-square test, *P* < 0.0001) (Fig. 6, Supplementary File 1). Similar results (91%) were obtained when considering a *P-*value below 0.05 for integrating transcriptomics and proteomics (Supplementary Table S3). Therefore, we used this last approach for comparisons between knockdowns and/or different cell lines.

**Table 1:** Proteogenomic analysis of D3 *vs.* M3 cell lines with an FDR-corrected *P* value below 0.1

		**Transcriptomics**			
**Proteomics**	**FDR *q* < 0.1**	**FC < 1**	**FC > 1**	**NS**	**Total**
	**FC < 1**	**822**	**91**	236	1,149
	**FC > 1**	**46**	**690**	172	908
	**NS**	1,259	1,238	1,242	3,739
	**Total**	2,127	2,019	1,650	5,796

*FDR* False-discovery rate, *FC* Fold change, *NS* Non-significant

### *Knockdown of either* HDAC4 *or* HDAC5 *does not affect differentiation, apoptosis, or necrosis*

**Fig. 7:**
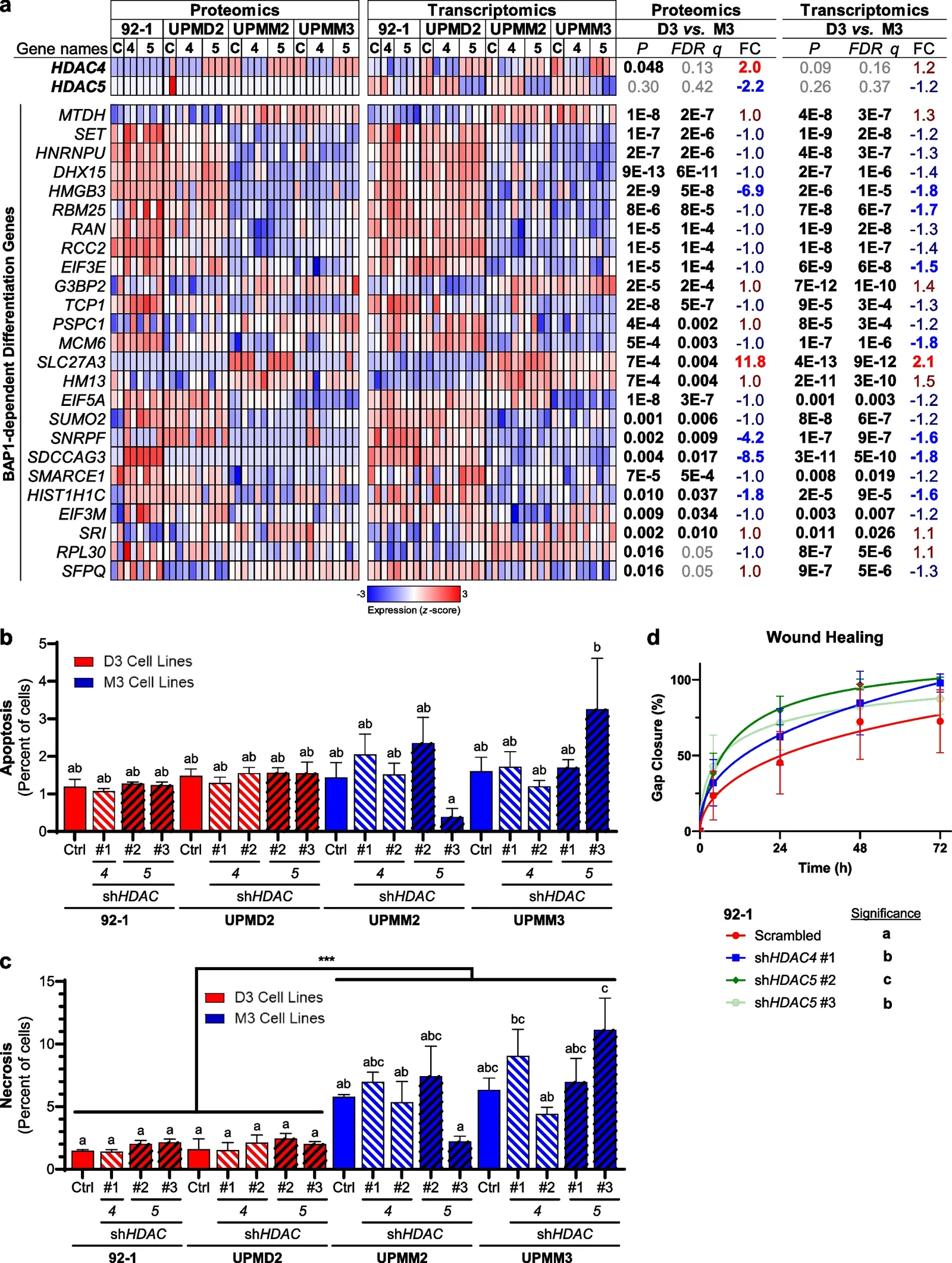
*HDAC4/5* knockdown has no effect on the differentiation, apoptosis, or necrosis of UM cell lines, but it does induce migration in 92–1 cells. (**a**) Proteogenomics analysis of significant BAP1-dependent differentiation genes when comparing D3 *vs*. M3 cells shows that knocking down *HDAC4* or *HDAC5* has a negligible effect, since UM cell lines retain their D3 or M3 characteristics. FDR, false-discovery rate corrected *P* value; FC, fold change. The non-significant BAP1-dependent differentiation genes when comparing D3 *vs*. M3 cells are displayed in Supplementary Figure S16. (**b,c**) The analysis of basal apoptosis (**b**) and necrosis (**c**) levels in UM cell lines via flow cytometry reveals no significant differences resulting from either *HDAC4* or *HDAC5* knockdown, while M3 cell lines display elevated necrosis. Data are presented in percentage of cells positive for annexin V (apoptosis) or 7-AAD (necrosis) for the indicated conditions. Data represent mean ± SE (*n* = 3). ***, *P* < 0.001 by two-factor ANOVA. Non-overlapping letters (e.g., “a” *vs*. “b”) represent significant differences (*P* < 0.05) by one-factor ANOVA and Student–Newman–Keuls post hoc test. (**d**) A wound-healing assay evaluating cell migration in 92–1 cells shows that *HDAC4* and *HDAC5* knockdown improves gap closure over time. Non-overlapping letters (e.g., “a” *vs*. “b”) represent significant differences (*P* < 0.05) by three-factor ANOVA and Student–Newman–Keuls post hoc test

BAP1 promotes lineage commitment and differentiation in developmental systems by maintaining H3K27ac at key promoters, while loss of BAP1 leads to failure of differentiation programs and can be rescued by HDAC inhibition or by *Hdac4* knockdown in *Xenopus* (Kuznetsov et al. 2019). In trophoblast models, reduced BAP1 is required to trigger epithelial-mesenchymal transition (EMT) and invasion, whereas restoring BAP1 preserves epithelial traits and suppresses mesenchymal markers, showing that the BAP1/ASXL axis actively tunes EMT during differentiation (Perez-Garcia et al. 2021). We then evaluated the effects of *HDAC4* and *HDAC5* knockdown on the proteogenomics of BAP1-dependent differentiation genes (Kuznetsov et al. 2019). Although stable knockdown of *HDAC4* and *HDAC5* significantly decreased their protein and gene expression, particularly in M3 cell lines, BAP1-dependent differentiation genes remained unaffected by *HDAC4* or *HDAC5* knockdown, retaining their D3 or M3 characteristics (Fig. 7a). These results are consistent with those of our cell viability and colony formation assays (Fig. 3d-g, Supplementary Figure S12).

To provide additional evidence, we conducted apoptosis and necrosis assays on these cell lines. Under basal (untreated) conditions, the rate of apoptosis remained consistently low, staying strictly below 5%, across all tested cell lines. No significant differences were observed between the cell lines under these conditions (Fig. 7b). Surprisingly, 1 µM staurosporine for 6 h did not trigger massive apoptosis induction, with rates remaining below 10% in all groups (Supplementary Figure S18a). The 92–1 and UPMD2 cell lines exhibited virtually no increase in apoptotic cell death compared to their respective baselines. In contrast, the UPMM2 and UPMM3 lines demonstrated a slight but noticeable trend toward elevated apoptotic rates ranging between approximately 4% and 8%. The highest apoptotic rate in this group was observed in UPMM3 cells with *HDAC5* #1 knockdown.

In contrast to the apoptotic profile, necrotic cell death revealed pronounced, cell line-specific variations (Fig. 7c). The 92–1 and UPMD2 cell lines demonstrated high resistance to necrosis. The percentage of necrotic cells remained consistently low, under 5%, under both basal conditions and following 1 µM staurosporine treatment. Conversely, the UPMM2 and UPMM3 lines displayed markedly elevated baseline levels of necrosis, ranging from 5 to 12% (*P* < 0.001). This effect was heavily exacerbated upon exposure to 1 µM staurosporine (Supplementary Figure S18b). Necrotic rates substantially increased in nearly all UPMM2 and UPMM3 cell lines, reaching peak values of up to 18%, especially in the UPMM3 cells with *HDAC5* knockdown.

Interestingly, the UPMM2 sh*HDAC5* #3 cell line exhibited a significant reduction in necrosis under both basal and staurosporine-stimulated conditions. Necrotic rates dropped to approximately 2–4%, while the other UPMM2 cell lines yielded rates between 7 and 13%. Within the UPMM3 cell lines, sh*HDAC5* #3 induced the highest necrotic rates observed across the entire assay. Under basal conditions, it accounted for approximately 12%, surging to nearly 18% upon staurosporine treatment.

### Knocking down either HDAC4 or HDAC5 improves the wound-healing ability of 92–1 cells

To provide additional evidence on the consequences of *HDAC4* or *HDAC5* knockdown, we conducted wound-healing assays on the 92–1 cell line only, due to its fast-growing characteristics and ability to cover the entire plate compared to the other patient-derived UM cell lines. We observed that the knockdown of *HDAC4* and *HDAC5* significantly (*P* < 0.05) improved the gap closure over time in the wound-healing assay (Fig. 7d, Supplementary Figure S19). Therefore, *HDAC4* and *HDAC5* knockdown enhances the migration and growth of 92–1 cells.

### Global effects of stable HDAC4 and HDAC5 knockdown

To gain further insight into the proteogenomics of *HDAC4* and *HDAC5* knockdown, we analyzed the significant genes and proteins within both platforms using Ingenuity Pathway Analysis (IPA). We focused on canonical pathways significant in more than one cell line or subgroup combination. The global effects of stable *HDAC4* knockdown in UM cells, when comparing the shRNA controls with sh*HDAC4* for all cell lines, were characterized by an overrepresentation of genes/proteins involved in ‘Cell Cycle: G1/S Checkpoint Regulation Signaling’ (Supplementary Table S4, Supplementary Figure S20) and ‘Cyclins and Cell Cycle Regulation’ pathways (Supplementary Figure S21). The specific effects of *HDAC4* knockdown in D3 cell lines revealed an overrepresentation of genes/proteins involved in ‘Xenobiotic Metabolism Signaling’ and ‘ERK/MAPK Signaling’, whereas the stable knockdown of *HDAC5* in D3 cell lines resulted in the overrepresentation of the ‘Melanocyte Development and Pigmentation Signaling’ pathway in both 92–1 and UPMD2 cell lines (Supplementary Table S4). On the other hand, the global effects of the stable knockdown of *HDAC4* in M3 cell lines showed an overrepresentation of genes/proteins involved in ‘Adipogenesis Pathway’ in both UPMM2 and UPMM3 cell lines, as well as the ‘Mitochondrial Dysfunction’ (Supplementary Table S4). The knockdown of *HDAC5* in M3 cell lines showed an overrepresentation of ‘Agrin Interactions at Neuromuscular Junction’ and ‘CDK5 Signaling’ in both UPMM2 and UPMM3 cell lines (Supplementary Table S4).

Cyclin-dependent kinases (CDKs) regulate cell cycle progression and are inhibited by CDK inhibitors (CDKIs) (Reed et al. 1994; Sherr and Roberts 1999). Constitutive activation of CDKs is an oncogenic event, leading to unrestricted passing of cell cycle checkpoints and uncontrolled proliferation (Malumbres and Barbacid 2009). Several CDKIs, including cyclin-dependent kinase inhibitor 1B (*CDKN1B*)*,* are downregulated in primary and metastatic UM compared to normal uveal melanocytes due to repression by the transcription factor *zinc finger E-box binding homeobox 1* (*ZEB1)* (Chen et al. 2017). Of note, we included three CDKi in our initial drug screening panel (LDC000526:15, LDC208447:01, and Palbociclib), but we did not detect remarkable sensitivities to these compounds in our cell lines (Fig. 1). This suggests that HDACi or knockdown of *HDAC4/HDAC5* might sensitize UM cells to CDKi. To test this hypothesis, we performed a combination screening of panobinostat and palbociclib on 92–1 cells. Knocking down *HDAC4* slightly increased the synergism of 92–1 cells to the combination of panobinostat and palbociclib (Supplementary Figure S23). However, knockdown of *HDAC5* #3, but not #2, showed strong synergism with this drug combination.

**Fig. 8:**
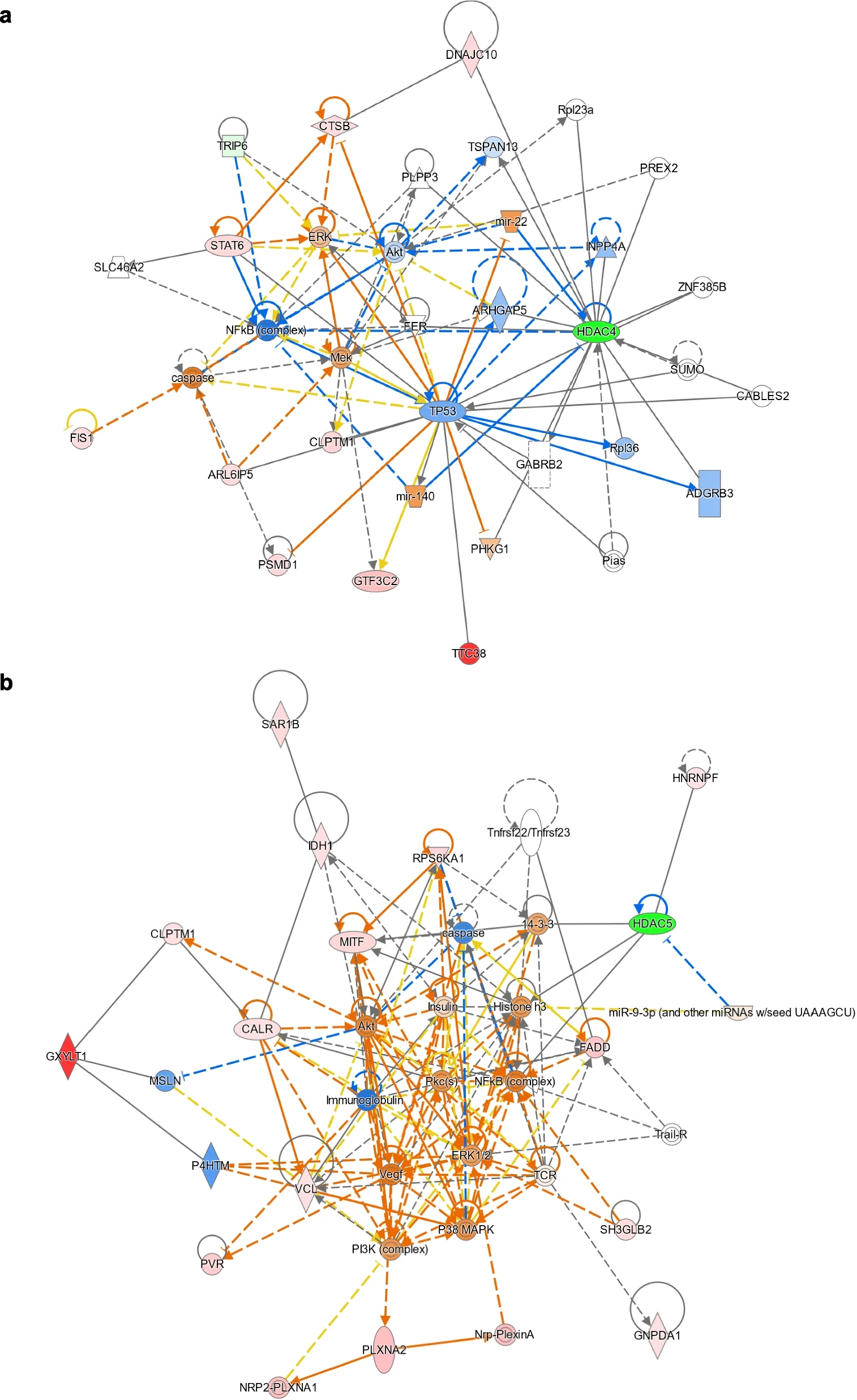
HDAC4 and HDAC5 might regulate NF-κB complex. (**a**) Network analysis of UM cell lines with *HDAC4* knockdown reveals an indirect interaction between HDAC4 and NF-κB and Rpl36, respectively. (**b**) Network analysis of UM cell lines with *HDAC5* knockdown reveals a negative interaction between HDAC5 and NF-κB. Ingenuity Pathway Analysis shows downregulated genes in blue/green (predicted/measured) and upregulated genes in orange/red (predicted/measured)

We could identify an HDAC4-related network upon *HDAC4* knockdown, revealing nuclear factor kappa-light-chain-enhancer of activated B cells (NF-κB) and Rpl36 as potential positive indirect downstream targets of HDAC4 (Fig. 8a). It has been shown that class II HDACi, as well as knockdown of *HDAC4*, reduce phosphorylation of NF-κB and prevent partial epithelial-to-mesenchymal-transition in kidney tissue, a cellular program that is also important in cancer progression and metastasis (Xiong et al. 2019; Dongre and Weinberg 2019). At the same time, another study investigated bromodomain and extraterminal domain family (BET) inhibitor (BETi) resistance in UM and found increased expression of NF-κB signaling genes in resistant cells, as well as synergistic effects of NF-κB inhibition with BETi (Ambrosini et al. 2019). We tested the BETi JQ1 in our initial drug screening panel as well as in the epigenetic library screen but detected incomplete responses in most of our UM cell lines (Fig. 1, Supplementary Figure S3). However, treatment with the dual BET/HDACi TW9 resulted in a complete response in all cell lines, but only at very high concentrations (Fig. 1). Conversely, compared to *HDAC4* knockdown, *HDAC5* knockdown displayed a negative interaction of HDAC5 with NF-κB, as well as with ERK/MAPK (Fig. 8b).

### NF-κB p65 phosphorylation is decreased in UM cell lines with BAP1 loss and M3

**Fig. 9:**
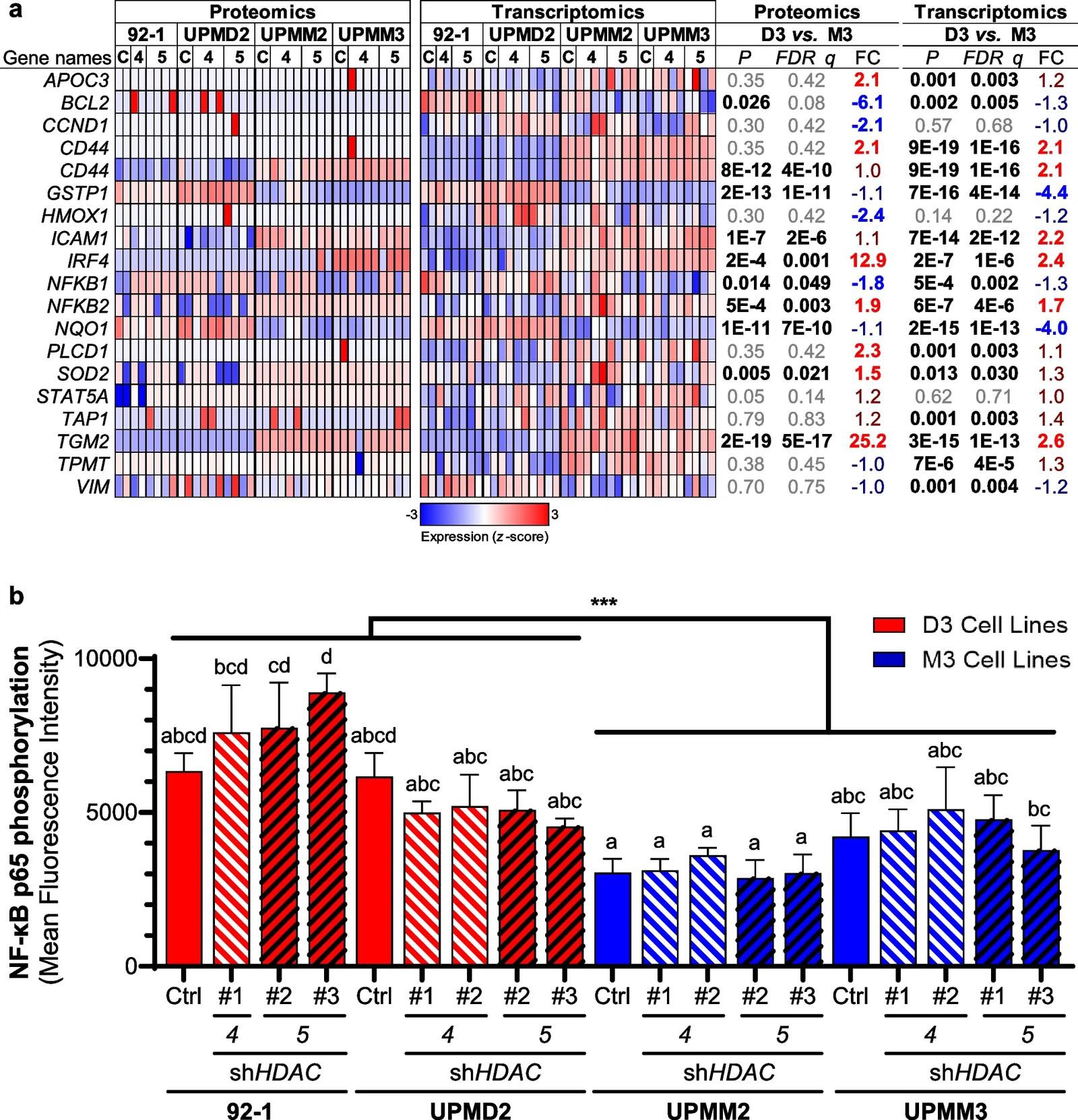
NF-κB p65 is less phosphorylated in M3 cells with BAP1 loss. (**a**) Proteogenomic analysis of NF-κB target genes shows stronger D3 and M3 characteristics than the effect of *HDAC4* or *HDAC5* knockdown. FDR, false-discovery rate corrected *P* value; FC, fold change. (**b**) Phospho-flow cytometry was used to quantify the phosphorylation of NF-κB p65 in UM cell lines and showed no significant differences resulting from *HDAC4* or *HDAC5* knockdown. In contrast, M3 cell lines displayed lower NF-κB activity. Data are presented as mean fluorescence intensity (MFI) of phospho-NF-κB p65 for the indicated conditions. Data represent mean ± SE (*n* = 5). ***, *P* < 0.001 by two-factor ANOVA. Non-overlapping letters (e.g., “a” *vs*. “b”) represent significant differences (*P* < 0.05) by one-factor ANOVA and Student–Newman–Keuls post hoc test

To evaluate the involvement of the NF-κB pathway in UM pathogenesis, we examined the proteogenomics of NF-κB target genes (Fig. 9a) and quantified the phosphorylation of NF-κB p65 by phospho-flow cytometry across all tested UM cell lines and experimental conditions (Fig. 9b). While there were no substantial disparities caused by the knockdown of *HDAC4* or *HDAC5*, UM cell lines with D3 exhibited differentially expressed NF-κB target genes and proteins compared to M3 cell lines with BAP1 loss (Fig. 9a). Similarly, a two-factor ANOVA revealed no significant differences in the phosphorylation of NF-κB p65 caused by *HDAC4* or *HDAC5* knockdown (Fig. 9b). However, UM cell lines with *BAP1* mutations and the M3 genotype exhibited significantly lower NF-κB p65 activity than UM cell lines with the D3 genotype (Fig. 9b). These findings are consistent with the inactivation of the NF-κB signaling pathway by *BAP1* mutations in UM cells (Zhang and Wu 2023).

## Discussion

Uveal melanoma (UM) is a rare but highly aggressive malignancy that is characterized by pronounced therapeutic resistance, particularly in the metastatic setting (Singh and Borden 2005). To identify potential vulnerabilities, we systematically assessed the effects of a broad panel of pharmacologically active compounds on cell viability in primary-derived UM cell lines. Among all tested agents, the pan-histone deacetylase inhibitor (HDACi) panobinostat consistently reduced viability across all cell lines, including those harboring monosomy 3 (M3) and *BAP1* mutations. In contrast, the class I/IV HDACi mocetinostat was substantially less effective, prompting us to investigate whether selective targeting of individual class II HDACs could account for the differential responses.

HDAC inhibitors have demonstrated clinical efficacy, particularly in hematological malignancies (Marks 2007; Grant et al. 2010; Laubach et al. 2015), and preclinical studies suggest potential activity in UM (Moschos et al. 2018). However, currently approved pan-HDAC inhibitors show limited efficacy in solid tumors and are associated with significant toxicity, leaving a narrow therapeutic window (Hontecillas-Prieto et al. 2020; Gryder et al. 2012). Panobinostat, in particular, exhibits pronounced off-target effects (Becher et al. 2016). These limitations provide a strong rationale for exploring isoform-selective HDAC targeting strategies to improve therapeutic index.

We focused on HDAC4 and HDAC5 based on prior reports linking *HDAC4* expression to BAP1 loss and increased sensitivity of BAP1-deficient UM cells to pan-HDAC inhibition (Kuznetsov et al. 2019; Kuznetsoff et al. 2021). BAP1 functions as an epigenetic regulator involved in transcriptional control and cellular differentiation, and its loss promotes a dedifferentiated, aggressive UM phenotype (Carbone et al. 2013; Matatall et al. 2013). Consistent with this, HDAC inhibition has been shown to induce differentiation-associated gene expression programs and sensitize BAP1-deficient cells in UM and other cancer entities (Landreville et al. 2012; Rosato et al. 2003; Wang et al. 1999; Göttlicher et al. 2001; Sacco et al. 2015).

Despite these observations, our data do not support a simple BAP1-dependent vulnerability to HDAC4 or HDAC5 inhibition. Although panobinostat potently reduced UM cell viability, neither BAP1 status nor M3 consistently predicted sensitivity. Moreover, selective pharmacological inhibition or genetic knockdown of *HDAC4* and/or *HDAC5* failed to recapitulate the effects of pan-HDAC inhibition. Although *Hdac4* knockdown rescued the Bap1-deficient phenotype in *Xenopus laevis* and significantly impaired UM cell proliferation in a BAP1-dependent manner (Kuznetsov et al. 2019), we observed minimal differences in terms of cell viability, colony formation, transcriptional and proteomic profiles, expression of BAP1-dependent differentiation genes, and apoptosis and necrosis analyses by *HDAC4* or *HDAC5* knockdown in our patient-derived UM cell lines. Perhaps HDACs have higher functional redundancy in humans, requiring additional knockdowns to observe phenotypic changes.

These findings likely reflect the complexity of HDAC-dependent regulatory networks and the limited specificity of currently available “selective” HDAC inhibitors, which frequently target additional HDAC isoforms or unrelated proteins. For example, tasquinimod demonstrated a high binding affinity not only for HDAC4 (*K*_d_ 10–30 nM) but also for the calcium-binding protein S100A9 (Isaacs et al. 2013). Tasquinimod is also an aryl hydrocarbon receptor (AHR) agonist that binds with an EC_50_ of 1 μM, producing unwanted off-target side effects (Isaacs et al. 2023). Therefore, studies using μM concentrations of tasquinimod can generate transcriptional changes unrelated to HDAC4, which complicates pathway interpretation. On the other hand, LMK-235 is a highly selective inhibitor of HDAC4 and HDAC5 with IC_50_ values of 11.9 nM and 4.22 nM, respectively, but also exhibits weaker activity against HDAC6, HDAC1, HDAC11, HDAC2, and HDAC8, with IC50 values of 55.7 nM, 320 nM, 852 nM, 881 nM, and 1278 nM, respectively (Marek et al. 2013). LMK-235 should be considered a preferential HDAC4/5 inhibitor rather than a selective one because the published IC_50_ panel shows measurable activity at several other HDACs. These off-target activities potentially obscure isoform-specific effects and contribute to cell line-dependent responses. Such off-target activities may also explain the discrepancy between pharmacological inhibition and genetic knockdown experiments observed in this study.

The lack of an effect of tasquinimod on the viability of all the UM cell lines studied cannot be explained by insufficient HDAC4 engagement alone, since the drug has a documented low nanomolar binding affinity for HDAC4, as well as target-pathway phenocopy and additional biology beyond HDAC4. A more likely explanation is that, although HDAC4 engagement is necessary, it is not sufficient. The observed effect depends on tumor context, microenvironment stress, and confounding off-target actions.

In summary, while pan-HDAC inhibition robustly compromises UM cell viability, selective targeting of HDAC4 or HDAC5 alone is insufficient to reproduce this effect. Our results suggest that effective HDAC-based therapeutic strategies in UM may require coordinated inhibition of multiple HDACs or alternative approaches that exploit broader epigenetic vulnerabilities rather than single-isoform dependencies.

To further dissect the molecular consequences of HDAC4 and HDAC5 perturbation beyond effects on cell viability, we integrated transcriptomic and proteomic analyses across UM cell lines with different chromosomal and *BAP1* mutation status. While stable knockdown of *HDAC4* or *HDAC5* induced only modest transcriptional changes, integration with LC–MS–based proteomics revealed robust and highly concordant alterations at the protein level. Notably, a substantial fraction of significantly regulated genes and proteins displayed consistent directionality across platforms, underscoring the biological relevance of the observed changes and validating the integrative approach.

Pathway-level analyses indicated that HDAC4 and HDAC5 contribute to distinct, context-dependent regulatory programs in UM. *HDAC4* knockdown predominantly affected cell cycle–related pathways, including G1/S checkpoint regulation and cyclin signaling, whereas *HDAC5* knockdown was associated with pathways linked to differentiation and lineage identity. Importantly, these effects differed between D3 and M3 cell lines, suggesting that chromosomal status and BAP1 loss shape HDAC-dependent signaling networks rather than defining a uniform dependency on individual HDAC isoforms.

Network analyses further implicated NF-κB and ERK/MAPK signaling as downstream nodes differentially linked to HDAC4 and HDAC5, supporting a model in which class IIa HDACs modulate broader transcriptional and stress-response programs rather than acting as dominant drivers of UM cell survival. Proteogenomics of NF-κB target genes and functional validation of NF-κB activity was not able to detect differences in NF-κB target genes and NF-κB p65 phosphorylation caused by stable *HDAC4* or *HDAC5* knockdown. These findings are consistent with the limited impact of selective HDAC4/5 inhibition on cell viability, colony formation, BAP1-dependent differentiation genes, apoptosis, and necrosis, suggesting that class IIa HDACs may primarily fine-tune oncogenic and inflammatory signaling rather than serve as standalone therapeutic targets. However, we observed a significant decrease in NF-κB p65 phosphorylation in UM cell lines with M3 and *BAP1* mutations. It has been demonstrated that *BAP1* mutations inhibit NF-κB pathway, reducing cytokine secretion and antigen presentation, fostering immunosuppression (Zhang and Wu 2023). Despite successful results in cutaneous melanoma, monotherapies with immune checkpoint inhibitors have shown little clinical benefit in patients with metastatic UM (Wang et al. 2024). HDAC inhibitors can interact with these strategies in two opposite ways. One way is by increasing HLA class I expression and the antigen-presentation machinery (Souri et al. 2020), making tumor cells more visible to T cells and potentially improving immunotherapy effectiveness. Conversely, HDAC inhibitors can remodel inflammatory transcription programs, including NF-κB-linked outputs, so their overall impact hinges on dosage, HDAC selectivity, and tumor context (Kroesen et al. 2014).

Collectively, our multi-omics analysis highlights that HDAC4 and HDAC5 exert measurable and biologically meaningful effects on UM cell state, yet these effects are insufficient to phenocopy pan-HDAC inhibition. This supports the concept that the therapeutic activity of pan-HDAC inhibitors arises from simultaneous interference with multiple epigenetic and signaling axes, emphasizing the need for combinatorial or systems-level strategies, such as panobinostat and palbociclib, when targeting epigenetic regulators in uveal melanoma.

## Supplementary Information

Supplementary Material 1.

Supplementary Material 2.

## Data Availability

All data supporting the findings of this study are available within the paper and its Supplementary Information. The gene expression data generated in this study have been deposited at the Gene Expression Omnibus (GEO) repository with accession number GSE199470 (https://www.ncbi.nlm.nih.gov/geo/query/acc.cgi?acc=GSE199470). The proteomics data from this study have been deposited at the PRoteomics IDEntifications (PRIDE) Archive database ( https://www.ebi.ac.uk/pride).

## Abbreviations

BAP1: BRCA1-associated protein 1
BET: Bromodomain and extraterminal domain family
BETi: Bromodomain and extraterminal domain family inhibitor
CDK: Cyclin-depdendent kinase
CDKI/CDKi: Cyclin-dependent kinase inhibitor
CDKN1B: Cyclin-dependent kinase inhibitor 1B
D3: Disomy of chromosome 3
HDACi: Histone deacetylase inhibitor
HDACs: Histone deacetylases
IC_50_: Half maximal inhibitory concentration
IPA: Ingenuity Pathway Analysis
M3: Monosomy of chromosome 3
NF-κB: Nuclear factor kappa light chain enhancer of activated B cells
shRNA: Short-hairpin RNA
TCGA: The Cancer Genome Atlas
TCGA-PANCAN: The Cancer Genome Atlas Pan-Cancer
UM: Uveal melanoma
UVM-TCGA: The Cancer Genome Atlas Uveal Melanoma
