# Supplementary material for: Proteogenomic Analysis of HDAC4 and HDAC5 in Uveal Melanoma
Source: Mol Med. 2026 Sep 22;32:156. doi: 10.1186/s10020-026-01652-9 (PMC13595792; doi:10.1186/s10020-026-01652-9)
Supplement: Supplementary file 1 [file 10020_2026_1652_MOESM1_ESM.pdf]

Proteogenomic Analysis of HDAC4 and HDAC5 in Uveal Melanoma

Svenja Henning[1,2], Tanja J. Kutzner[3], Martin Schneider[4], Teresa González-Gimeno[1,5,6], Yue Zheng[1,5], Utta Berchner-Pfannschmidt[5], Dominic Helm[4], Bernd Giebel[3], Nikolaos E. Bechrakis[5] and Samuel Peña-Llopis[1,2,5,*]

[1]Translational Genomics, German Cancer Consortium (DKTK) and Department of Ophthalmology, University Hospital Essen, D-45147 Essen, Germany.

[2]German Cancer Consortium (DKTK) and German Cancer Research Center (DKFZ), Heidelberg, Germany.

[3]Institute for Transfusion Medicine, University Hospital Essen, University of Duisburg-Essen, Essen, Germany.

[4]Proteomics Core Facility, German Cancer Research Center (DKFZ), Heidelberg, Germany.

[5]Department of Ophthalmology, University Hospital Essen, Essen, Germany.

[6]Faculty of Experimental Sciences, Universidad Francisco de Vitoria (UFV), Madrid, Spain.

*Corresponding author: Samuel.Pena-Llopis@uk-essen.de

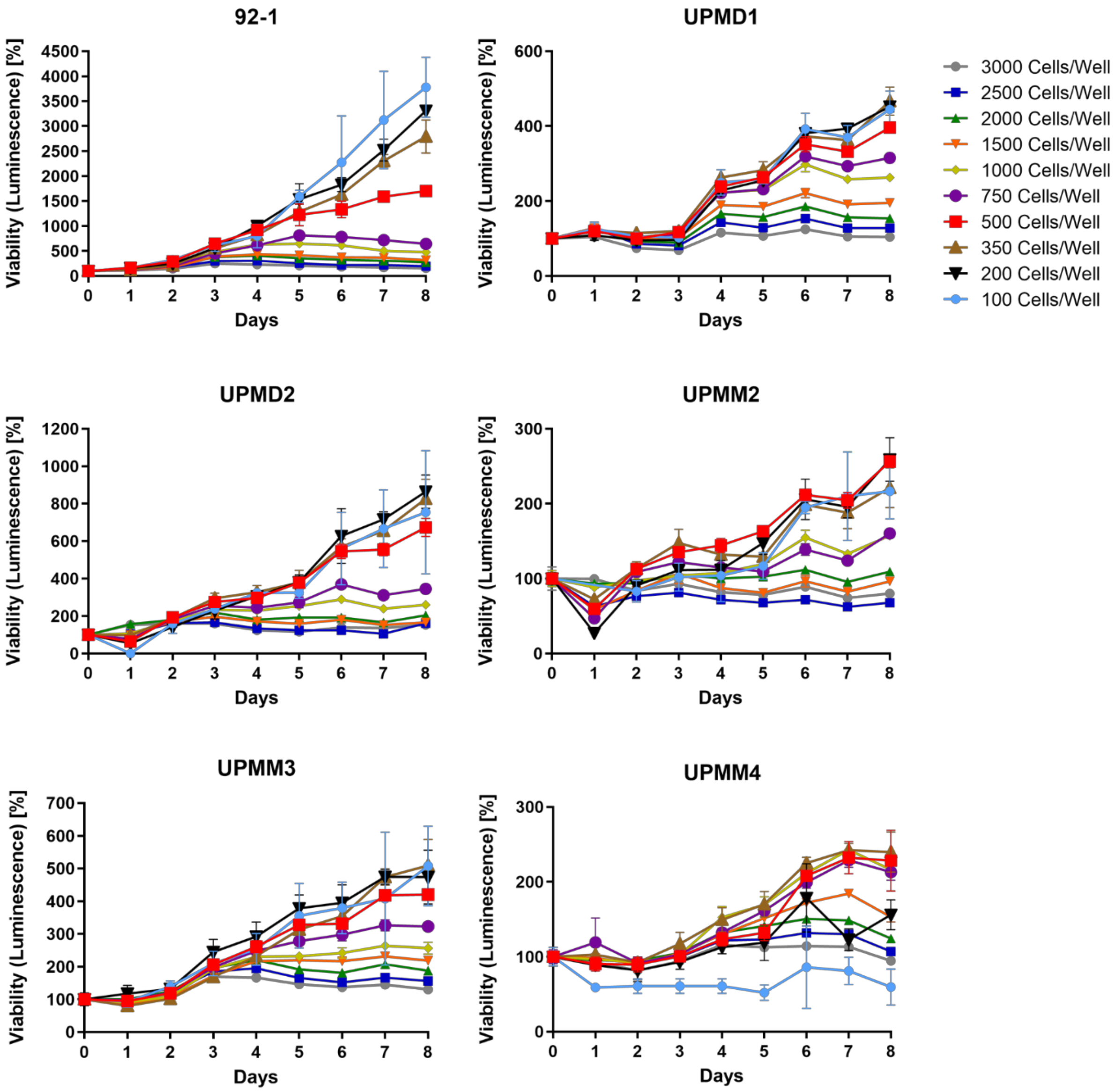

Figure S1. Results of cell number and assay period optimization for drug screening experiments. Proliferation curves of UM cell lines seeded at different cell numbers. Luminescence is normalized to day 0 and expressed as percentage of day 0. A cell number of 500 cells per well and an assay period of 5 days was used for subsequent experiments. Summarized results of one experiment are shown. Data are average ± SD.

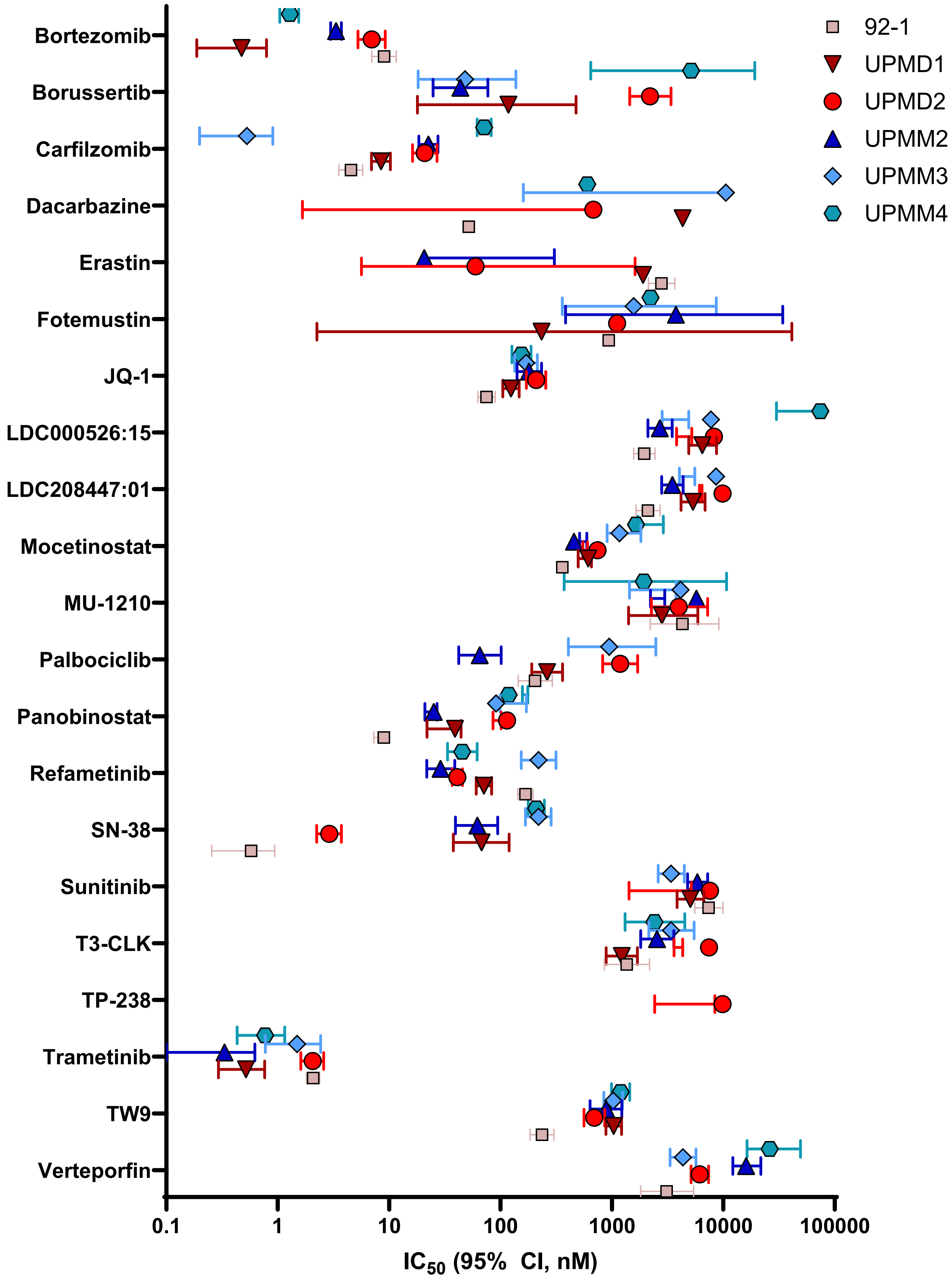

Figure S2. Half maximal inhibitory concentrations (IC_{50}) and 95% Confidence Interval (CI) comparison for UM cell lines. IC_{50}s and 95% CI were calculated from the cell viability assays of Figure 1 and from the cell viability assays of trametinib, refametinib, carfilzomib, and sunitinib previously published in Mergener *et al. Int. J. Mol. Sci.* 2021[1].

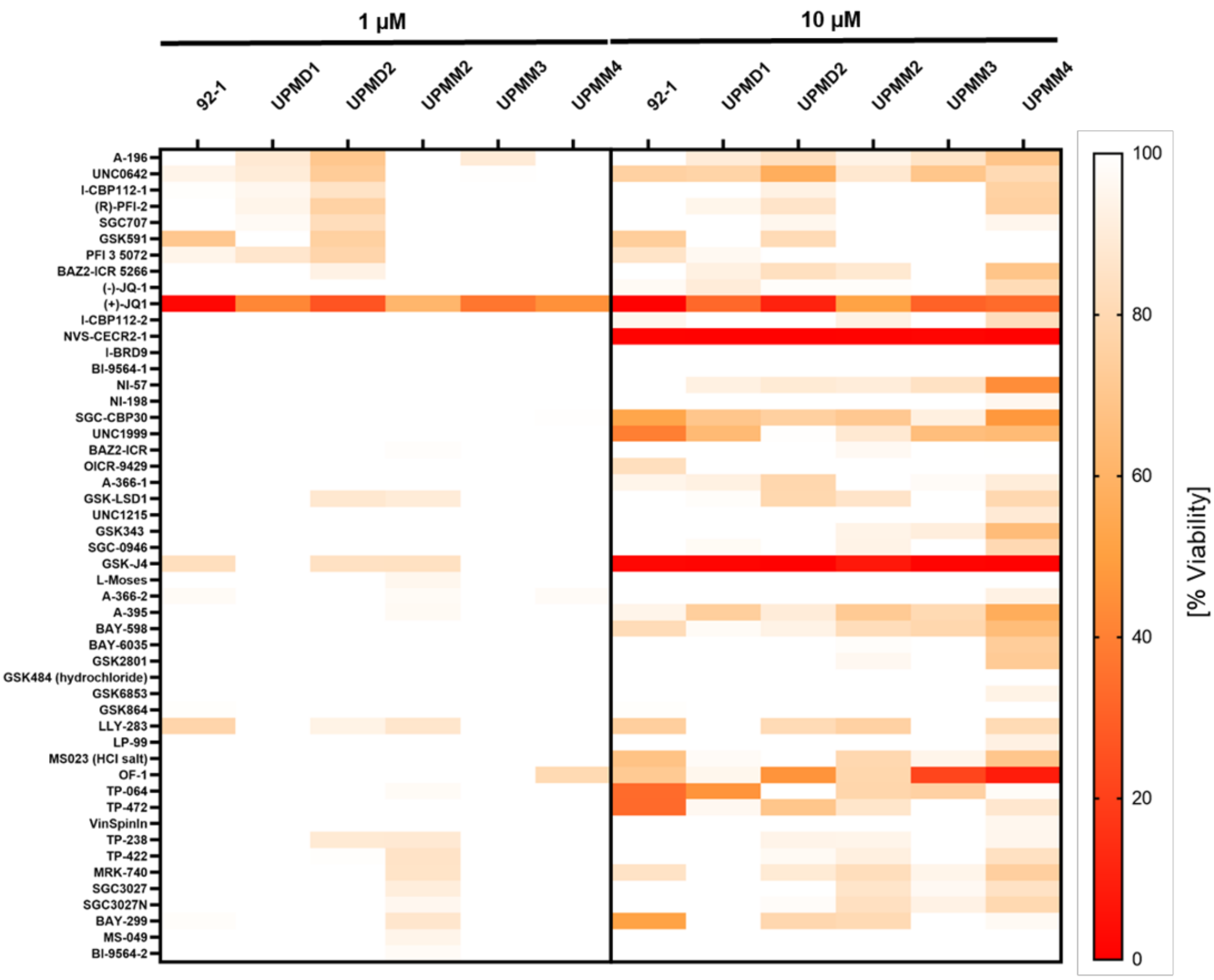

Figure S3. Results of epigenetic library screening in UM cell lines. Cell viability of UM cell lines after treatment with an epigenetic library (https://www.thesgc.org/chemical-probes) consisting of 50 compounds for 5 days at 1 µM or 10 µM. Cell viability is expressed as normalized values in percentage to DMSO controls. Summarized results of one experiment (UPMD2, UPMM4) or two independent experiments (92-1, UPMD1, UPMM2, UPMM3) are shown.

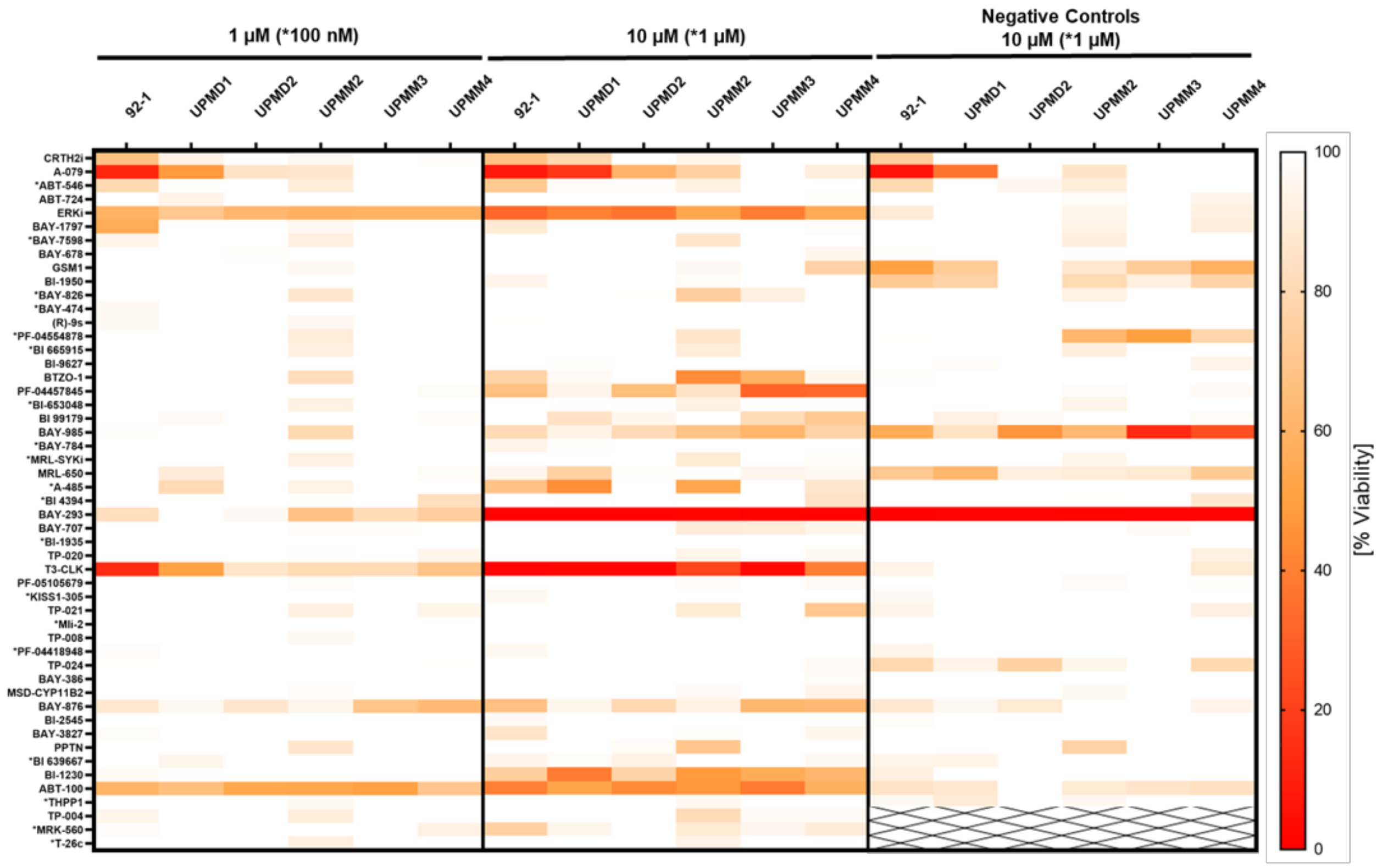

Figure S4. Results of DCP library screening in UM cell lines. Cell viability of UM cell lines after treatment with a DCP library (https://www.sgc-ffm.uni-frankfurt.de) consisting of 51 compounds and 48 respective negative controls for 5 days at 1 µM or 10 µM. Cell viability is expressed as normalized values in percentage to DMSO controls. Summarized results of one experiment (UPMD2, UPMM4) or two independent experiments (92-1, UPMD1, UPMM2, UPMM3) are shown. No negative control compounds were available for TP-004. MRK-560 and T-25c. *, lower concentrations of 100 nM or 1 µM were used due to less concentrated stock dilutions.

A

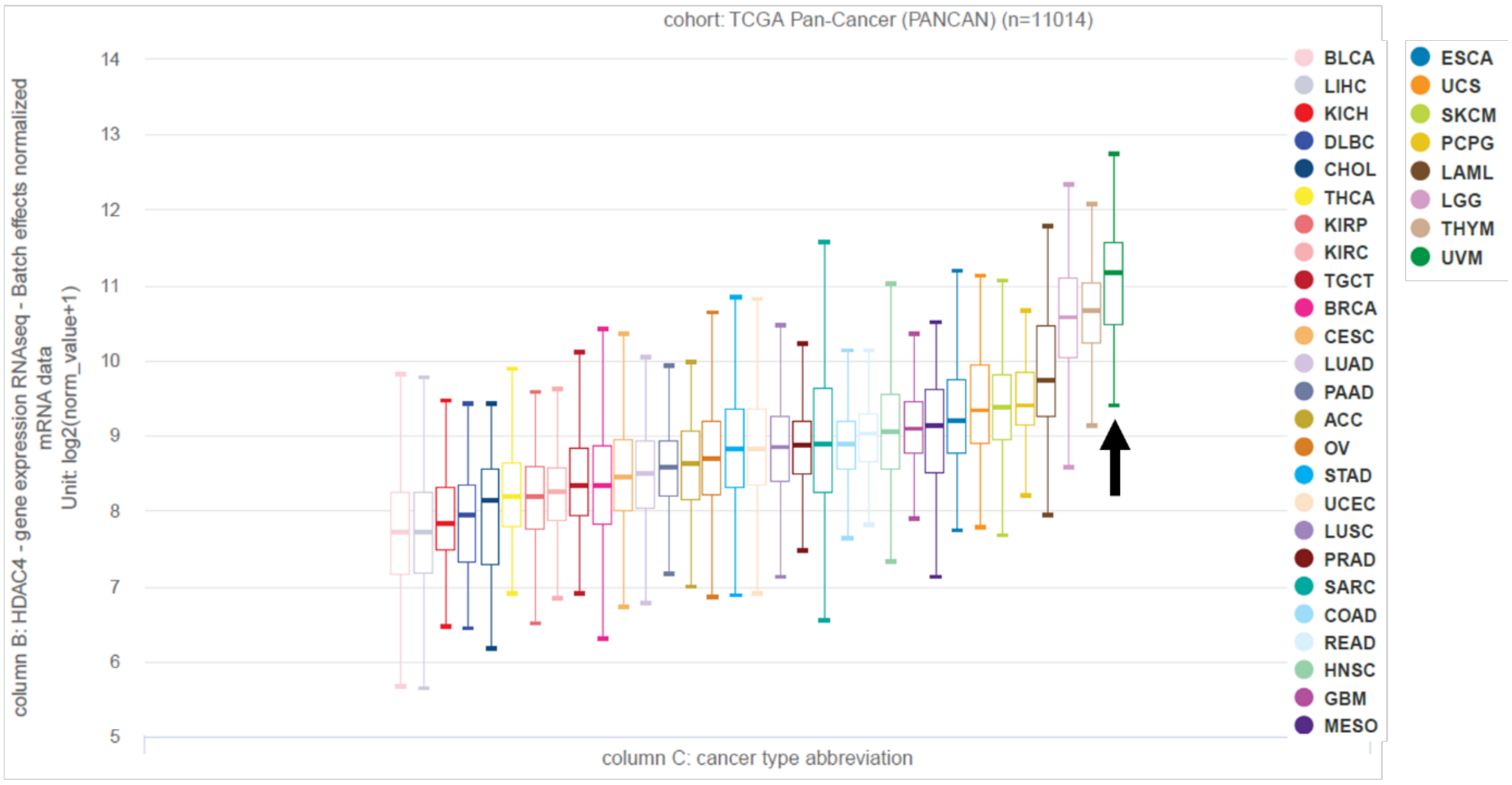

B

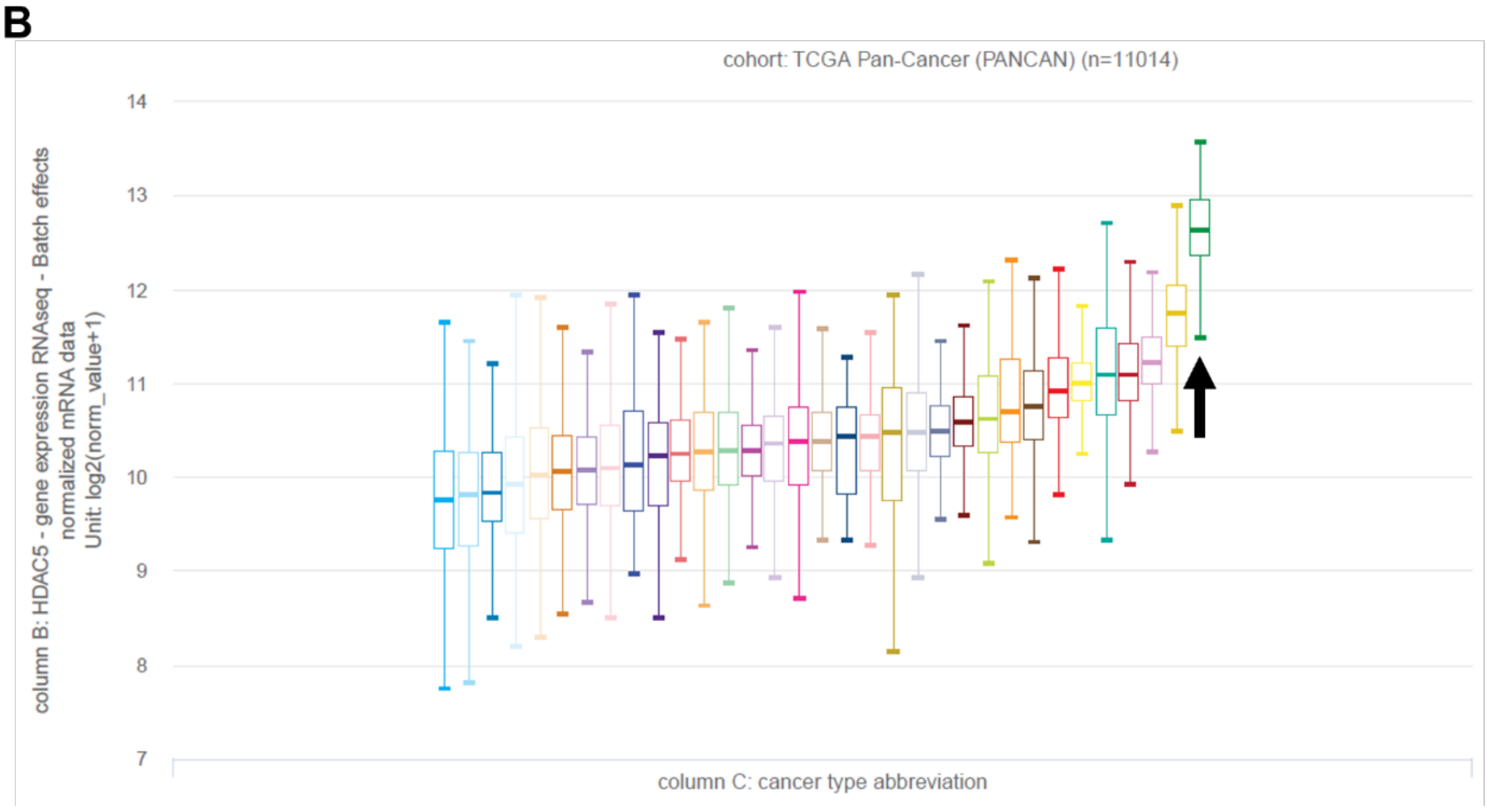

Figure S5. *HDAC4* and *HDAC5* are highly expressed in UM tumors. Gene expression ranking of *HDAC4* (**A**) and *HDAC5* (**B**) in the 33 most prevalent forms of cancer as defined by the Pan-Cancer atlas from TCGA. Arrows: uveal melanoma (UVM). Data and graphs are retrieved from TCGA and UCSC Xena (https://xena.ucsc.edu).

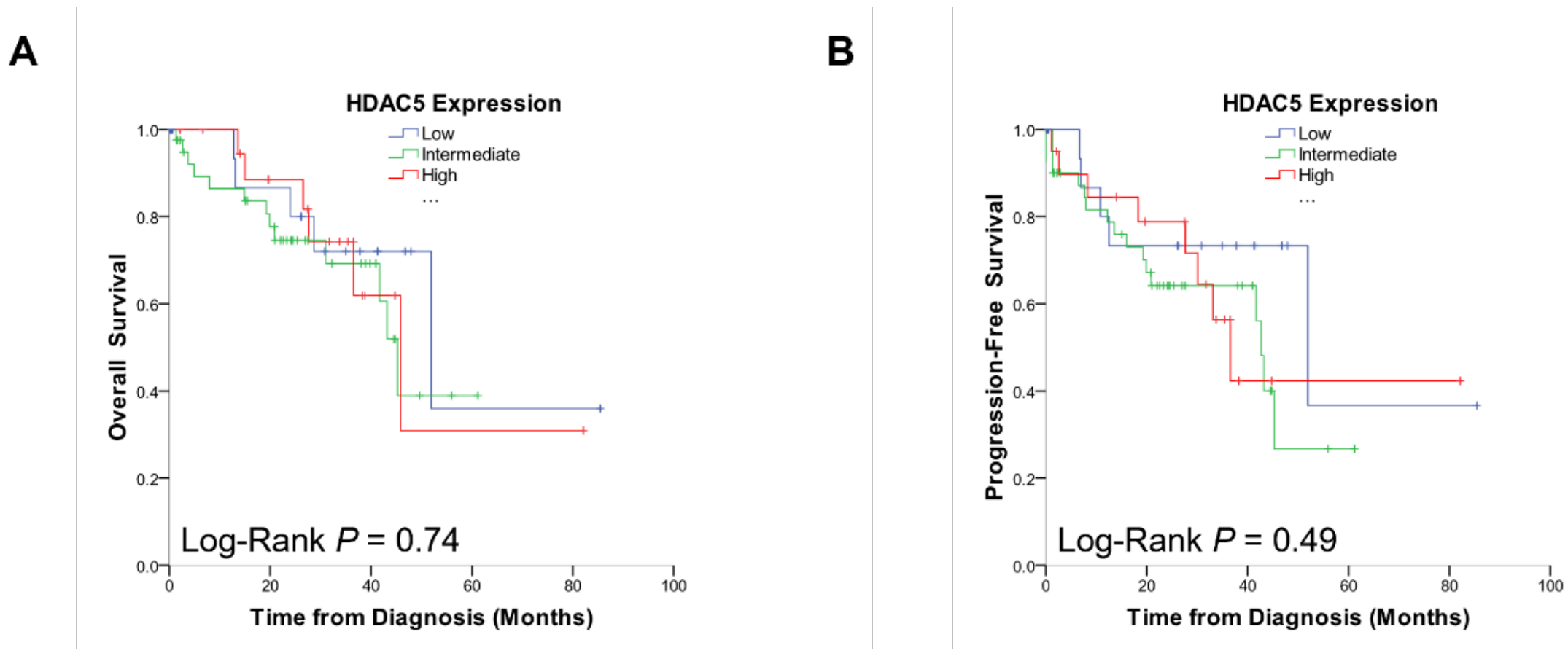

Figure S6. Expression of *HDAC5* does not correlate with survival in UM. Expression of *HDAC5* does not correlate with overall (**A**) and progression-free survival (**B**) in UVM-TCGA. Tumors (*n*=80) were subdivided in three groups of high, intermediate or low gene expression.

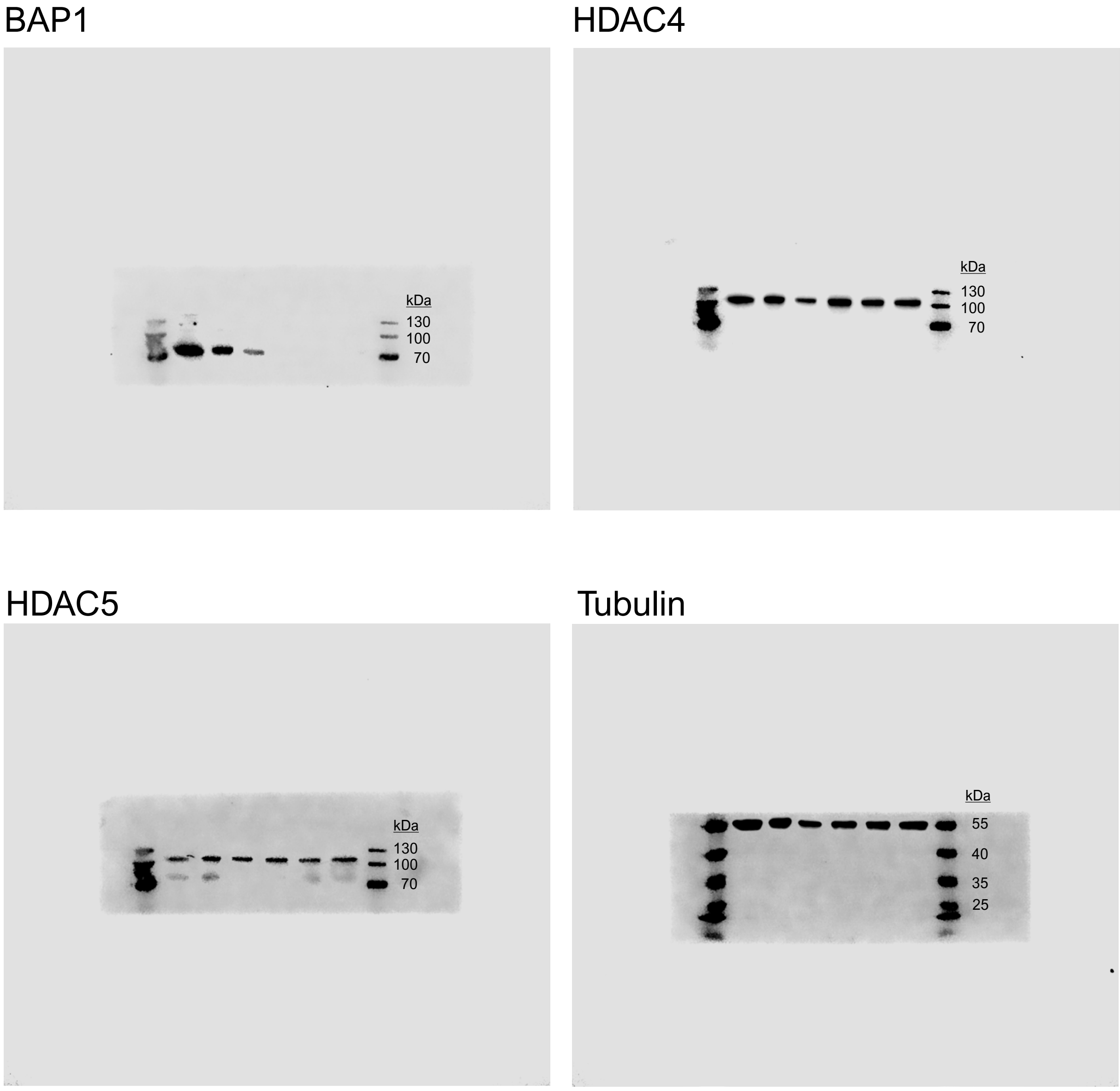

Figure S7. Un-cropped western blots. Original pictures of the western blots shown in Figure 2.

(Next page) Figure S8. pan-HDAC inhibition and MEK inhibition show no strong synergistic effects in uveal melanoma cell lines. Cell viability of UM cell lines after treatment with combinations of pan-HDAC inhibitor panobinostat and MEK inhibitor trametinib for 5 days (**A, C, E, G, I, K**) and respective synergy scores according to the Loewe model (**B, D, F, H, J, L**). Cell viability is expressed as normalized values in percentage to DMSO controls. Summarized results of three independent experiments are shown. Synergy scores and graphs are generated with Combenefit[2].

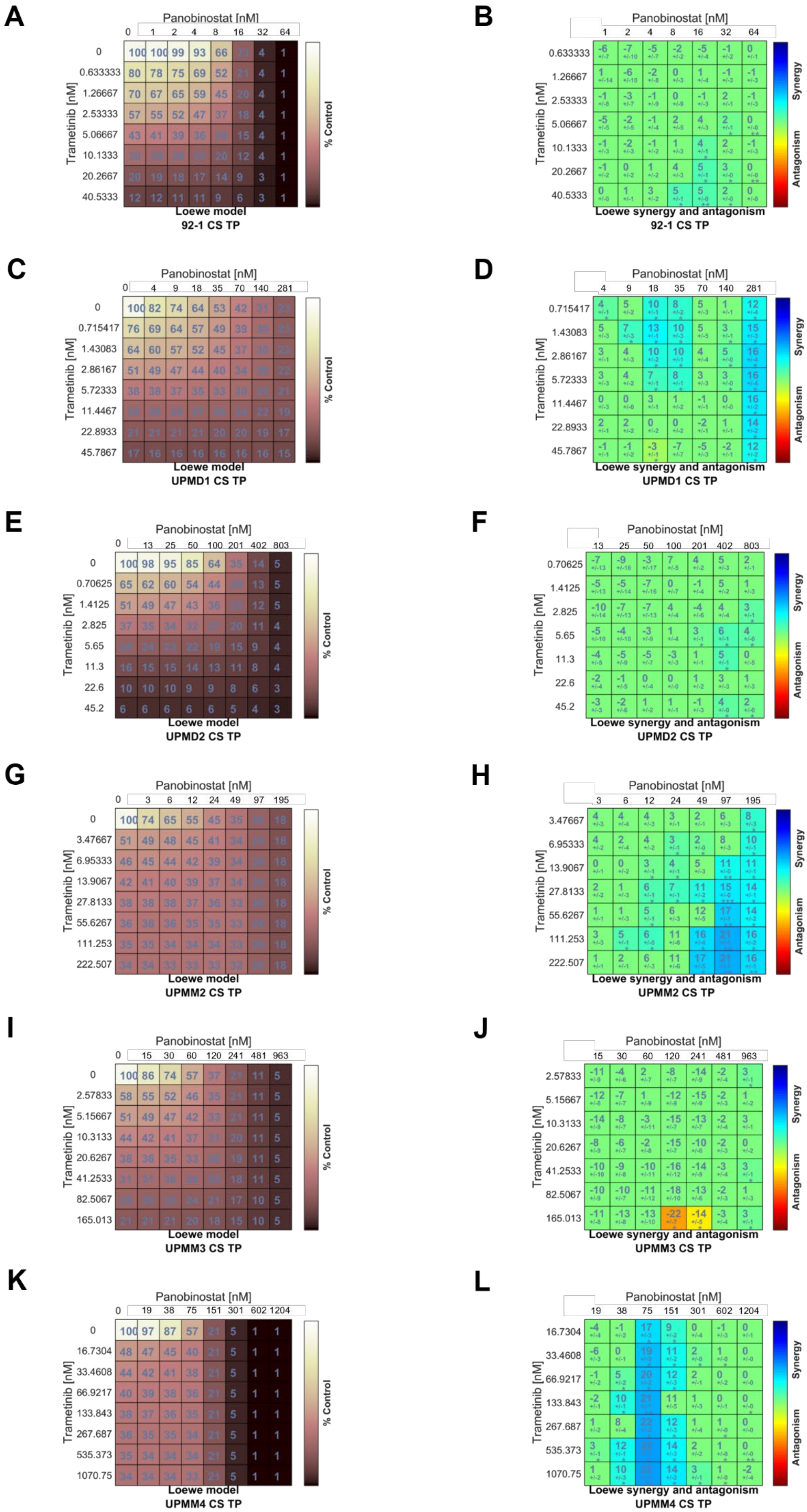

A
Panobinostat [nM]
Trametinib [nM]
% Control
Loewe model
92-1 CS TP
B
Panobinostat [nM]
Trametinib [nM]
Synergy
Antagonism
Loewe synergy and antagonism
92-1 CS TP
C
Loewe model
UPMD1 CS TP
D
Loewe synergy and antagonism
UPMD1 CS TP
E
Loewe model
UPMD2 CS TP
F
Loewe synergy and antagonism
UPMD2 CS TP
G
Loewe model
UPMM2 CS TP
H
Loewe synergy and antagonism
UPMM2 CS TP
I
Loewe model
UPMM3 CS TP
J
Loewe synergy and antagonism
UPMM3 CS TP
K
Loewe model
UPMM4 CS TP
L
Loewe synergy and antagonism
UPMM4 CS TP

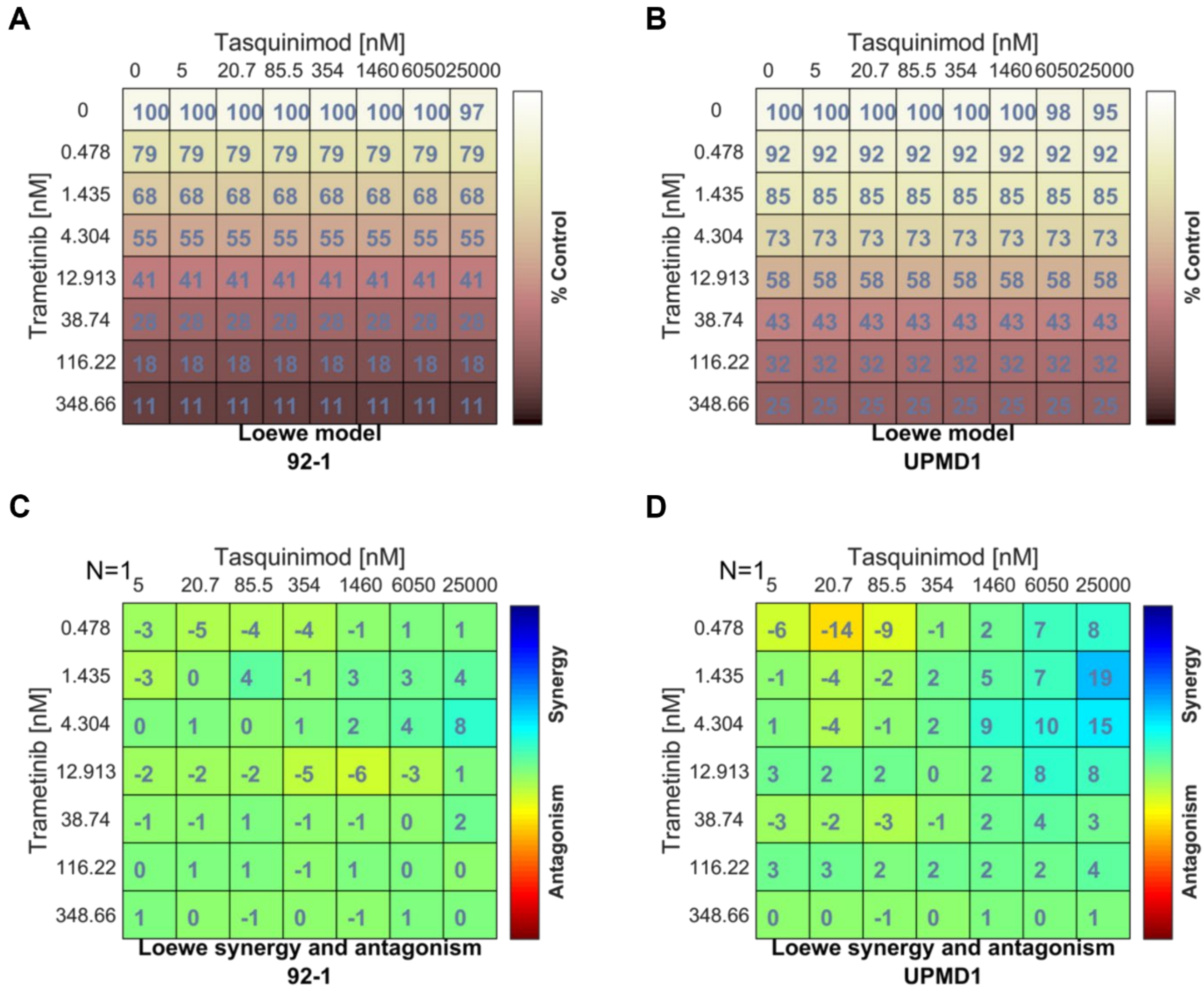

Figure S9. HDAC4 inhibition and MEK inhibition do not show synergistic effects in UM cell lines. Cell viability of UM cell lines 92-1 and UPMD1 remains unaffected by treatment with combinations of HDAC4 inhibitor tasquinimod and MEK inhibitor trametinib for 5 days compared to single treatments (**A, B**), despite calculated synergy scores according to the Loewe model (**C, D**). Cell viability is expressed as normalized values in percentage to DMSO controls. Summarized results of one experiment are shown. Synergy scores and graphs are generated with Combenefit[2].

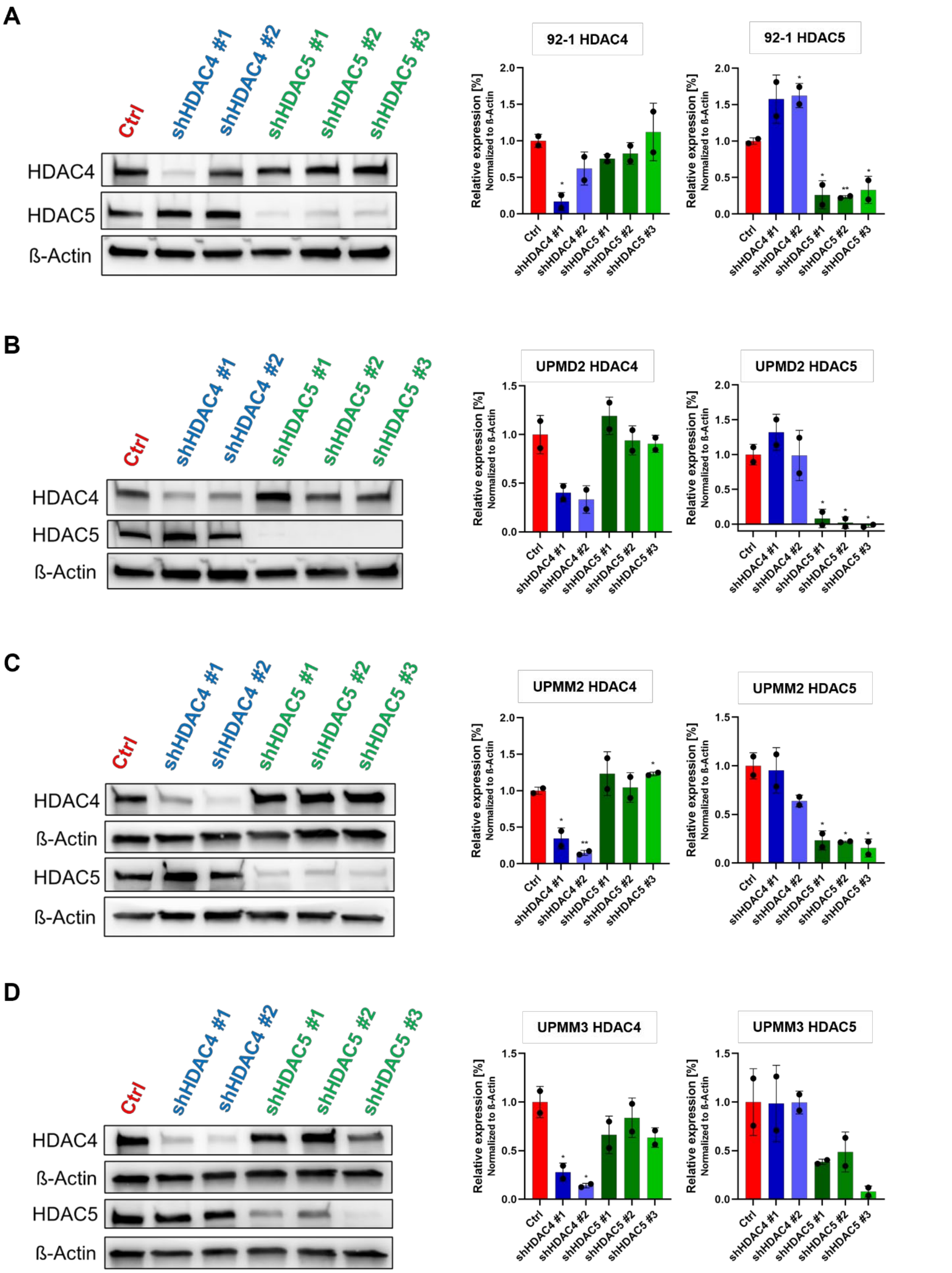

Figure S10. Knockdown validation of *HDAC4* or *HDAC5* in UM cell lines. Western blots with quantifications for HDAC4 or HDAC5 in cell lines 92-1 (**A**), UPMD2 (**B**), UPMM2 (**C**) and UPMM3 (**D**) upon shRNA knockdown. A representative blot and quantifications of summarized results of two independent experiments is shown. Protein expression is normalized to β-Actin followed by normalization to control levels (Ctrl). Data are average ± SD. *, *P*<0.05. **, *P*<0.01.

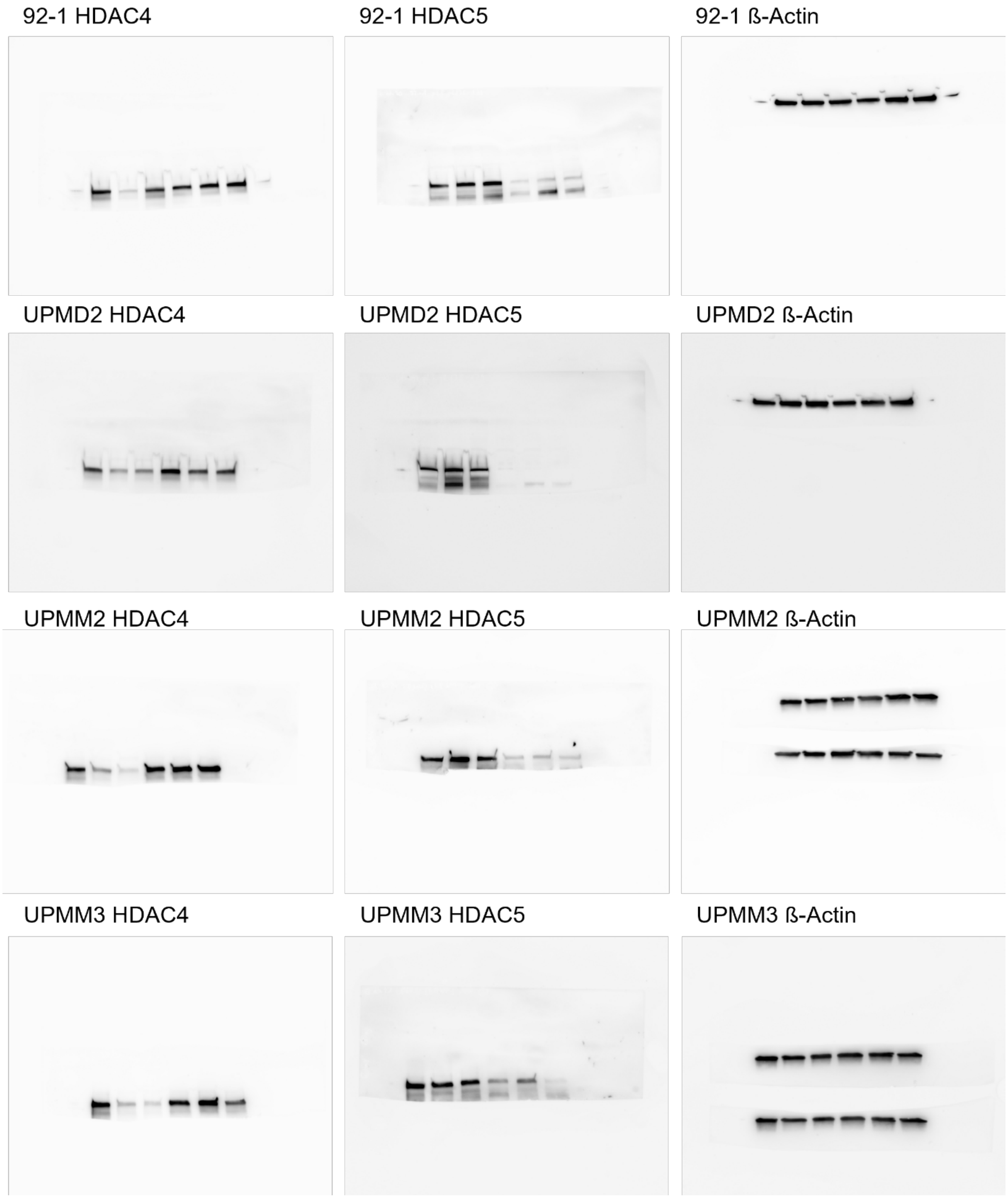

Figure S11. Un-cropped western blots. Original pictures of the western blots shown in Supplementary Figure S10.

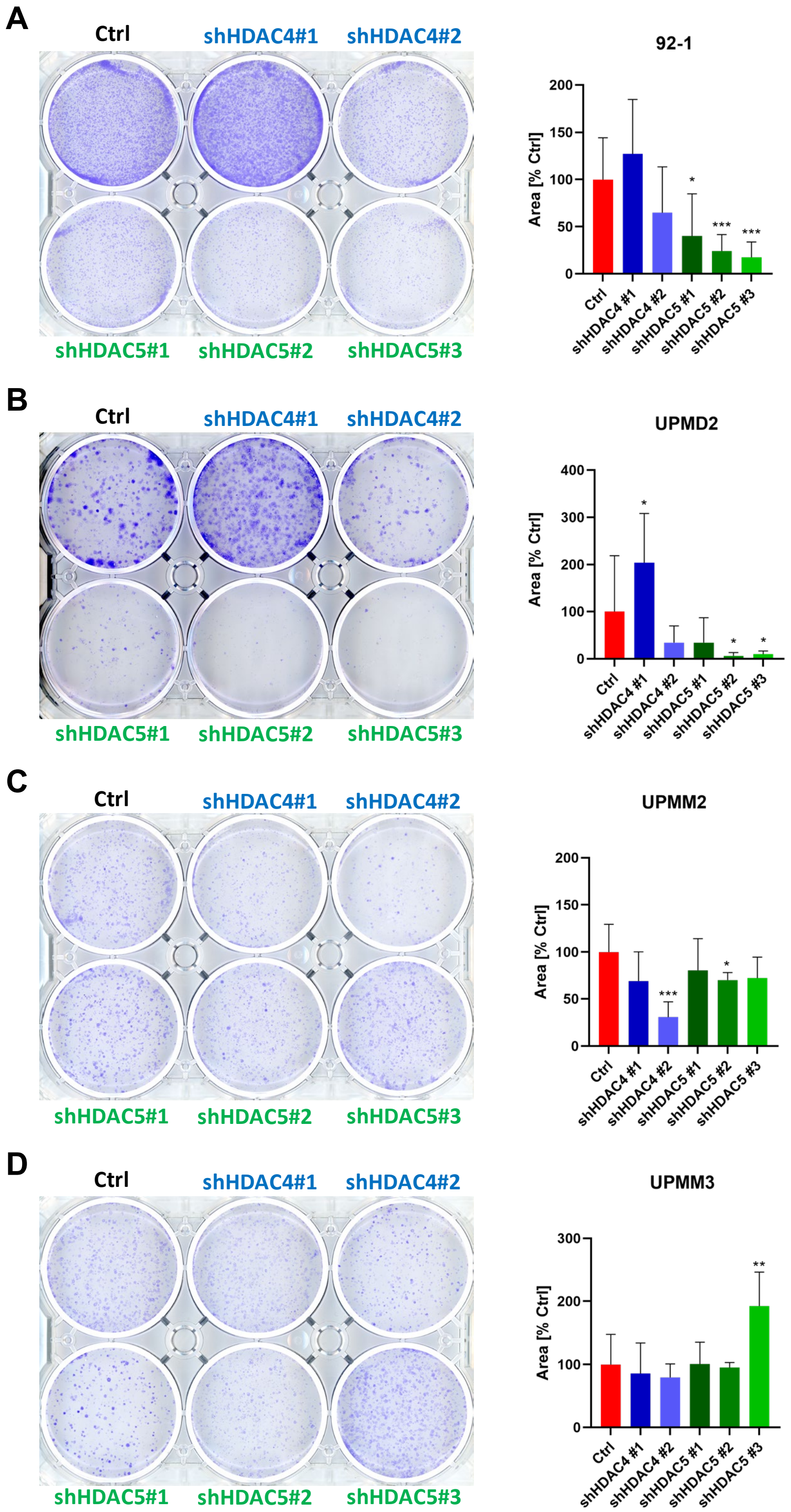

Figure S12. Colony formation ability is not consistently affected by knockdown of *HDAC4* or *HDAC5* in uveal melanoma cell lines. Colony formation assays and quantifications of UM cell lines 92-1 (**A**), UPMD2 (**B**), UPMM2 (**C**) and UPMM3 (**D**) upon knockdown of *HDAC4* or *HDAC5*. A representative picture and quantifications of summarized results of two to four independent experiments is shown. Confluency is normalized to controls (Ctrl). Data are average ± SD. *, P<0.05. **, P<0.01. ***, P<0.001.

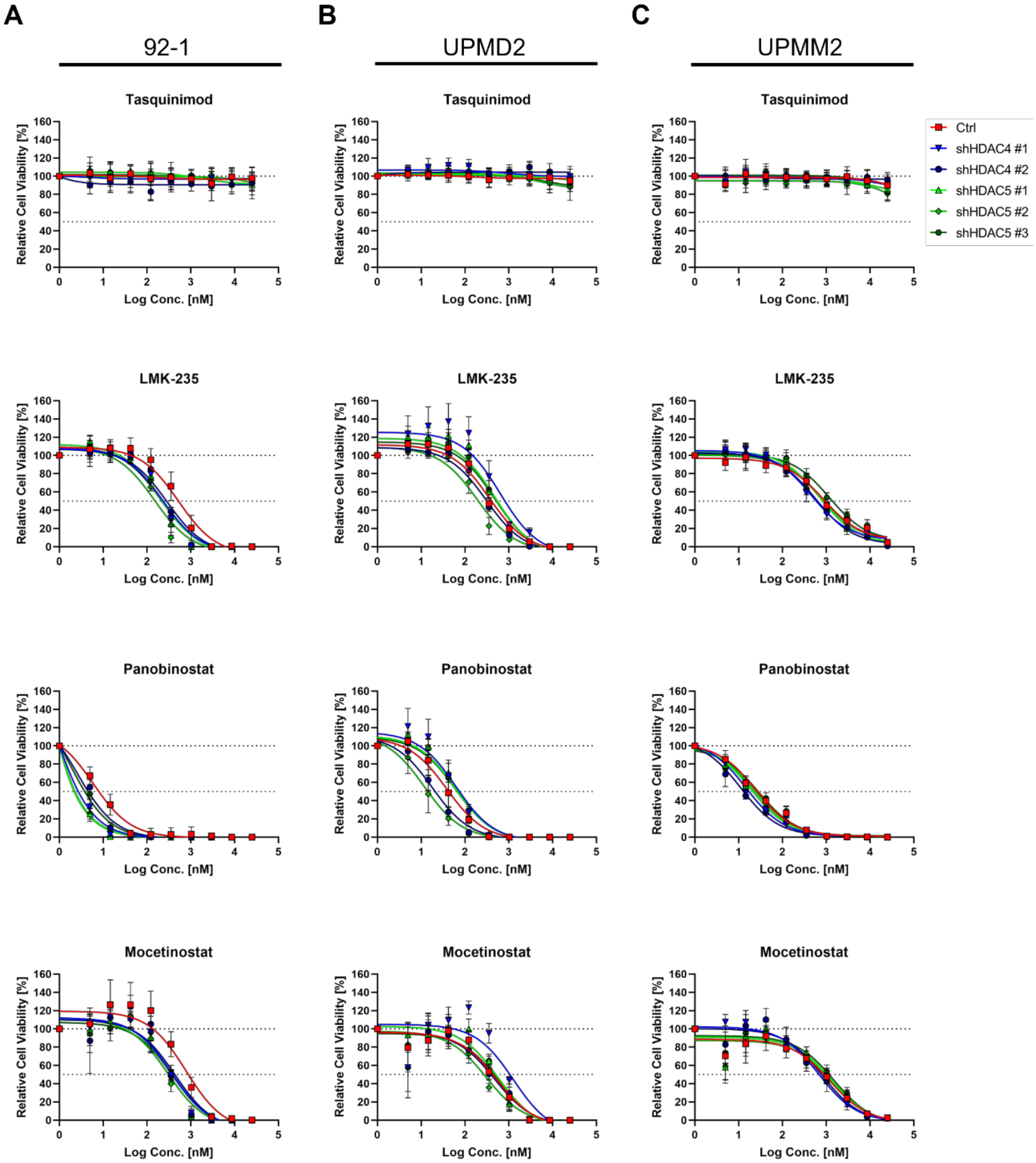

Figure S13. Sensitivity to HDAC inhibition is not driven by inhibition of HDAC4 or HDAC5. Dose-response curves of UM cell lines 92-1 (**A**), UPMD2 (**B**) and UPMM2 (**C**) upon shRNA knockdown of *HDAC4* or *HDAC5* treated with the HDAC4 inhibitor tasquinimod, the HDAC4/5 inhibitor LMK-235, the pan-HDAC inhibitor panobinostat or the HDAC inhibitor mocetinostat (from top to bottom) at different concentrations (from 5 nM to 25 µM) for 5 days. Cell viability is expressed as normalized values in percentage to DMSO controls. Summarized results of two to three independent experiments are shown. Data are average ± SD.

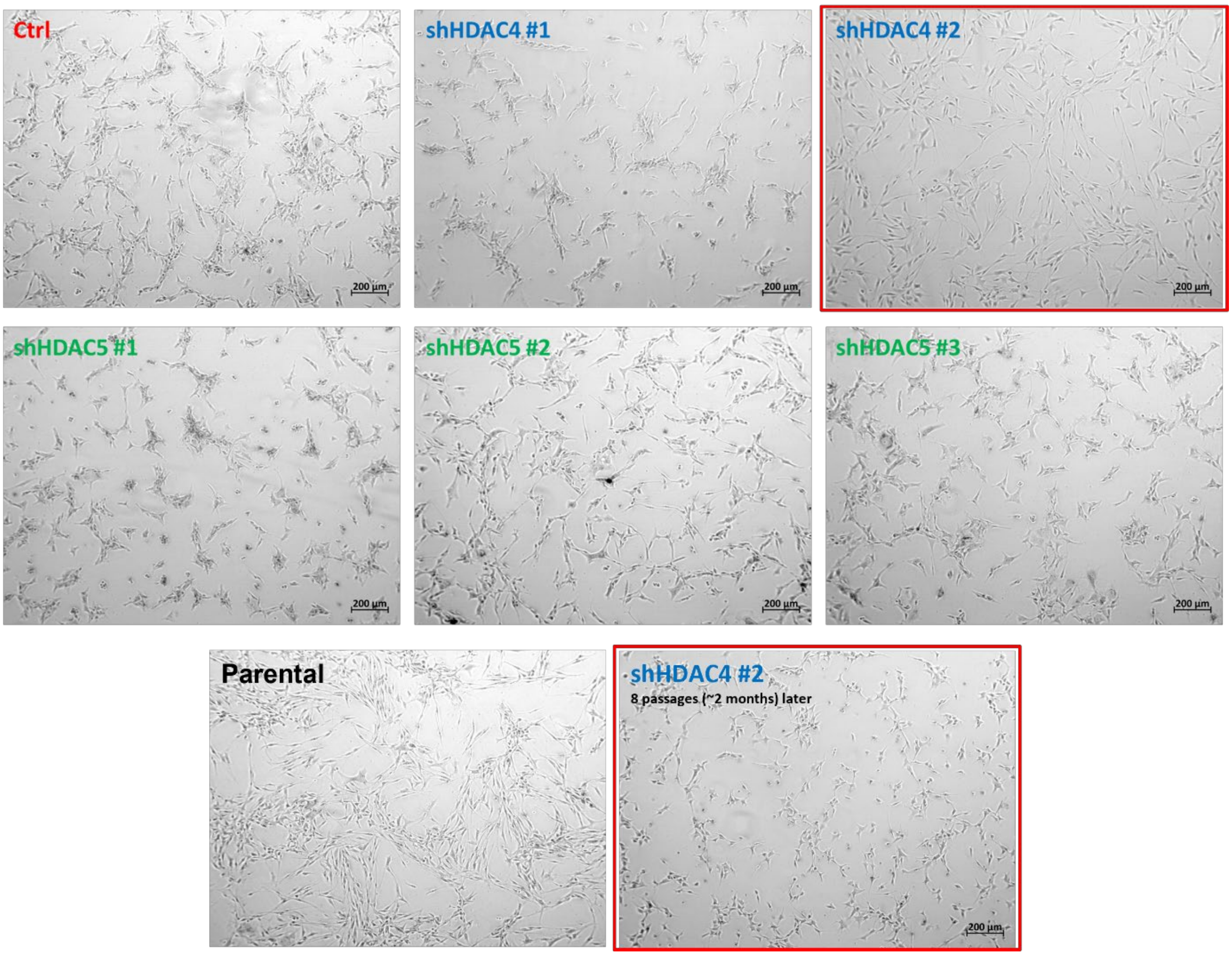

Figure S14. UPMM2 undergoes morphological transition after transduction with a delay in sh*HDAC4* #2. All transduced UPMM2 variants underwent morphological changes quickly after transduction, except for UPMM2 sh*HDAC4* #2 (red frame), where morphological transition was delayed for several passages. Brightfield microscopy images. Magnification: 40x. Scale bar: 200 µm.

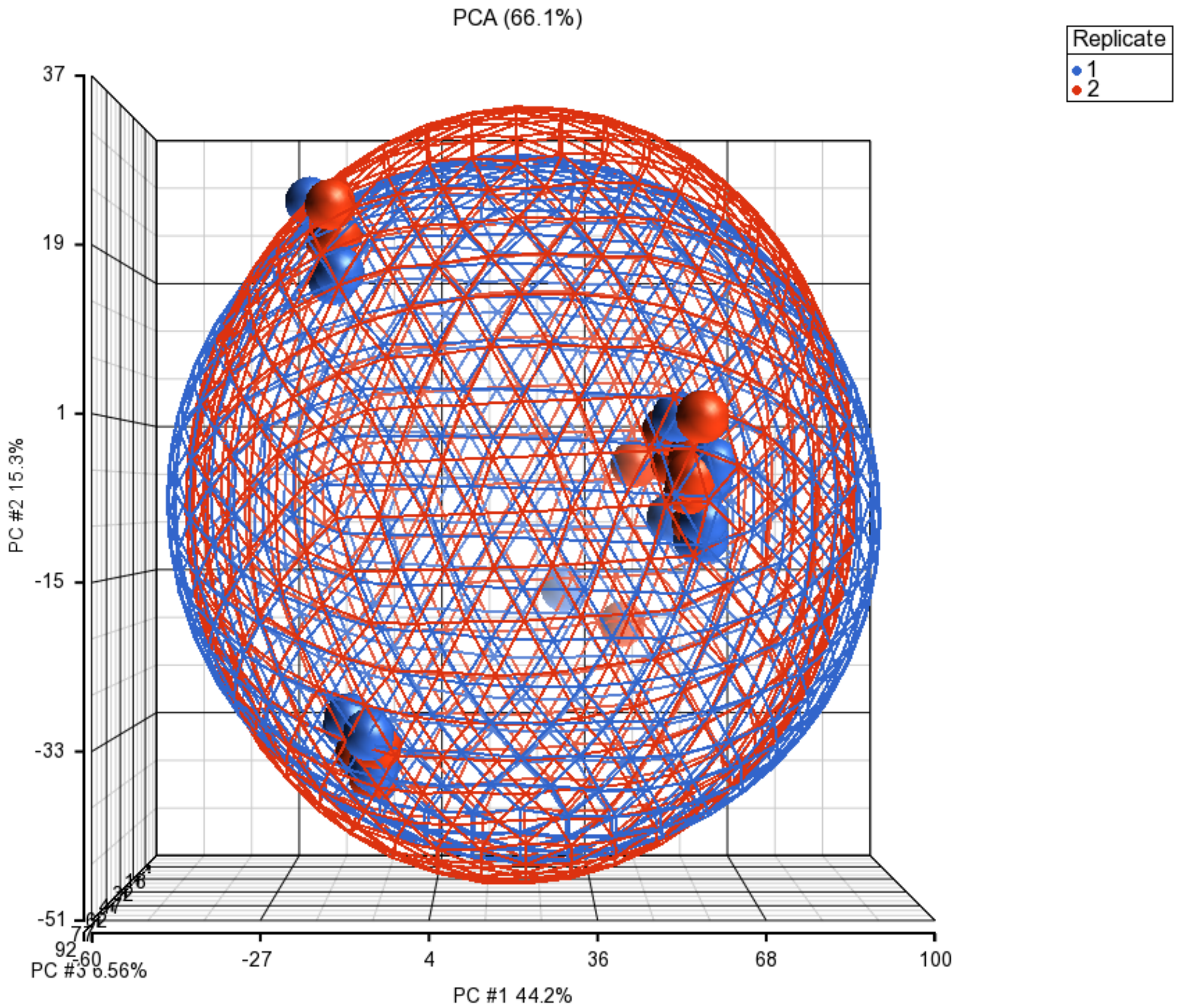

Figure S15. Biological replicates do not constitute a source of variation in gene expression analysis. Principal Component Analysis (PCA) shows low variation between biological replicates of samples in gene expression analysis and can therefore be excluded as a potential source of error.

(Next page) Figure S16. Proteogenomic analysis of BAP1-dependent differentiation genes revealed that *HDAC4* and *HDAC5* knockdown had no significant effect. Non-significant BAP1-dependent differentiation genes when comparing D3 vs. M3 cells show that knocking down *HDAC4* or *HDAC5* has a marginal effect, since UM cell lines retain their D3 or M3 characteristics. FDR, false-discovery rate corrected *P* value; FC, fold change. The significant BAP1-dependent differentiation genes are displayed in Figure 7a.

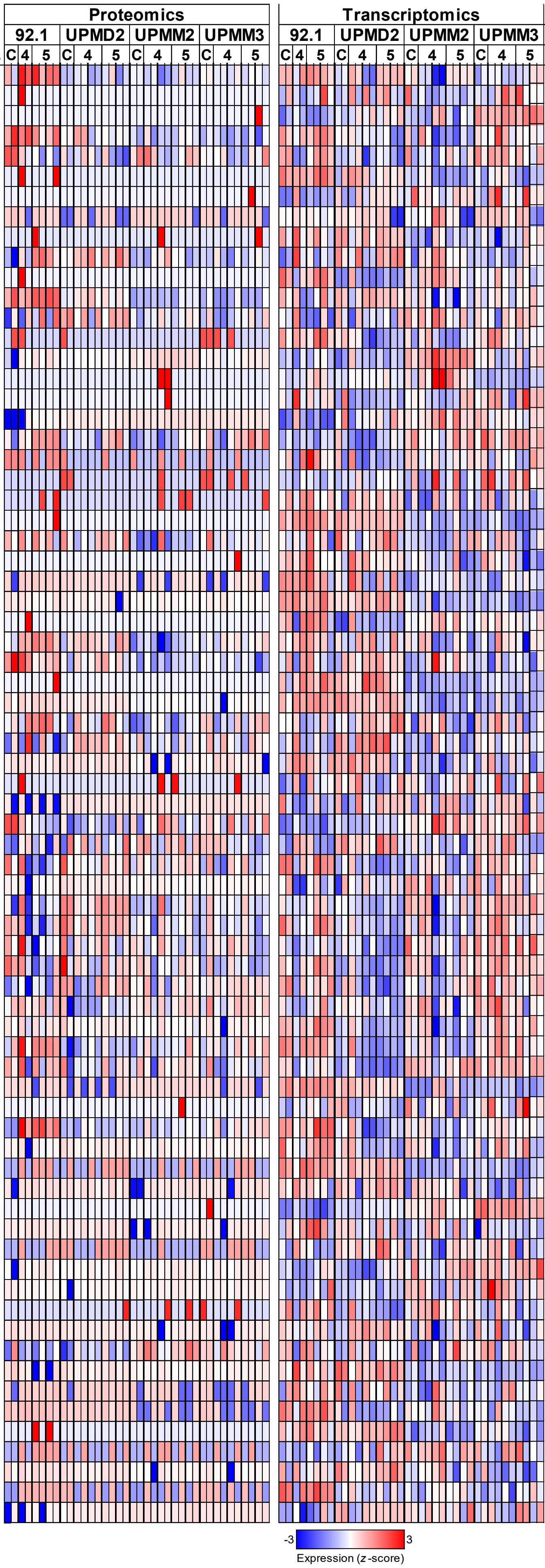

Gene names	Proteomics D3 vs. M3 P	FDR q	FC	Transcriptomics D3 vs. M3 P	FDR q	FC
API5	0.048	0.13	-1.0	0.012	0.028	-1.2
ARHGEF16	0.30	0.42	-2.2	0.83	0.88	-1.0
BACE2	0.35	0.42	2.1	0.009	0.021	1.3
BTF3	7E-6	7E-5	-1.0	0.25	0.36	-1.0
C12orf10	0.34	0.42	1.0	0.11	0.18	1.1
C19orf43	0.13	0.26	-3.6	8E-8	6E-7	-1.3
CELSR1	0.35	0.42	2.0	0.011	0.027	1.2
CERS2	0.20	0.36	1.3	0.87	0.91	1.0
CREB1	0.64	0.70	1.4	1E-4	5E-4	-1.1
DDB1	0.014	0.046	-1.0	0.25	0.36	-1.1
DNAJB6	0.30	0.42	-2.3	0.22	0.33	1.1
EEF2	3E-8	6E-7	-1.0	0.047	0.09	-1.0
EIF3A	0.09	0.20	-1.0	0.014	0.031	1.1
FAU	0.82	0.86	1.1	0.70	0.78	-1.0
FNDC3A	0.10	0.23	1.1	2E-5	7E-5	1.7
FZD7	0.18	0.33	3.5	0.64	0.74	1.8
GIGYF1	0.35	0.42	2.1	0.21	0.32	1.1
GORASP2	0.043	0.12	1.2	0.002	0.005	1.2
GSPT1	0.33	0.42	-1.0	2E-7	1E-6	1.1
GTPBP4	0.19	0.34	-1.7	0.69	0.77	1.0
HIST1H1B	0.28	0.42	1.9	0.018	0.040	1.8
MKI67	0.53	0.60	1.5	0.010	0.023	-1.6
NCOA5	0.30	0.42	-2.2	2E-14	8E-13	-2.0
NOP2	0.006	0.024	-1.0	0.09	0.16	-1.1
NRF1	0.35	0.42	2.1	0.001	0.003	-1.2
NUP85	0.05	0.14	-1.3	0.001	0.003	-1.4
PABPN1	0.52	0.59	1.0	2E-12	4E-11	-1.4
PAXIP1	0.30	0.42	-2.4	0.14	0.22	1.1
PDE12	5E-4	0.003	-1.0	0.043	0.08	-1.1
PPIA	2E-5	2E-4	-1.0	0.14	0.22	-1.0
PRCC	0.30	0.42	-2.2	7E-10	8E-9	-1.3
PRMT1	0.041	0.12	-1.1	1E-13	3E-12	-1.6
PRPF6	0.020	0.07	-1.0	0.23	0.34	-1.1
PSMD4	0.87	0.90	-1.0	0.025	0.05	-1.1
PTMS	0.045	0.13	-1.2	8E-5	3E-4	-1.3
PTRH1	0.36	0.43	1.9	0.51	0.62	1.0
PURB	0.040	0.11	1.3	0.65	0.74	1.0
RAB1A	0.17	0.32	1.0	2E-9	2E-8	1.2
RAD23B	0.26	0.42	1.0	0.16	0.24	-1.0
RPL10	0.72	0.77	-1.0	0.90	0.93	1.0
RPL13A	0.56	0.63	1.0	0.039	0.08	1.1
RPL14	0.16	0.31	-1.0	0.027	0.06	1.1
RPL18	0.09	0.20	-1.0	0.94	0.96	-1.0
RPL22	0.78	0.82	-1.0	3E-5	1E-4	1.2
RPL27A	0.15	0.30	-1.0	0.040	0.08	1.2
RPL28	0.30	0.42	1.0	0.17	0.27	1.0
RPS11	0.08	0.18	1.0	0.13	0.21	1.0
RPS13	0.23	0.41	-1.1	0.82	0.88	-1.0
RPS16	0.83	0.87	-1.0	0.85	0.89	1.0
RPS2	0.53	0.59	-1.0	1E-6	7E-6	1.1
RPS26	0.17	0.32	1.2	2E-9	2E-8	-1.4
RPS29	0.35	0.42	2.2	0.10	0.17	1.1
RPS3	0.38	0.45	-1.0	0.48	0.60	-1.0
RPS9	0.49	0.56	1.0	0.59	0.69	-1.0
SF3B4	0.20	0.36	-1.5	2E-13	6E-12	-1.4
SMG1	0.35	0.42	-1.1	0.022	0.047	-1.4
SOWAHC	0.35	0.42	2.1	3E-9	3E-8	2.1
SRSF11	0.06	0.15	-1.2	0.018	0.040	-1.1
SRSF9	0.21	0.38	-1.5	0.33	0.45	-1.1
SSR1	0.28	0.42	1.1	6E-6	3E-5	1.4
TCEB2	0.26	0.42	1.1	0.003	0.007	1.1
TIMM23	0.20	0.36	2.4	0.44	0.56	1.0
TTC1	0.05	0.14	-1.2	0.024	0.05	-1.1
TUBB	2E-5	1E-4	1.0	0.95	0.96	1.0
UBTF	0.32	0.42	1.1	3E-10	4E-9	-1.4
UCK2	0.08	0.18	-1.4	0.08	0.14	-1.1
WDR43	2E-4	0.001	-2.0	0.40	0.52	-1.1
YTHDF1	0.13	0.26	-3.6	3E-8	2E-7	-1.3
ZBTB10	0.54	0.60	-1.2	0.010	0.024	1.2
ZFR	0.06	0.15	-1.1	0.002	0.005	-1.1
ZNF207	0.27	0.42	-1.3	0.015	0.034	-1.1
ZYX	0.049	0.13	1.2	0.21	0.31	1.1

Figure S16.

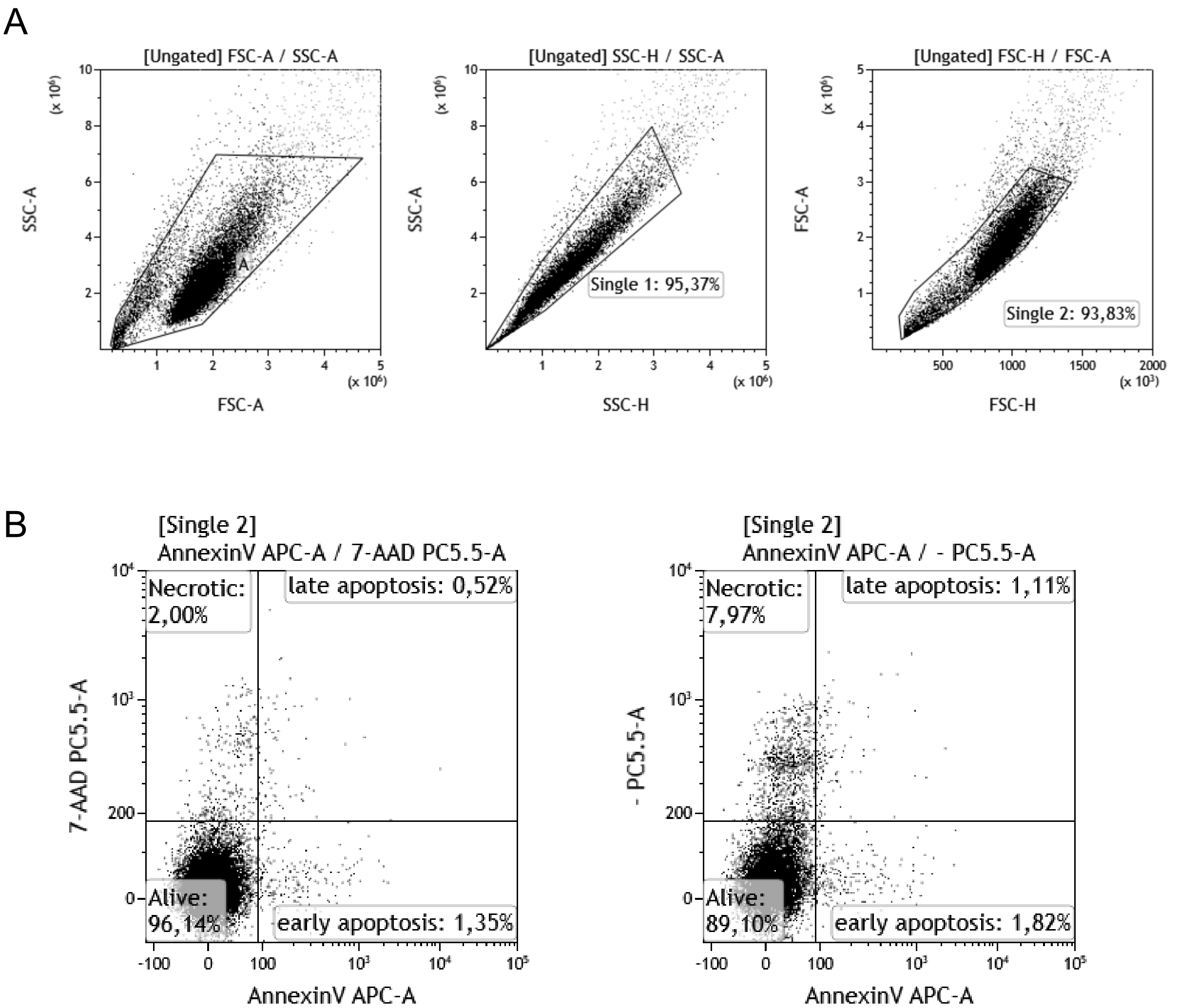

Figure S17. Representative gating strategy of flow cytometric analysis of apoptosis and necrosis levels. (**A**) Cells were sequentially gated using forward and side scatter (FSC/SSC) profiles to exclude debris and doublets prior to downstream analysis. (**B**) Representative plots of UPMD2 sh*HDAC4* #1 and UPMM2 sh*HDAC4* #1 illustrate the gating strategy for Annexin V- and 7-AAD-stained cells. Annexin V-positive but 7-AAD-negative cells indicate early apoptosis, whereas cells positive for both indicate late apoptosis. The two populations were summed to determine the total percentage of apoptotic cells. Necrotic cells were defined as 7-AAD-positive but Annexin V-negative events. The final percentages for both cell death pathways were calculated by subtracting the background signal determined from parallel unstained controls.

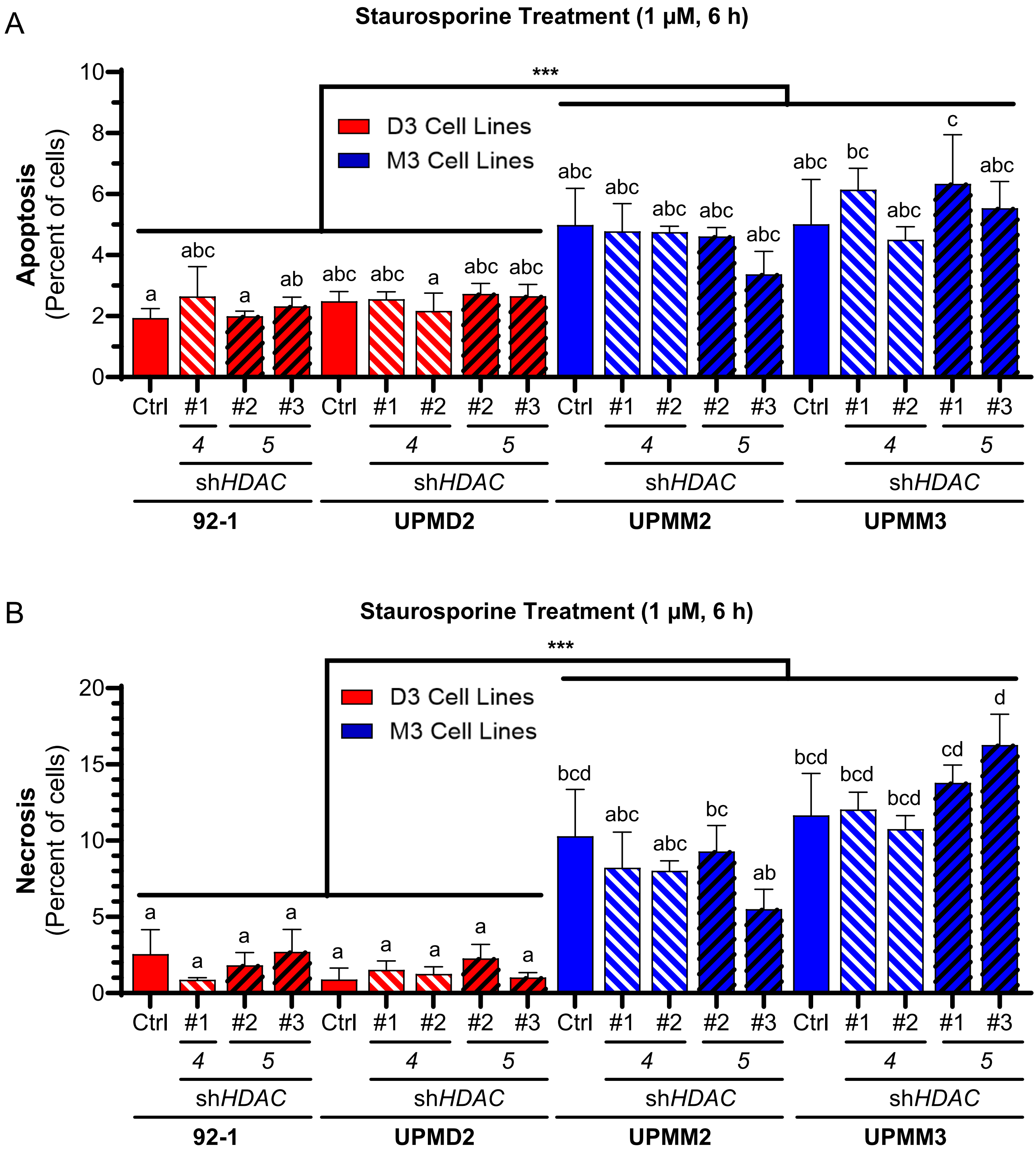

Figure S18. Flow cytometric analysis of apoptosis and necrosis levels after staurosporine treatment. Cell lines were analyzed by flow cytometry to assess the levels of apoptosis (**A**) and necrosis (**B**) after treatment with 1 μM staurosporine for 6 h. Data are presented in percentage of cells positive for Annexin V (apoptosis) or 7-AAD (necrosis) for the indicated conditions. Data represent the average ± SE (n=3). ***, $P < 0.001$ by two-factor ANOVA. Non-overlapping letters (e.g., “a” vs. “b”) represent significant differences ($P < 0.05$) by one-factor ANOVA and Student-Newman-Keuls *post hoc* test.

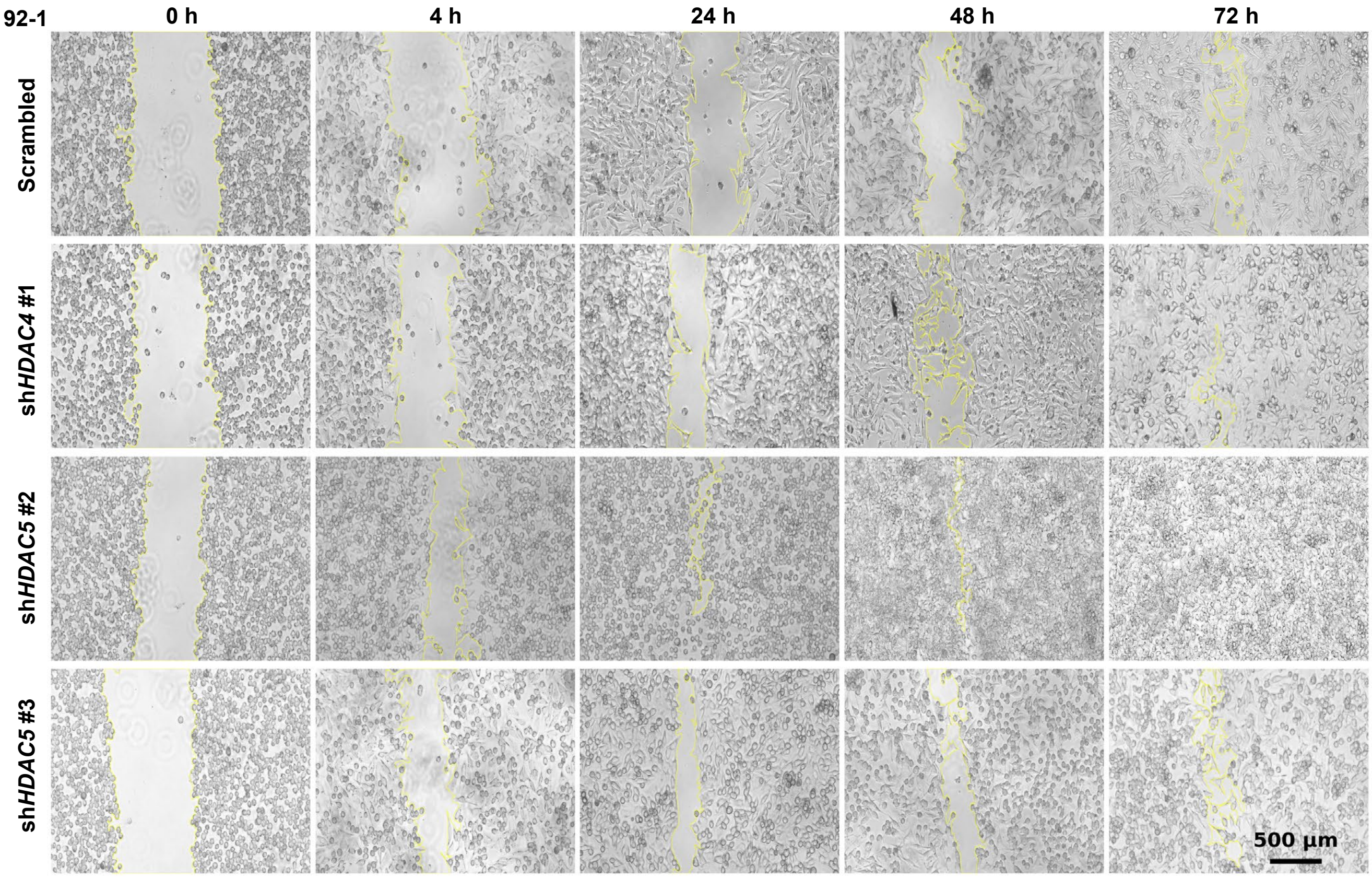

Figure S19. Wound-healing assay shows differences in gap closure due to *HDAC4* and *HDAC5* knockdown in 92-1 cells. Representative images of four technical replicates in three independent biological experiments. Gap closure was quantified using Fiji/ImageJ and displayed in Figure 7d.

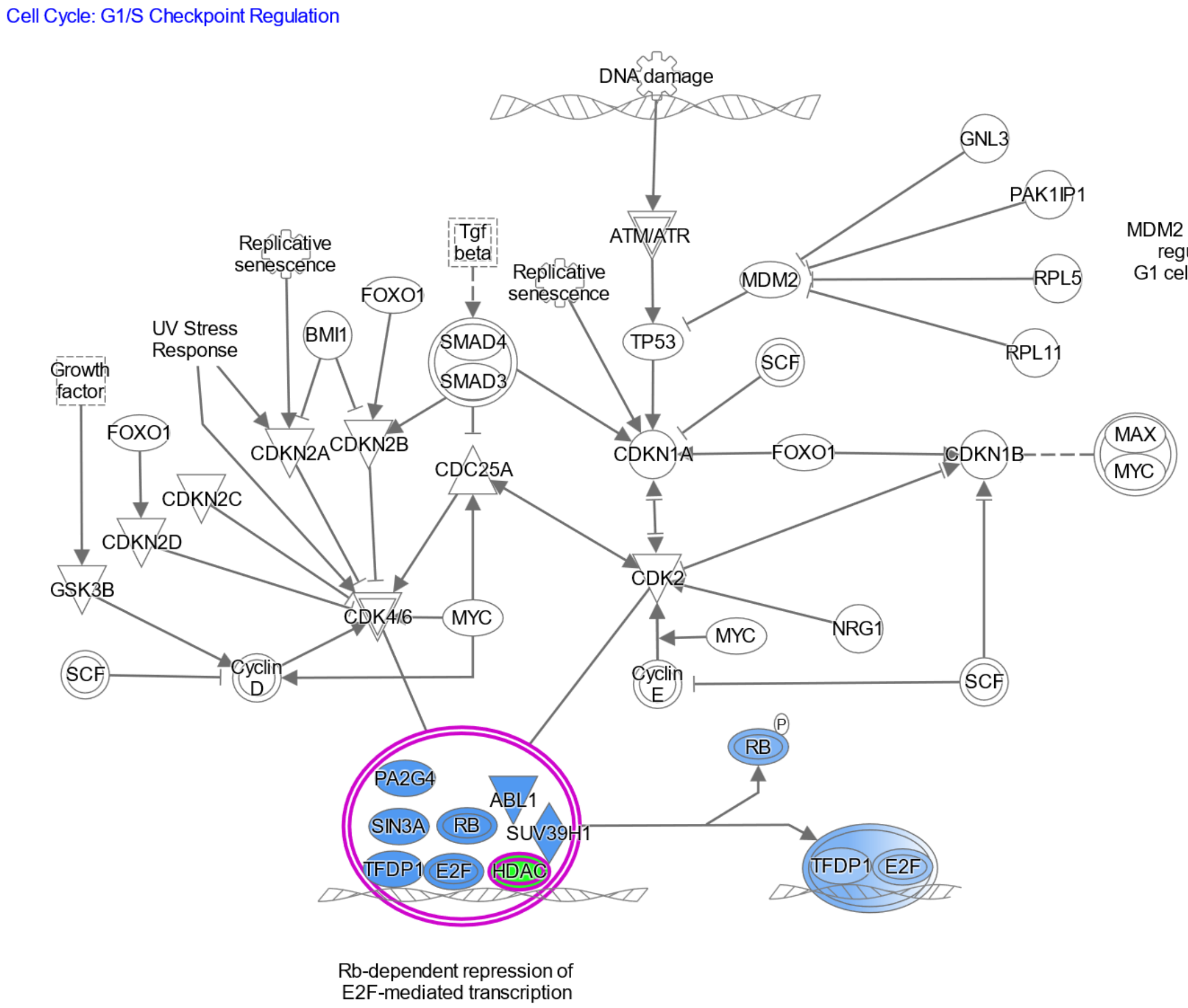

© 2000-2022 QIAGEN. All rights reserved.

Figure S20. Cell Cycle: G1/S Checkpoint Regulation Signaling upon knockdown of *HDAC4*. Ingenuity Pathway Analysis shows downregulated genes in blue/green (predicted/measured) and upregulated genes in orange/red (predicted/measured).

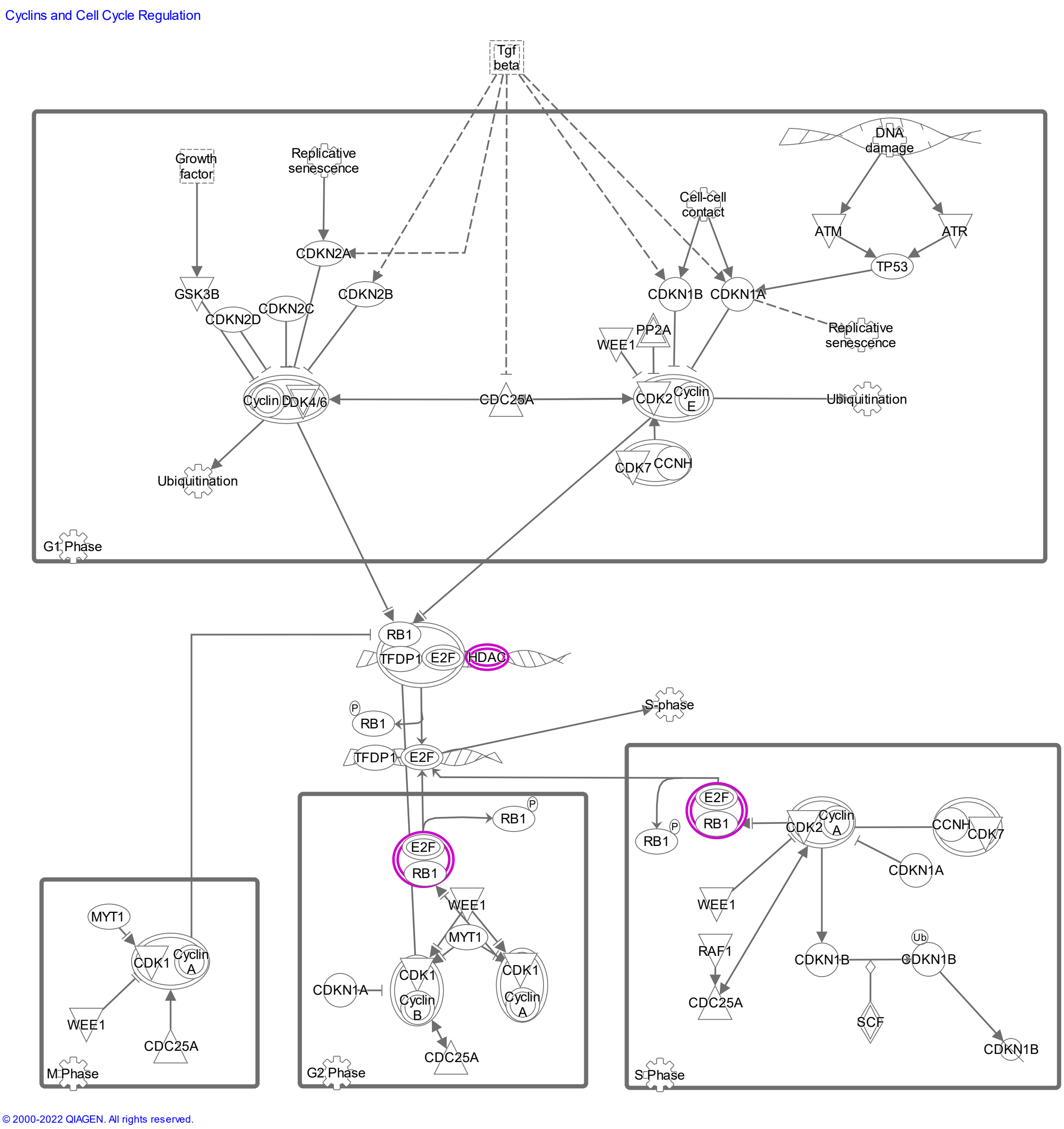

Figure S21 Cyclins and Cell Cycle Regulation Signaling upon knockdown of *HDAC4*. Ingenuity Pathway Analysis shows downregulated genes in blue/green (predicted/measured) and upregulated genes in orange/red (predicted/measured).

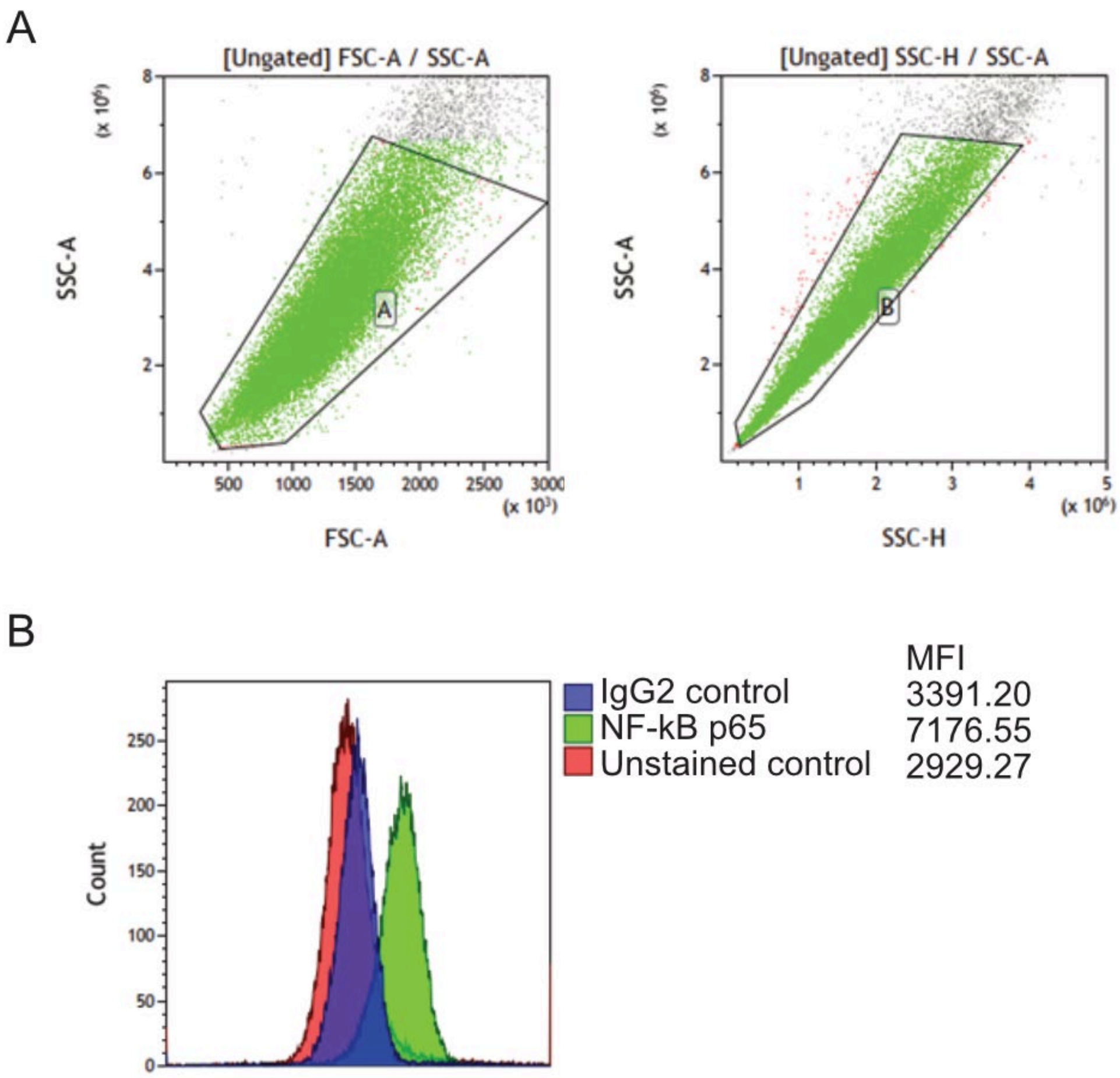

Figure S22. Representative phospho-flow cytometric analysis of NF-κB p65 activation. (**A**) Cells were gated sequentially to exclude debris and doublets prior to analysis. (**B**) Representative overlaid histograms display intracellular NF-κB p65 phosphorylation levels measured by phospho-flow cytometry. Shown are unstained cells, IgG2b isotype control, and NF-κB p65–stained samples. Quantification was performed using mean fluorescence intensity (MFI).

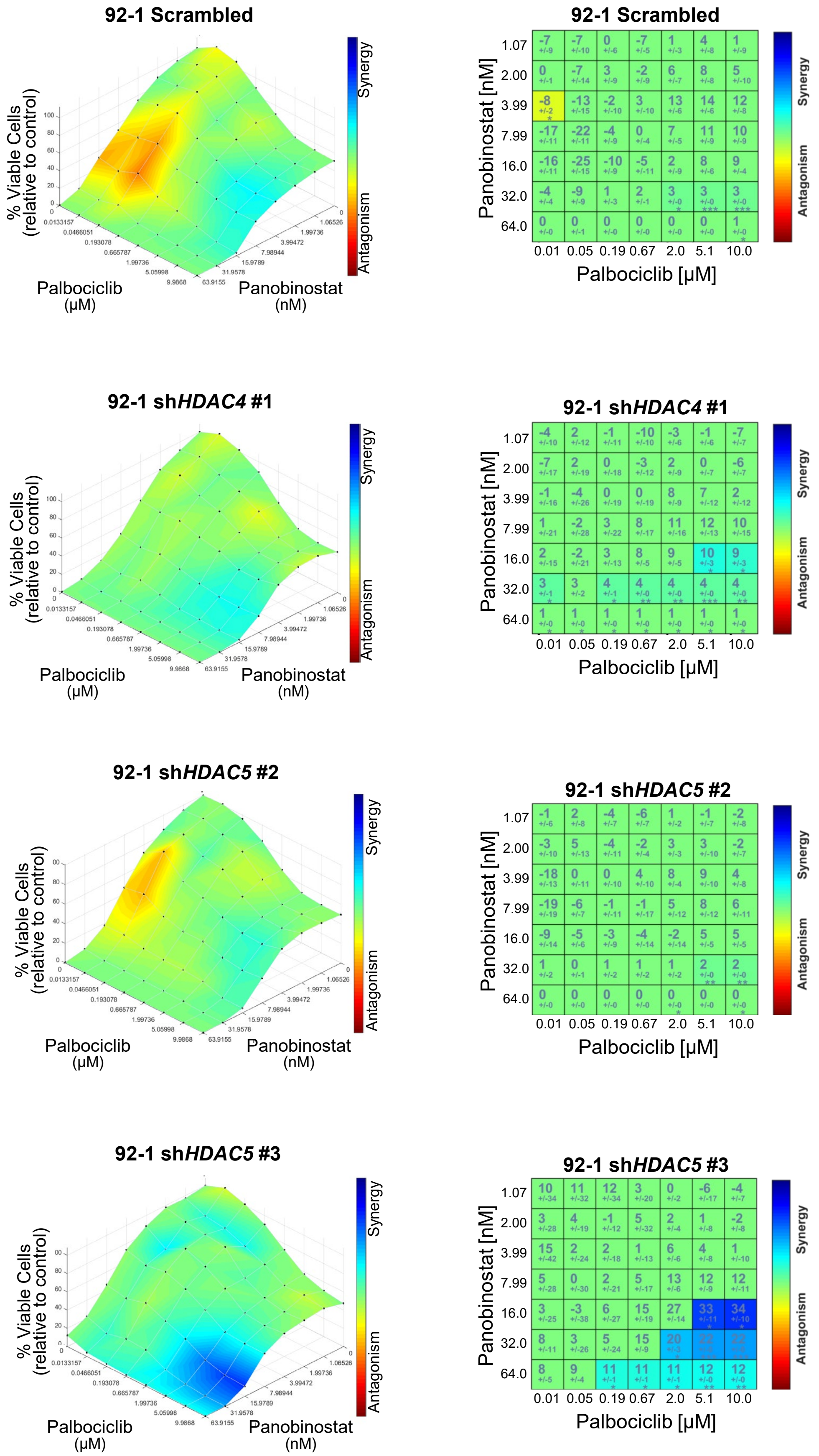

Figure S23. *HDAC5* #3 knockdown sensitizes 92-1 cells to the combination of panobinostat and palbociclib. Cell viability of 92-1 cells after treatment with combinations of pan-HDAC inhibitor panobinostat and palbociclib for 5 days and respective synergy scores according to the Loewe model. Cell viability is expressed as normalized values in percentage to DMSO controls. Summarized results of three independent experiments are shown. Synergy scores and graphs are generated with Combenefit[2].

Supplementary Table S1. List of compounds.

Compound	Mode of action	Supplier	Catalogue #
Bortezomib	Proteasomal inhibitor	Selleck Chemicals	S1013
Borussertib (RL1784)	AKT inhibitor	Prof. Dr. Daniel Rauh, Technical University Dortmund	-
Carfilzomib	Proteasomal inhibitor	Selleck Chemicals	S2853
Dacarbazine	Cytostatic (alkylating agent)	Sigma-Aldrich	D2390
Erastin	Ferroptosis inducer	Sigma-Aldrich	E7781
Fotemustin	Cytostatic (alkylating agent)	LKT Laboratories	F5976
JQ1	BET inhibitor	BPS Bioscience	27402
LDC000526:15	CDK9 inhibitor	Lead Discovery Center, Dortmund	-
LDC208447:01	CDK9 inhibitor	Lead Discovery Center, Dortmund	-
LMK-235	HDAC4/5 inhibitor	Hölzel Diagnostika	M4986
Mocetinostat	HDAC class I + IV inhibitor	Selleck Chemicals	S1122
MU-1210	CLK inhibitor	Prof. Dr. Stefan Knapp, University of Frankfurt	-
Palbociclib	CDK4/6 inhibitor	Selleck Chemicals	S1116
Panobinostat	pan-HDAC inhibitor	Selleck Chemicals	S1030
Refametinib	MEK inhibitor	Selleck Chemicals	S1089
SN-38	Topoisomerase I inhibitor	Cayman Chemical Company	15632
Sunitinib	Tyrosine kinase inhibitor	Selleck Chemicals	S7781
T3-CLK	CLK inhibitor	Prof. Dr. Stefan Knapp, University of Frankfurt	-
Tasquinimod	HDAC4 inhibitor	Cayman Chemical Company	17692
TP-238	CECR2 inhibitor	Prof. Dr. Stefan Knapp, University of Frankfurt	-
Trametinib	MEK inhibitor	Selleck Chemicals	S2673
TW9	Dual BET/HDAC inhibitor	Prof. Dr. Stefan Knapp, University of Frankfurt	-
Verteporfin	Photosensitizer	Hölzel Diagnostika	A12658

Supplementary Table S2. List of plasmid targeted sequences.

Name	Description	Insert / Targeted Sequence	Supplier	ID
pLKO.1-puro	Empty vector	-	Sigma-Aldrich	SHC001
pLKO.1-Sc	Scrambled control	CCGGCAACAAGATGAAGAGCACCAACTCGAGTTGGTGCTCTTCATCTTGTTGTTTTT	Sigma-Aldrich	SHC002
pLKO.1-Sc #2	Scrambled control #2	GGGTCTGTATAGGTGGAGACTCGAGTCTCCACCTATACAGACCC	Gardner Lab, New York University School of Medicine, NY, USA	Nemetski *et al.,* J Biol Chem, 2007[3]
pLKO.1-sh*HDAC4* #1	shRNA	CCGGGCCAAAGATGACTTCCCTCTTCTCGAGAAGAGGGAAGTCATCTTTGGCTTTTT	Sigma-Aldrich	TRCN0000004832
pLKO.1-sh*HDAC4* #2	shRNA	CCGGGAATCTGAACCACTGCATTTCCTCGAGGAAATGCAGTGGTTCAGATTCTTTTTG	Sigma-Aldrich	TRCN0000314667
pLKO.1-sh*HDAC5* #1	shRNA	CCGGGCTAGAGAAAGTCATCGAGATCTCGAGATCTCGATGACTTTCTCTAGCTTTTT	Sigma-Aldrich	TRCN0000004835
pLKO.1-sh*HDAC5* #2	shRNA	CCGGCTACGACACGTTCATGCTAAACTCGAGTTTAGCATGAACGTGTCGTAGTTTTTG	Sigma-Aldrich	TRCN0000244923
pLKO.1-sh*HDAC5* #3	shRNA	CCGGCCATCGCTGAGAATGGCTTTACTCGAGTAAAGCCATTCTCAGCGATGGTTTTTG	Sigma-Aldrich	TRCN0000244922

Supplementary Table S3. **Proteogenomic analysis of D3 *vs.* M3 cell lines with *P* < 0.05.**

		Transcriptomics			
**Proteomics**	***P*<0.05**	**FC<1**	**FC>1**	**NS**	**Total**
	**FC<1**	**852**	**100**	257	1,209
	**FC>1**	**49**	**744**	204	997
	**NS**	1,207	1,163	1,220	3,590
	**Total**	2,108	2,007	1,681	5,796

FDR, false-discovery rate; FC, fold change; NS, non-significant.

Supplementary Table S4. Recurrent significant pathways by *HDAC4* and *HDAC5* knockdowns for genes identified in both proteomics and transcriptomics.

	sh*HDAC4*							sh*HDAC5*						
				D3		M3					D3		M3	
**Ingenuity Canonical Pathways**	**All**	**D3**	**M3**	**91-2**	**UPMD2**	**UPMM2**	**UPMM3**	**All**	**D3**	**M3**	**91-2**	**UPMD2**	**UPMM2**	**UPMM3**
**Cell Cycle: G1/S Checkpoint Regulation**	0.034	0.047	0.029		0.004	0.013	0.016	0.05	0.040	0.07		0.09	0.013	0.013
**Cyclins and Cell Cycle Regulation**	0.043	0.002	0.036		0.006	0.017	0.020	0.06	0.049	0.09		0.11	0.017	0.017
Pancreatic Adenocarcinoma Signaling	0.06	0.004	0.05		0.013	0.025	0.030	0.09	0.003	0.14		0.16	0.025	0.025
Prostate Cancer Signaling	0.06	0.003	0.05		0.011	0.023	0.028	0.09	0.002	0.13		0.15	0.023	0.023
CNTF Signaling		0.040						0.042	0.033		0.037			0.040
Role of NFAT in Cardiac Hypertrophy	0.11	0.011	0.10	0.11	0.27	0.045	0.05	0.16	0.008	0.23		0.004	0.12	0.15
**ERK/MAPK Signaling**		0.010			0.035			0.15			0.14	0.003		0.14
**Xenobiotic Metabolism Signaling**	0.14	0.018	0.12		0.007	0.06	0.07	0.20	0.16	0.29		0.33	0.011	0.19
**Inflammasome pathway**	0.011	0.015			0.029									
**Melanocyte Development and Pigmentation Signaling**		0.07		0.05				0.003	0.06		0.002	0.008		
**Role of IL-17F in Allergic Inflammatory Airway Diseases**								0.035	0.028		0.031			
Axonal Guidance Signaling	0.23	4E-4		0.23	0.15			0.32	0.003			0.033		
Cardiac Hypertrophy Signaling (Enhanced)	0.25	0.007	0.21	0.25	0.53	0.10	0.12	0.34	0.041	0.47		0.040	0.26	0.06
EIF2 Signaling		0.011			0.27				0.008			0.040		
Leptin Signaling in Obesity		0.05		0.040					0.046			0.005		0.05
ERK5 Signaling					0.10			0.05	0.044		0.049			
Gluconeogenesis I					0.06				0.028		0.032			
Glycolysis I					0.06				0.025		4E-4			
Growth Hormone Signaling								0.05	0.044		0.049	0.10		0.05
Semaphorin Neuronal Repulsive Signaling Pathway		0.10						0.11	0.004			0.019		
Non-Small Cell Lung Cancer Signaling	0.05	0.002	0.043		0.13	0.019	0.023	0.07	0.002	0.11		0.008	0.019	0.019
Small Cell Lung Cancer Signaling	0.049	0.07	0.042		0.008	0.019	0.023	0.07	0.002	0.11		0.13	0.019	0.019
Chronic Myeloid Leukemia Signaling	0.05	0.003	0.046		0.13	0.021	0.025	0.08	0.002	0.12		0.14	0.021	0.021
**Telomerase Signaling**	0.05	6E-5	0.046		0.13	0.021	0.025	0.08	0.002	0.12		0.14	0.021	0.021
**Xenobiotic Metabolism AHR Signaling Pathway**	0.044	0.06	0.037		2E-4	0.017	0.020	0.06	0.05	0.10		0.11	0.017	0.017
Glioma Signaling	0.07	0.004	0.06		0.16	0.026	0.031	0.004	0.003	0.14		0.014	0.07	0.09
**Bladder Cancer Signaling**	0.06	0.08	0.049		0.14	0.022	0.027	0.08	0.07	0.12		0.15	0.022	0.022
Hereditary Breast Cancer Signaling	0.07	0.10	0.06		0.18	0.028	0.034	0.10	0.003	0.15	0.09	0.001	0.08	0.10
Ovarian Cancer Signaling	0.08	0.11	0.07		0.19	0.031	0.037	0.11	0.004	0.17		7E-5	0.09	0.11
**Apelin Endothelial Signaling Pathway**	0.07	0.10	0.06	0.07	0.18	0.029	0.035	0.11	0.003	0.16		0.19	0.08	0.005
Coronavirus Pathogenesis Pathway	0.10	0.13	0.09		0.030	0.040	0.047	0.14	0.11	0.21		0.25	0.11	0.009
**Adipogenesis pathway**	0.07	0.10	0.06		0.17	0.027	0.032	0.005	0.08	0.15	0.09	0.18	0.07	0.10
**Granzyme A Signaling**			0.032				0.018							
**Mitochondrial Dysfunction**	0.09	0.13	0.003				0.044							
Vitamin-C Transport										0.037	0.022			0.023
**Tumoricidal Function of Hepatic Natural Killer Cells**								0.018		0.027				0.017
**Agrin Interactions at Neuromuscular Junction**						0.014							0.014	0.014
**CDK5 Signaling**		0.08				0.023						0.15	0.023	0.023
**Coenzyme A Biosynthesis**										0.016			0.008	
**Complement System**										0.043				0.026
**Heme Biosynthesis from Uroporphyrinogen-III I**										7E-5				0.008
**Heme Biosynthesis II**										3E-4				0.016
Huntington's Disease Signaling	0.009	0.017	0.12	0.14	0.32	0.05	0.002	0.19	2E-5	0.004		0.06	0.15	0.19
Calcium Signaling	0.11	0.15	0.09		0.26	0.044	0.05	0.012	0.13	0.028		0.039	0.12	0.15
EGF Signaling		0.042	0.026						0.035			0.08		0.042
GM-CSF Signaling		0.049							0.041					0.049
Melanoma Signaling				0.026				0.038	0.030					
Molecular Mechanisms of Cancer	0.21	0.041	0.18	0.21	0.13	0.09	0.10	0.046	0.029	0.41		0.13	0.22	0.28
Semaphorin Signaling in Neurons		0.043						0.045	0.035					
Amyloid Processing		0.036	0.022						0.030			0.07		
IL-2 Signaling		0.045	0.028						0.037					
Role of JAK1 and JAK3 in γc Cytokine Signaling	0.035	0.048												0.048

P values of recurrent Ingenuity Canonical Pathways in individual cell lines or their combination. Pathways more specific for D3 cell lines are depicted in blue and pathways more specific for M3 cell lines are depicted in red.

References

1 Mergener, S., Siveke, J. T. & Peña-Llopis, S. Monosomy 3 Is Linked to Resistance to MEK Inhibitors in Uveal Melanoma. *Int J Mol Sci* **22**, doi:10.3390/ijms22136727 (2021).

2 Di Veroli, G. Y. *et al.* Combenefit: an interactive platform for the analysis and visualization of drug combinations. *Bioinformatics* **32**, 2866-2868, doi:10.1093/bioinformatics/btw230 (2016).

3 Nemetski, S. M. & Gardner, L. B. Hypoxic Regulation of Id-1 and Activation of the Unfolded Protein Response Are Aberrant in Neuroblastoma *. *Journal of Biological Chemistry* **282**, 240-248, doi:10.1074/jbc.M607275200 (2007).
